# Supplementary figures and images for: Centrosomal actin pool levels regulated by localized PKA set the threshold for T cell polarization (part 1 of 2)
Source: EMBO Rep. 2025 Aug 26;26(18):4436–55. doi: 10.1038/s44319-025-00533-2 (PMC12457651; doi:10.1038/s44319-025-00533-2)

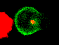

Supplement: Supplementary file 2 — Source data Fig. 1 [file 44319_2025_533_MOESM2_ESM.zip › Figure 1/Figure 1C/Centrin actin +340.tif]

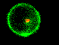

Supplement: Supplementary file 2 — Source data Fig. 1 [file 44319_2025_533_MOESM2_ESM.zip › Figure 1/Figure 1C/Centrin actin -190.tif]

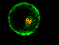

Supplement: Supplementary file 2 — Source data Fig. 1 [file 44319_2025_533_MOESM2_ESM.zip › Figure 1/Figure 1C/Centrin actin -20.tif]

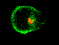

Supplement: Supplementary file 2 — Source data Fig. 1 [file 44319_2025_533_MOESM2_ESM.zip › Figure 1/Figure 1C/Centrin actin+210.tif]

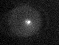

Supplement: Supplementary file 2 — Source data Fig. 1 [file 44319_2025_533_MOESM2_ESM.zip › Figure 1/Figure 1C/Original movie Centrin actin.tif]

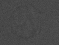

Supplement: Supplementary file 2 — Source data Fig. 1 [file 44319_2025_533_MOESM2_ESM.zip › Figure 1/Figure 1C/Original movie Visible.tif]

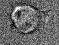

Supplement: Supplementary file 2 — Source data Fig. 1 [file 44319_2025_533_MOESM2_ESM.zip › Figure 1/Figure 1C/visible +210.tif]

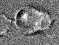

Supplement: Supplementary file 2 — Source data Fig. 1 [file 44319_2025_533_MOESM2_ESM.zip › Figure 1/Figure 1C/visible +340.tif]

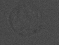

Supplement: Supplementary file 2 — Source data Fig. 1 [file 44319_2025_533_MOESM2_ESM.zip › Figure 1/Figure 1C/visible -190.tif]

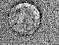

Supplement: Supplementary file 2 — Source data Fig. 1 [file 44319_2025_533_MOESM2_ESM.zip › Figure 1/Figure 1C/visible -20.tif]

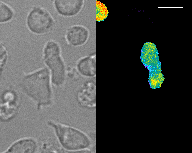

Supplement: Supplementary file 3 — Source data Fig. 2 [file 44319_2025_533_MOESM3_ESM.zip › Figure 2/Figure 2D/cAMP +160.tif]

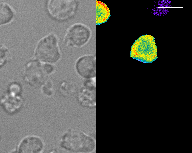

Supplement: Supplementary file 3 — Source data Fig. 2 [file 44319_2025_533_MOESM3_ESM.zip › Figure 2/Figure 2D/cAMP +40.tif]

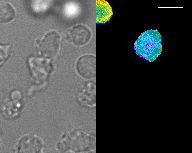

Supplement: Supplementary file 3 — Source data Fig. 2 [file 44319_2025_533_MOESM3_ESM.zip › Figure 2/Figure 2D/cAMP -10.tif]

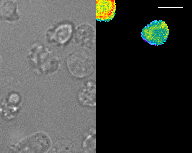

Supplement: Supplementary file 3 — Source data Fig. 2 [file 44319_2025_533_MOESM3_ESM.zip › Figure 2/Figure 2D/Original movie cAMP.tif]

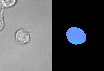

Supplement: Supplementary file 3 — Source data Fig. 2 [file 44319_2025_533_MOESM3_ESM.zip › Figure 2/Figure 2D/Original movie PKA.tif]

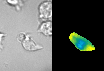

Supplement: Supplementary file 3 — Source data Fig. 2 [file 44319_2025_533_MOESM3_ESM.zip › Figure 2/Figure 2D/PKA +190.tif]

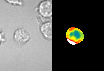

Supplement: Supplementary file 3 — Source data Fig. 2 [file 44319_2025_533_MOESM3_ESM.zip › Figure 2/Figure 2D/PKA +40.tif]

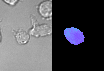

Supplement: Supplementary file 3 — Source data Fig. 2 [file 44319_2025_533_MOESM3_ESM.zip › Figure 2/Figure 2D/PKA -10.tif]

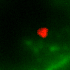

Supplement: Supplementary file 4 — Source data Fig. 3 [file 44319_2025_533_MOESM4_ESM.zip › Figure 3/Figure 3B/Control Actin and Pericentrin zoom.tif]

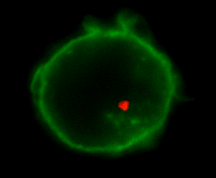

Supplement: Supplementary file 4 — Source data Fig. 3 [file 44319_2025_533_MOESM4_ESM.zip › Figure 3/Figure 3B/Control Actin and Pericentrin.tif]

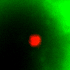

Supplement: Supplementary file 4 — Source data Fig. 3 [file 44319_2025_533_MOESM4_ESM.zip › Figure 3/Figure 3B/CXCL12 Actin and Pericentrin zoom.tif]

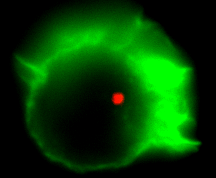

Supplement: Supplementary file 4 — Source data Fig. 3 [file 44319_2025_533_MOESM4_ESM.zip › Figure 3/Figure 3B/CXCL12 Actin and Pericentrin.tif]

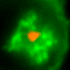

Supplement: Supplementary file 4 — Source data Fig. 3 [file 44319_2025_533_MOESM4_ESM.zip › Figure 3/Figure 3B/H89 Actin and Pericentrin zoom.tif]

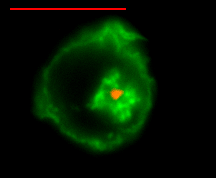

Supplement: Supplementary file 4 — Source data Fig. 3 [file 44319_2025_533_MOESM4_ESM.zip › Figure 3/Figure 3B/H89 Actin and Pericentrin.tif]

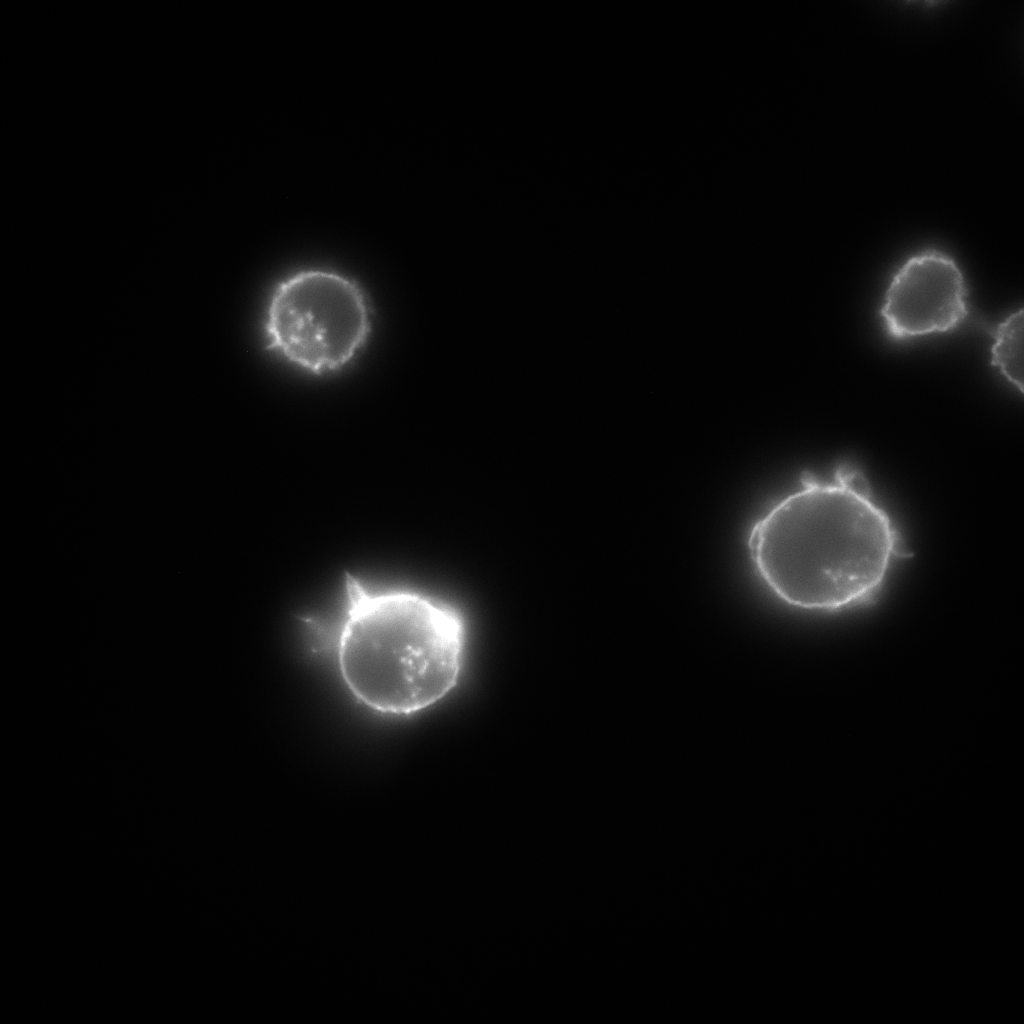

Supplement: Supplementary file 4 — Source data Fig. 3 [file 44319_2025_533_MOESM4_ESM.zip › Figure 3/Figure 3B/Original image Control Actin.tif]

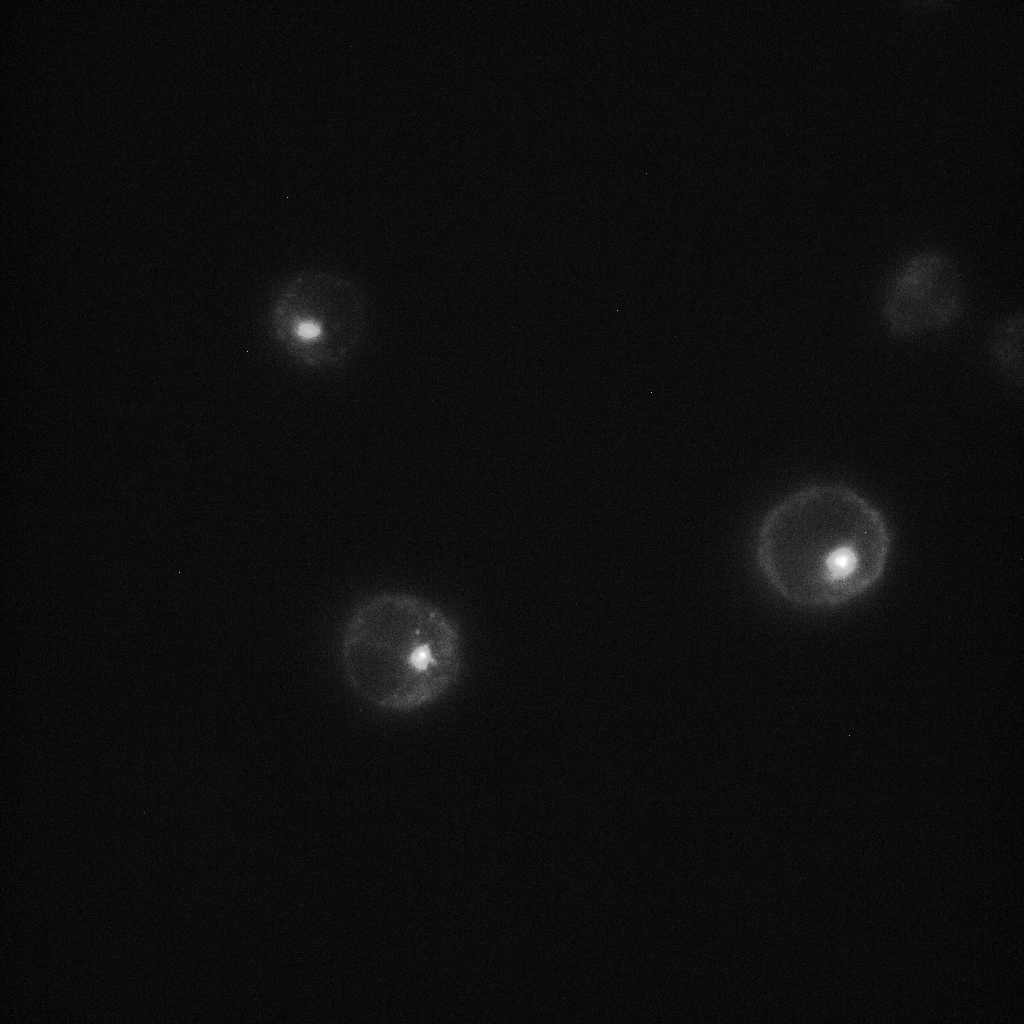

Supplement: Supplementary file 4 — Source data Fig. 3 [file 44319_2025_533_MOESM4_ESM.zip › Figure 3/Figure 3B/Original image Control Pericentrin.tif]

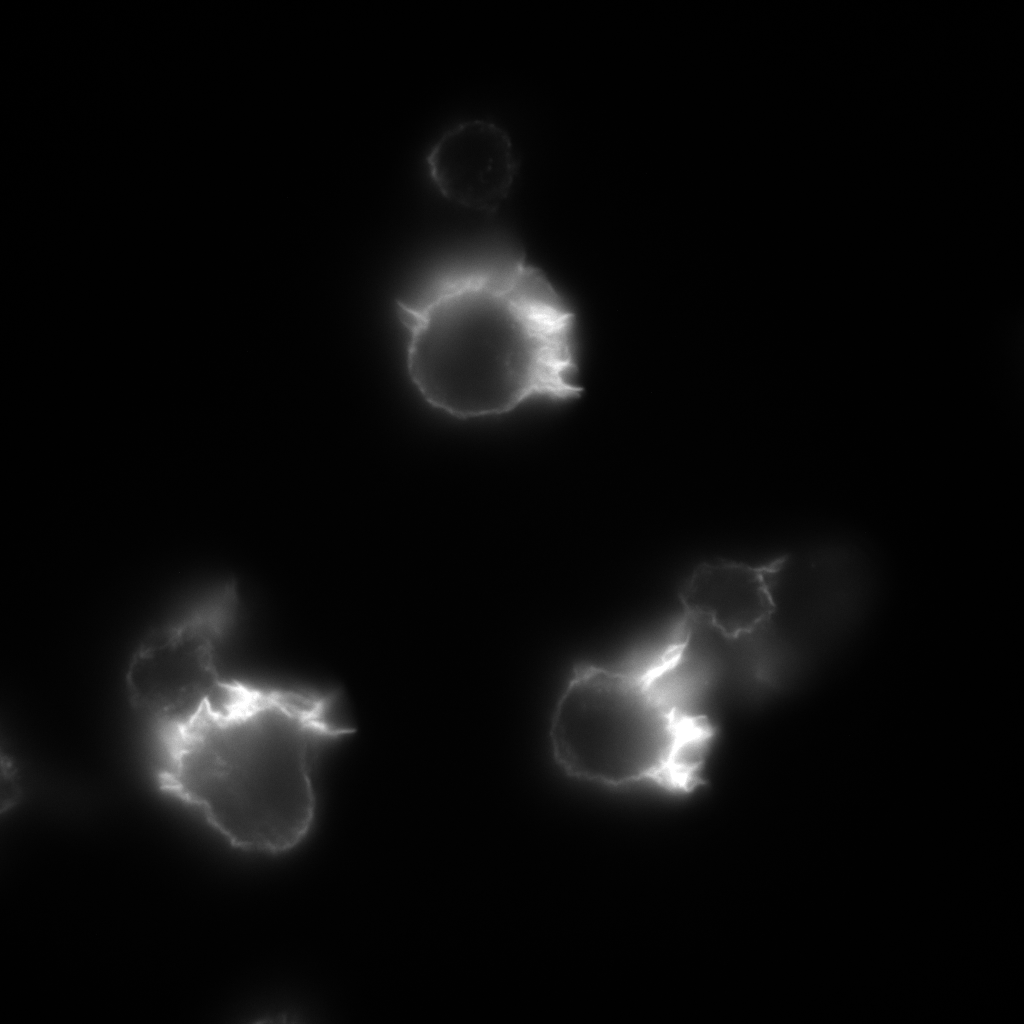

Supplement: Supplementary file 4 — Source data Fig. 3 [file 44319_2025_533_MOESM4_ESM.zip › Figure 3/Figure 3B/Original image CXCL12 Actin.tif]

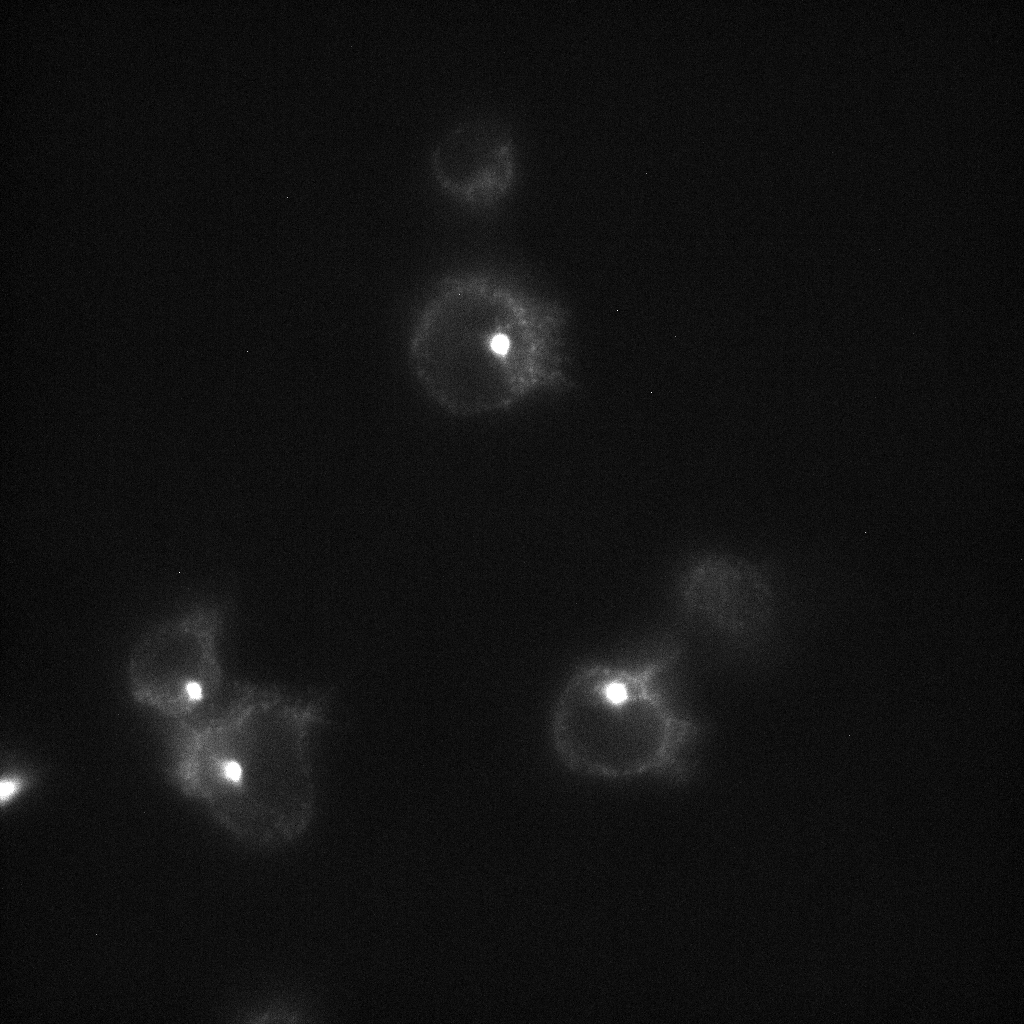

Supplement: Supplementary file 4 — Source data Fig. 3 [file 44319_2025_533_MOESM4_ESM.zip › Figure 3/Figure 3B/Original image CXCL12 Pericentrin.tif]

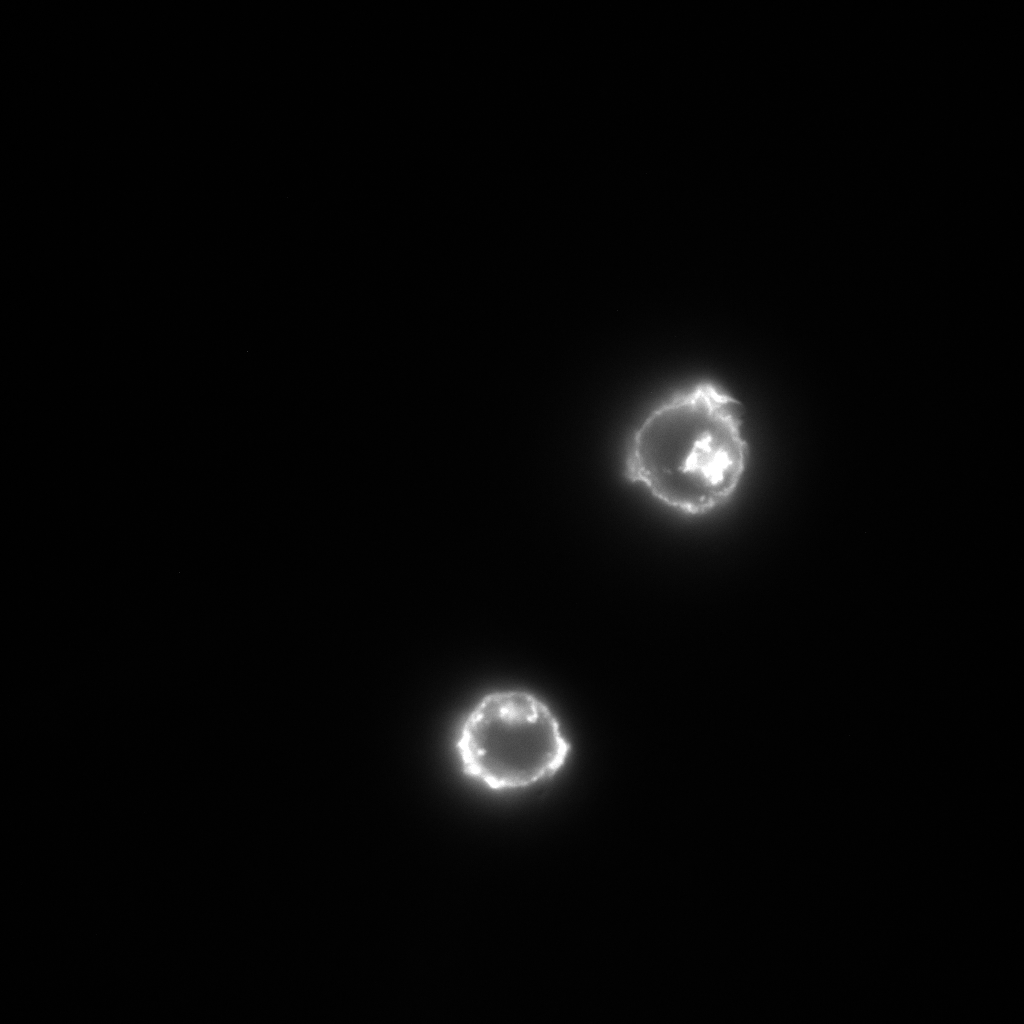

Supplement: Supplementary file 4 — Source data Fig. 3 [file 44319_2025_533_MOESM4_ESM.zip › Figure 3/Figure 3B/Original image H89 Actin.tif]

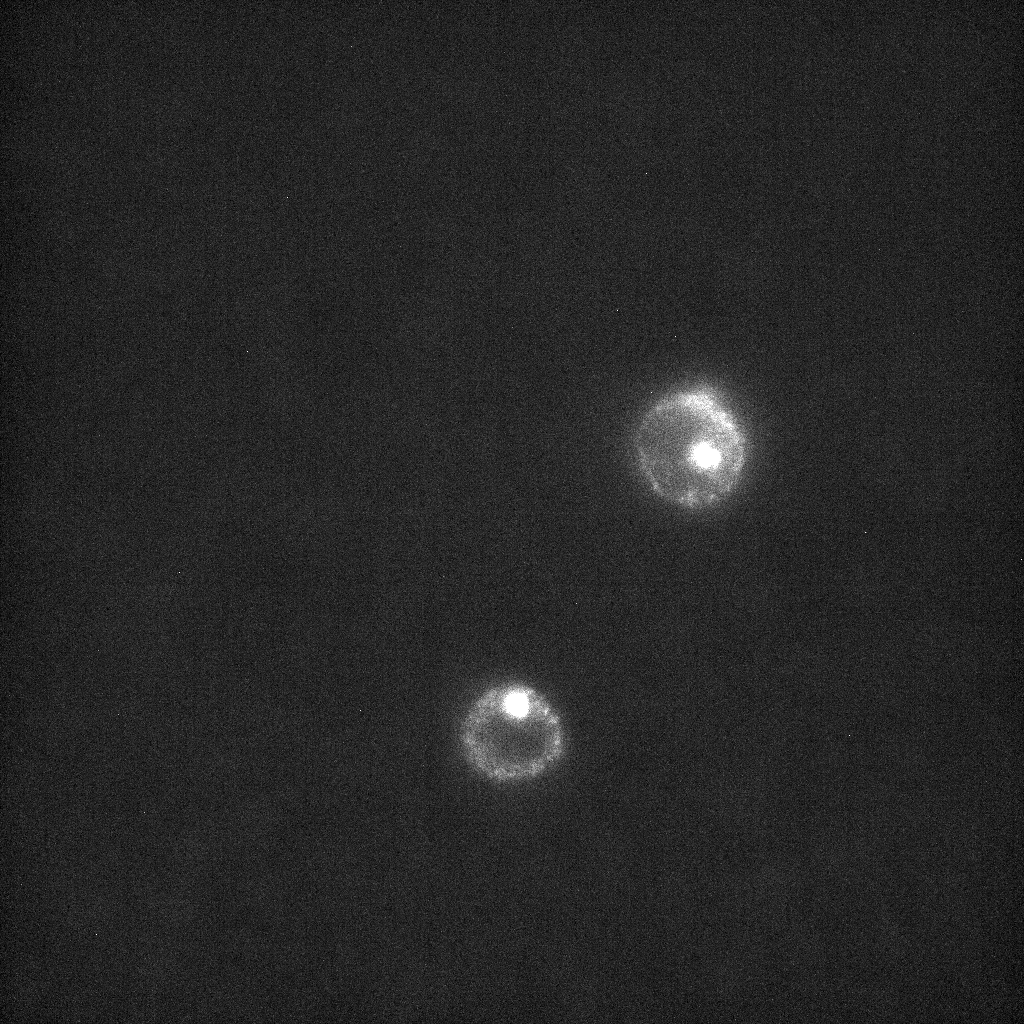

Supplement: Supplementary file 4 — Source data Fig. 3 [file 44319_2025_533_MOESM4_ESM.zip › Figure 3/Figure 3B/Original image H89 Pericentrin.tif]

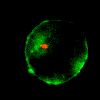

Supplement: Supplementary file 4 — Source data Fig. 3 [file 44319_2025_533_MOESM4_ESM.zip › Figure 3/Figure 3E/Actin and Centrin -10.tif]

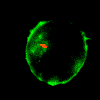

Supplement: Supplementary file 4 — Source data Fig. 3 [file 44319_2025_533_MOESM4_ESM.zip › Figure 3/Figure 3E/Actin and Centrin +20.tif]

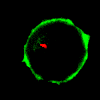

Supplement: Supplementary file 4 — Source data Fig. 3 [file 44319_2025_533_MOESM4_ESM.zip › Figure 3/Figure 3E/Actin and Centrin +40.tif]

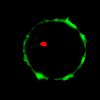

Supplement: Supplementary file 4 — Source data Fig. 3 [file 44319_2025_533_MOESM4_ESM.zip › Figure 3/Figure 3E/Actin and Centrin +70.tif]

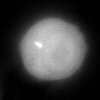

Supplement: Supplementary file 4 — Source data Fig. 3 [file 44319_2025_533_MOESM4_ESM.zip › Figure 3/Figure 3E/Original movie Actin and Centrin.tif]

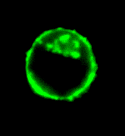

Supplement: Supplementary file 5 — Source data Fig. 4 [file 44319_2025_533_MOESM5_ESM.zip › Figure 4/Figure 4A/Actin.tif]

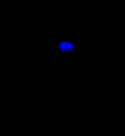

Supplement: Supplementary file 5 — Source data Fig. 4 [file 44319_2025_533_MOESM5_ESM.zip › Figure 4/Figure 4A/AKAP450.tif]

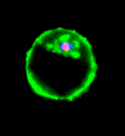

Supplement: Supplementary file 5 — Source data Fig. 4 [file 44319_2025_533_MOESM5_ESM.zip › Figure 4/Figure 4A/merge.tif]

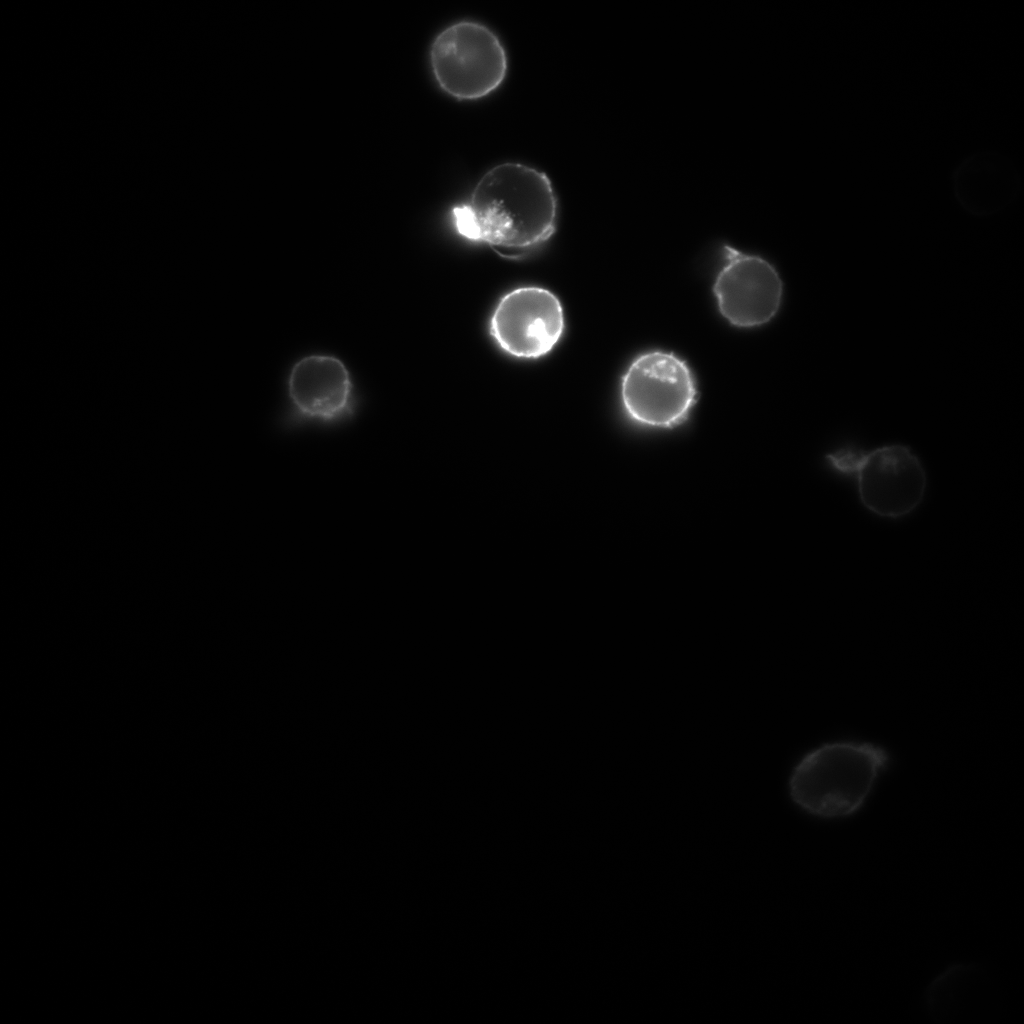

Supplement: Supplementary file 5 — Source data Fig. 4 [file 44319_2025_533_MOESM5_ESM.zip › Figure 4/Figure 4A/Original Image Actin.tif]

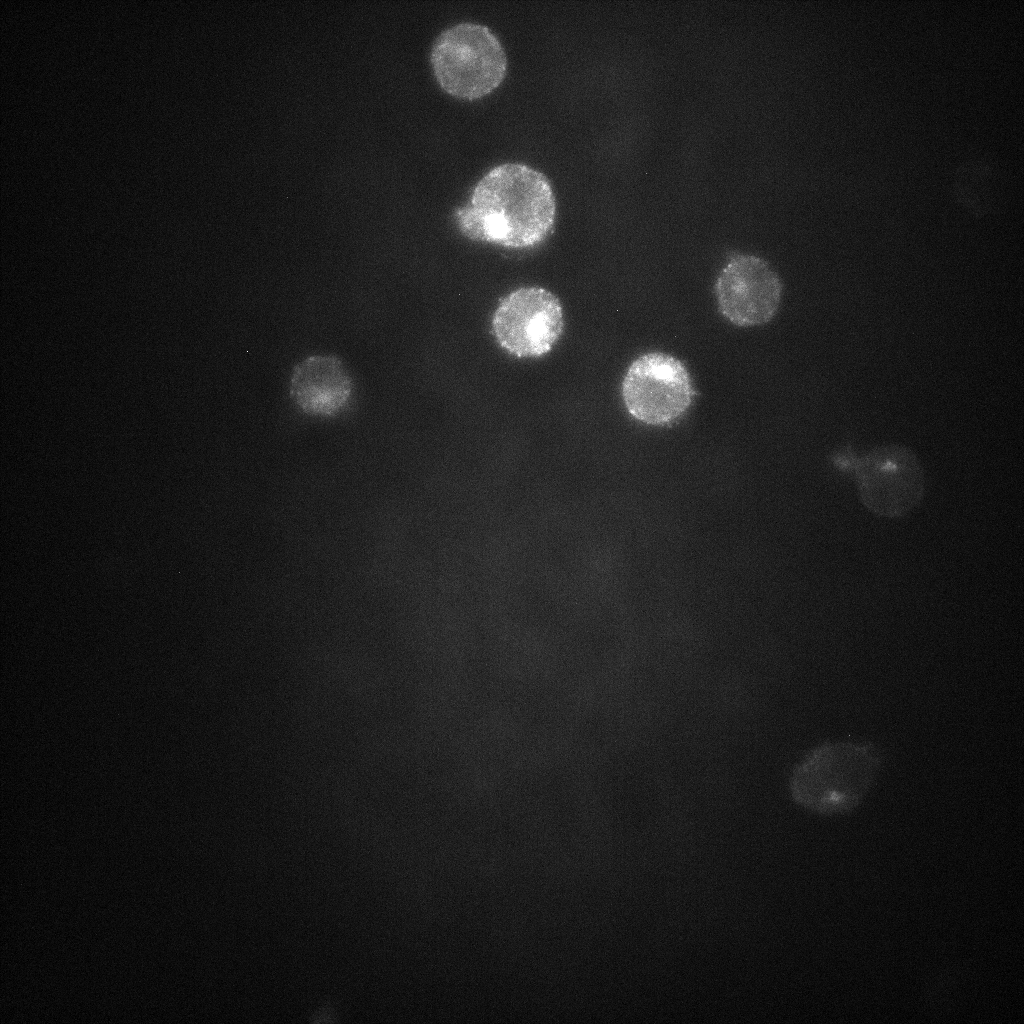

Supplement: Supplementary file 5 — Source data Fig. 4 [file 44319_2025_533_MOESM5_ESM.zip › Figure 4/Figure 4A/Original Image AKAP450.tif]

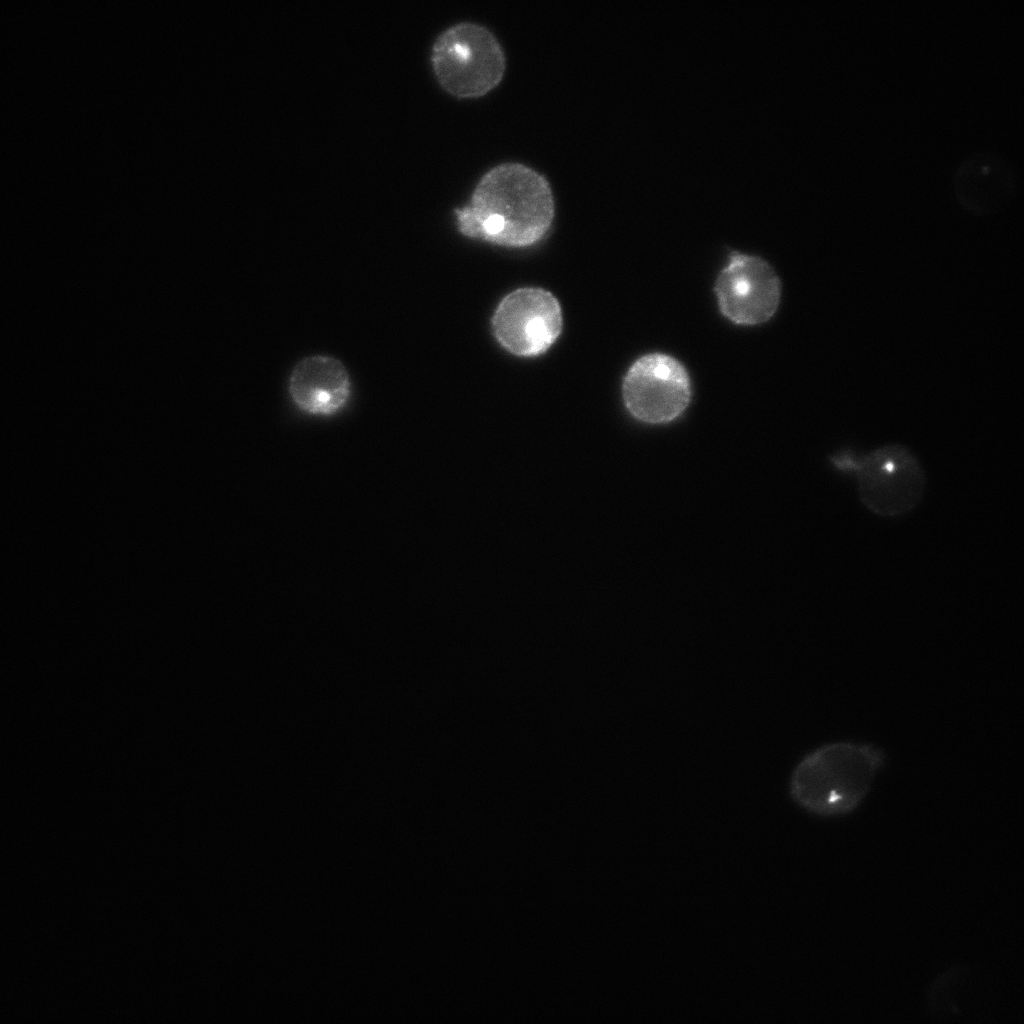

Supplement: Supplementary file 5 — Source data Fig. 4 [file 44319_2025_533_MOESM5_ESM.zip › Figure 4/Figure 4A/Original Image Pericentrin.tif]

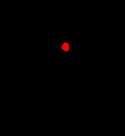

Supplement: Supplementary file 5 — Source data Fig. 4 [file 44319_2025_533_MOESM5_ESM.zip › Figure 4/Figure 4A/Pericentrin.tif]

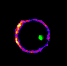

Supplement: Supplementary file 7 — Figure EV1 Source Data [file 44319_2025_533_MOESM7_ESM.zip › Figure EV1/Figure EV1A/Control CXCR4 and Pericentrin cell4.tif]

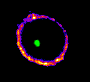

Supplement: Supplementary file 7 — Figure EV1 Source Data [file 44319_2025_533_MOESM7_ESM.zip › Figure EV1/Figure EV1A/Control CXCR4 and Pericentrin cell1.tif]

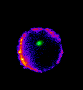

Supplement: Supplementary file 7 — Figure EV1 Source Data [file 44319_2025_533_MOESM7_ESM.zip › Figure EV1/Figure EV1A/Control CXCR4 and Pericentrin cell2.tif]

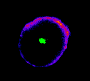

Supplement: Supplementary file 7 — Figure EV1 Source Data [file 44319_2025_533_MOESM7_ESM.zip › Figure EV1/Figure EV1A/Control CXCR4 and Pericentrin cell3.tif]

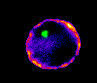

Supplement: Supplementary file 7 — Figure EV1 Source Data [file 44319_2025_533_MOESM7_ESM.zip › Figure EV1/Figure EV1A/Control CXCR4 and Pericentrin cell5.tif]

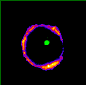

Supplement: Supplementary file 7 — Figure EV1 Source Data [file 44319_2025_533_MOESM7_ESM.zip › Figure EV1/Figure EV1A/Control CXCR4 and Pericentrin cell6.tif]

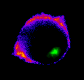

Supplement: Supplementary file 7 — Figure EV1 Source Data [file 44319_2025_533_MOESM7_ESM.zip › Figure EV1/Figure EV1A/Control CXCR4 and Pericentrin cell7.tif]

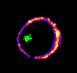

Supplement: Supplementary file 7 — Figure EV1 Source Data [file 44319_2025_533_MOESM7_ESM.zip › Figure EV1/Figure EV1A/Control CXCR4 and Pericentrin cell8.tif]

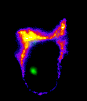

Supplement: Supplementary file 7 — Figure EV1 Source Data [file 44319_2025_533_MOESM7_ESM.zip › Figure EV1/Figure EV1A/CXCL12 CXCR4 and Pericentrin cell10.tif]

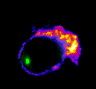

Supplement: Supplementary file 7 — Figure EV1 Source Data [file 44319_2025_533_MOESM7_ESM.zip › Figure EV1/Figure EV1A/CXCL12 CXCR4 and Pericentrin cell11.tif]

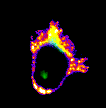

Supplement: Supplementary file 7 — Figure EV1 Source Data [file 44319_2025_533_MOESM7_ESM.zip › Figure EV1/Figure EV1A/CXCL12 CXCR4 and Pericentrin cell12.tif]

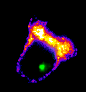

Supplement: Supplementary file 7 — Figure EV1 Source Data [file 44319_2025_533_MOESM7_ESM.zip › Figure EV1/Figure EV1A/CXCL12 CXCR4 and Pericentrin cell13.tif]

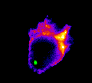

Supplement: Supplementary file 7 — Figure EV1 Source Data [file 44319_2025_533_MOESM7_ESM.zip › Figure EV1/Figure EV1A/CXCL12 CXCR4 and Pericentrin cell9.tif]

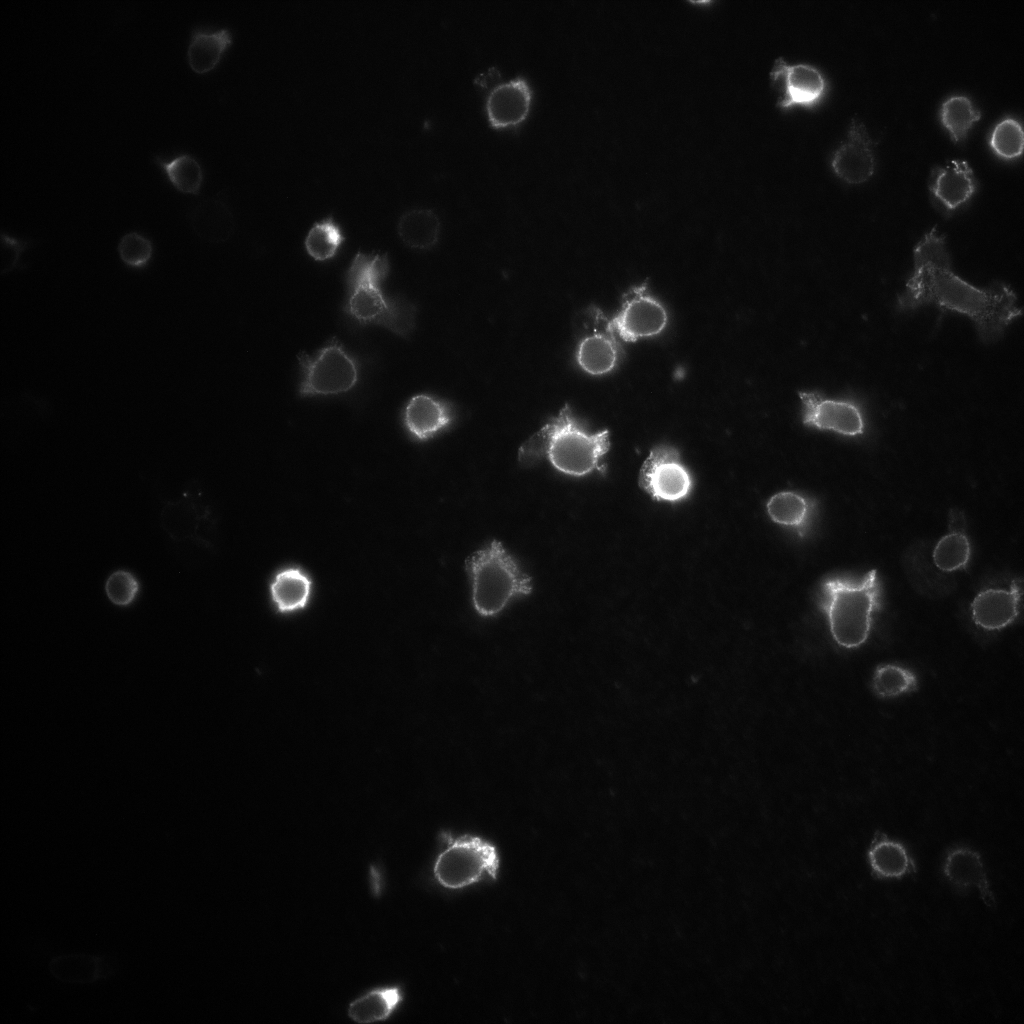

Supplement: Supplementary file 7 — Figure EV1 Source Data [file 44319_2025_533_MOESM7_ESM.zip › Figure EV1/Figure EV1A/Original image CXCL12 CXCR4 cell10,11,12.tif]

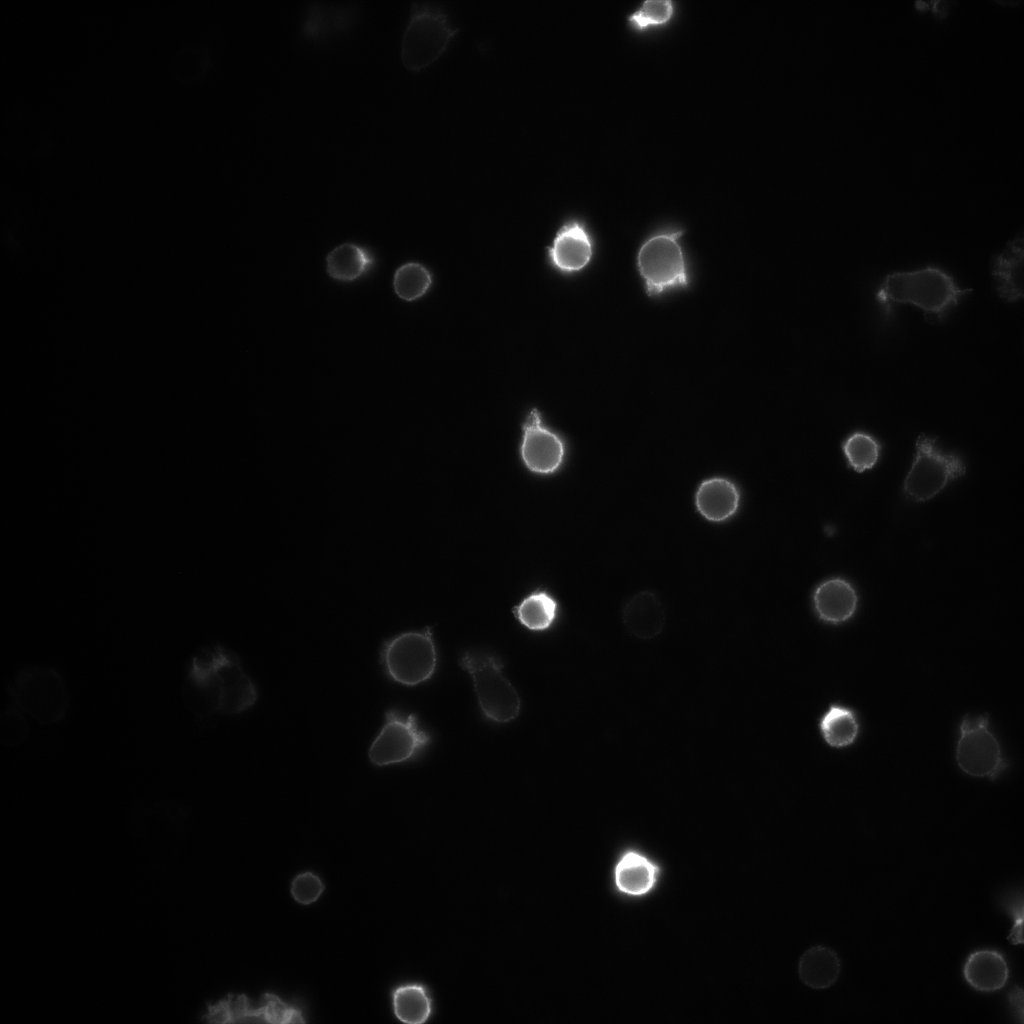

Supplement: Supplementary file 7 — Figure EV1 Source Data [file 44319_2025_533_MOESM7_ESM.zip › Figure EV1/Figure EV1A/Original image CXCL12 CXCR4 cell13.tif]

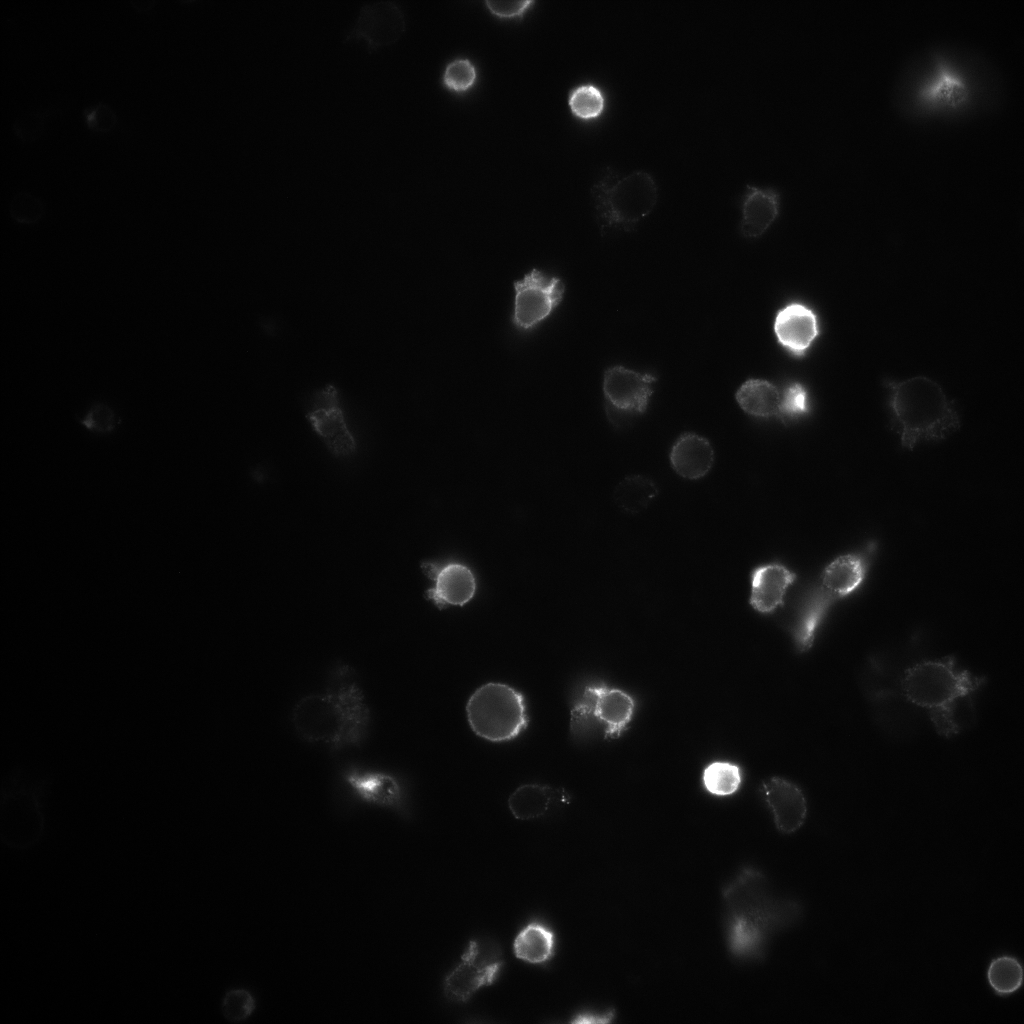

Supplement: Supplementary file 7 — Figure EV1 Source Data [file 44319_2025_533_MOESM7_ESM.zip › Figure EV1/Figure EV1A/Original image CXCL12 CXCR4 cell9.tif]

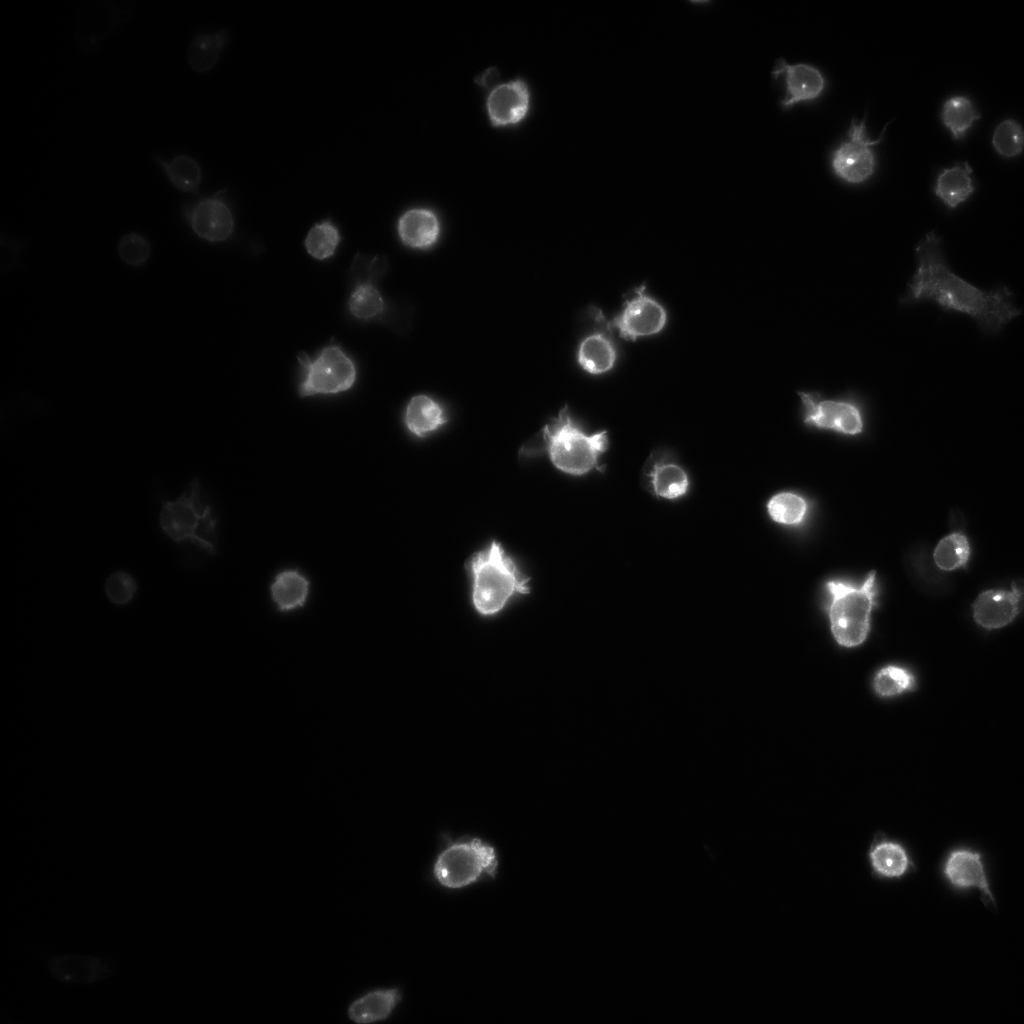

Supplement: Supplementary file 7 — Figure EV1 Source Data [file 44319_2025_533_MOESM7_ESM.zip › Figure EV1/Figure EV1A/Original image CXCL12 Pericentrin cell10,11,12.tif]

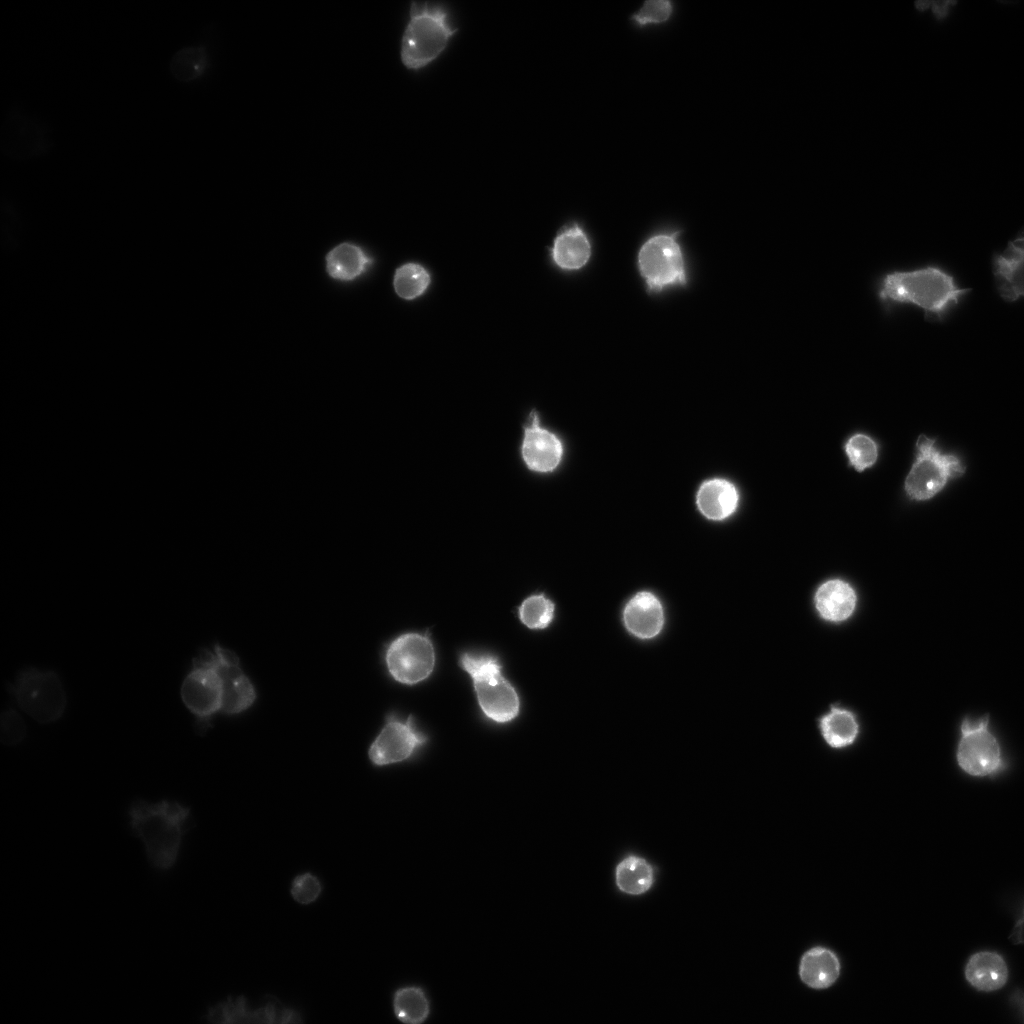

Supplement: Supplementary file 7 — Figure EV1 Source Data [file 44319_2025_533_MOESM7_ESM.zip › Figure EV1/Figure EV1A/Original image CXCL12 Pericentrin cell13.tif]

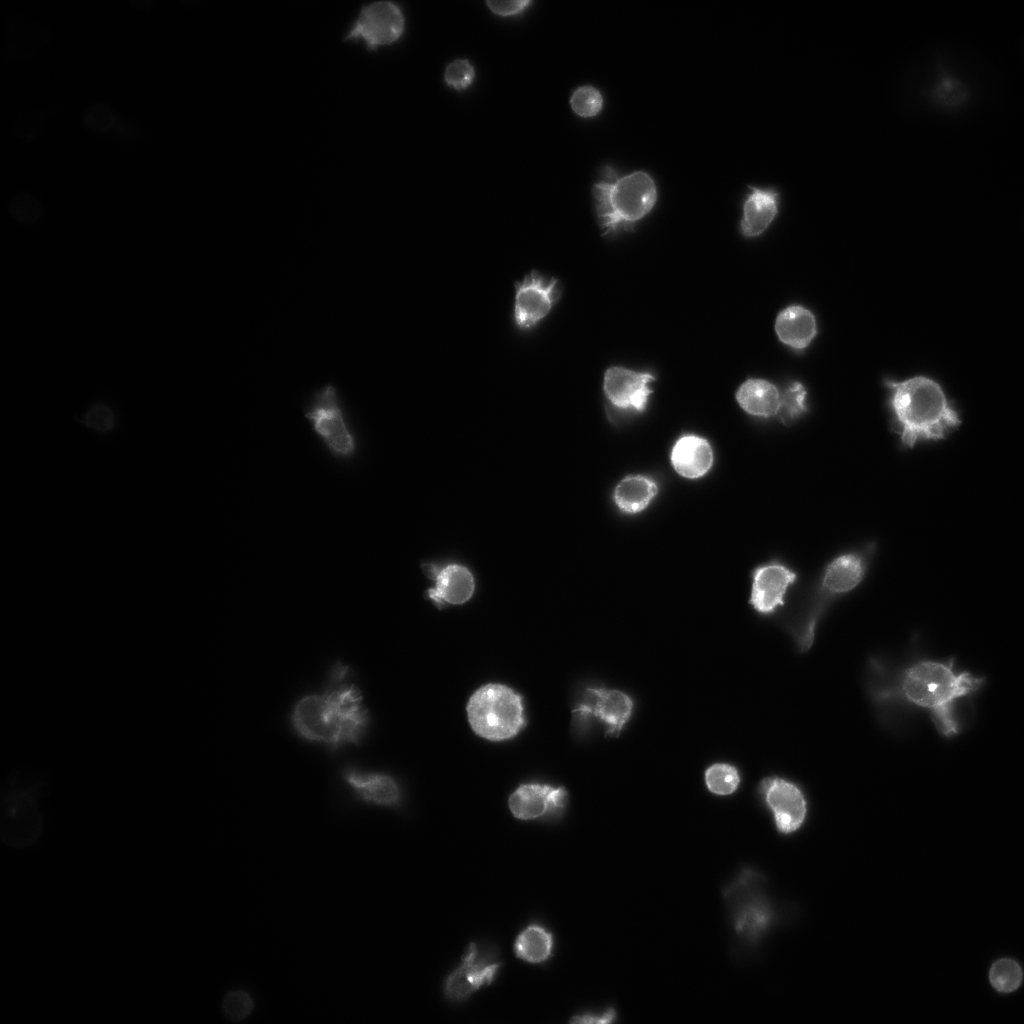

Supplement: Supplementary file 7 — Figure EV1 Source Data [file 44319_2025_533_MOESM7_ESM.zip › Figure EV1/Figure EV1A/Original image CXCL12 Pericentrin cell9.tif]

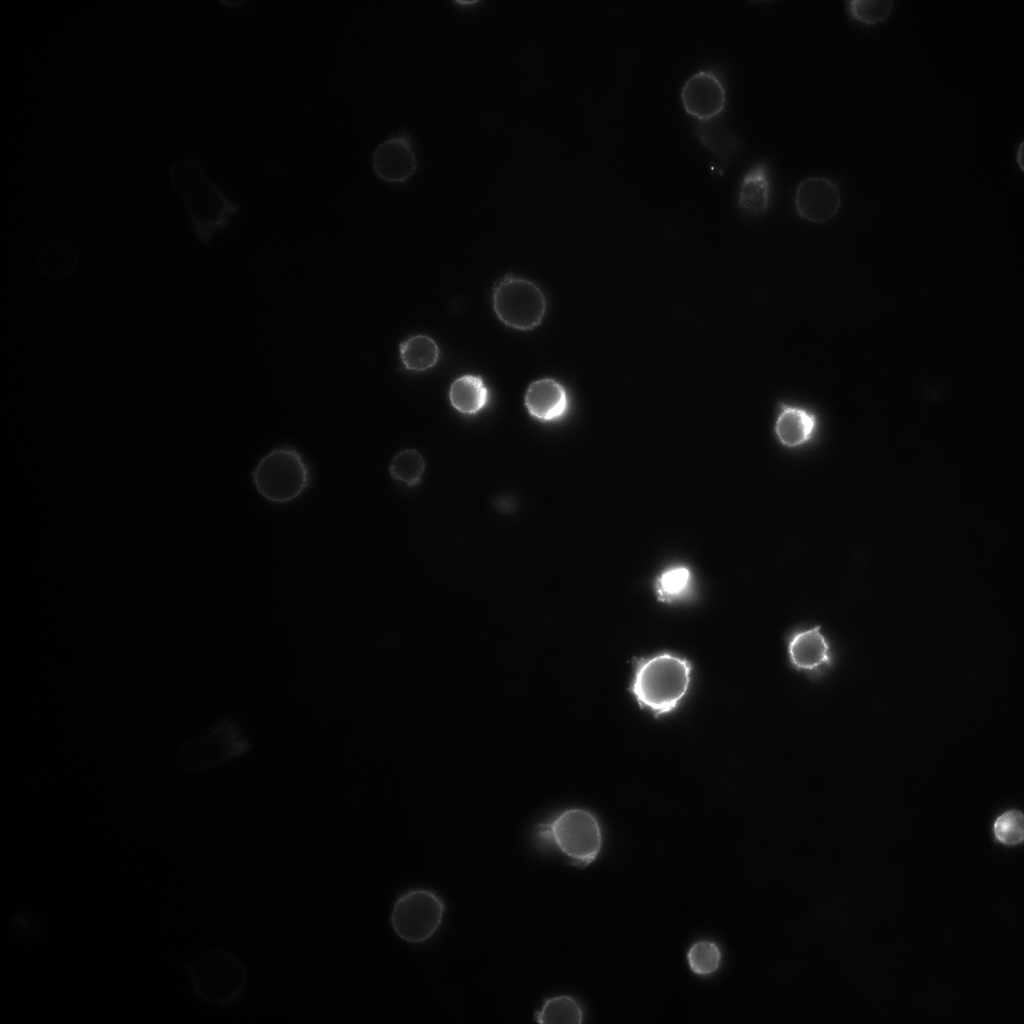

Supplement: Supplementary file 7 — Figure EV1 Source Data [file 44319_2025_533_MOESM7_ESM.zip › Figure EV1/Figure EV1A/Original image CXCR4 cell1,2,3.tif]

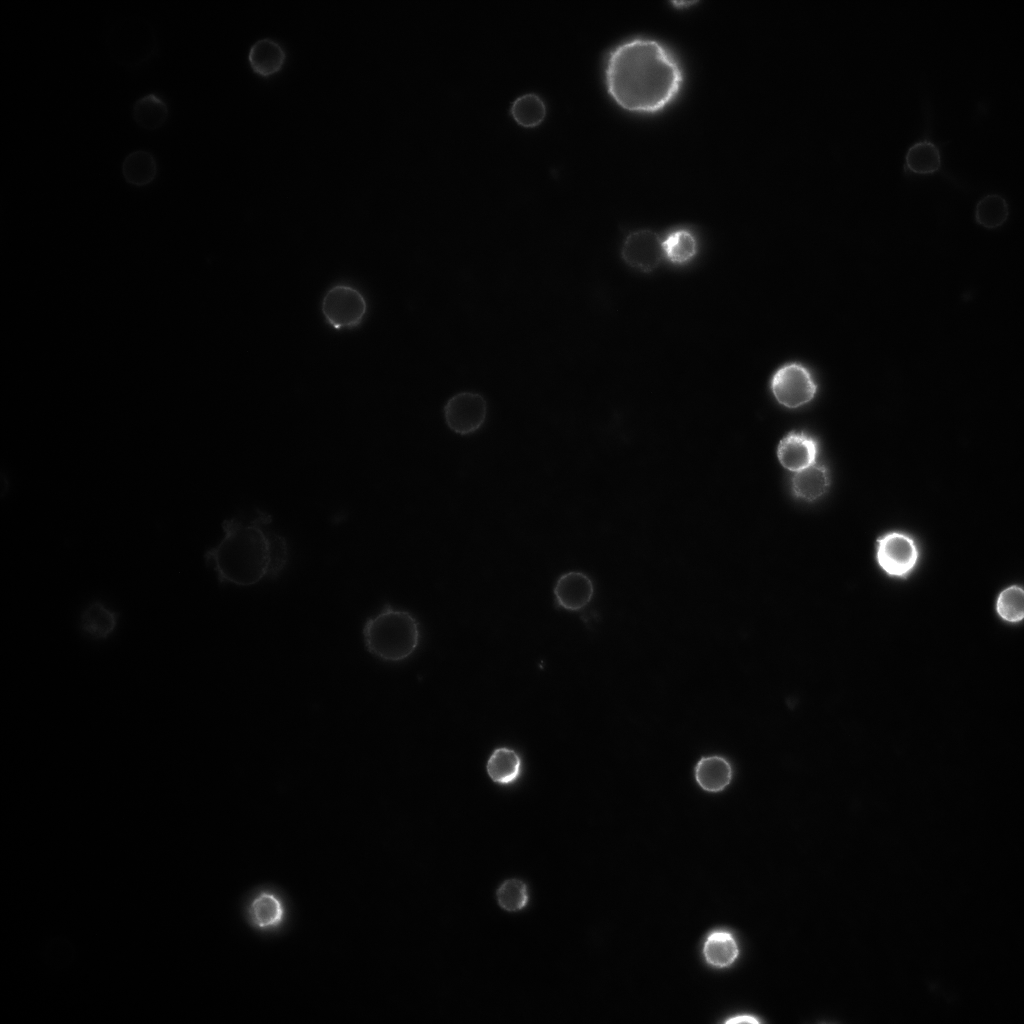

Supplement: Supplementary file 7 — Figure EV1 Source Data [file 44319_2025_533_MOESM7_ESM.zip › Figure EV1/Figure EV1A/Original image CXCR4 cell4.tif]

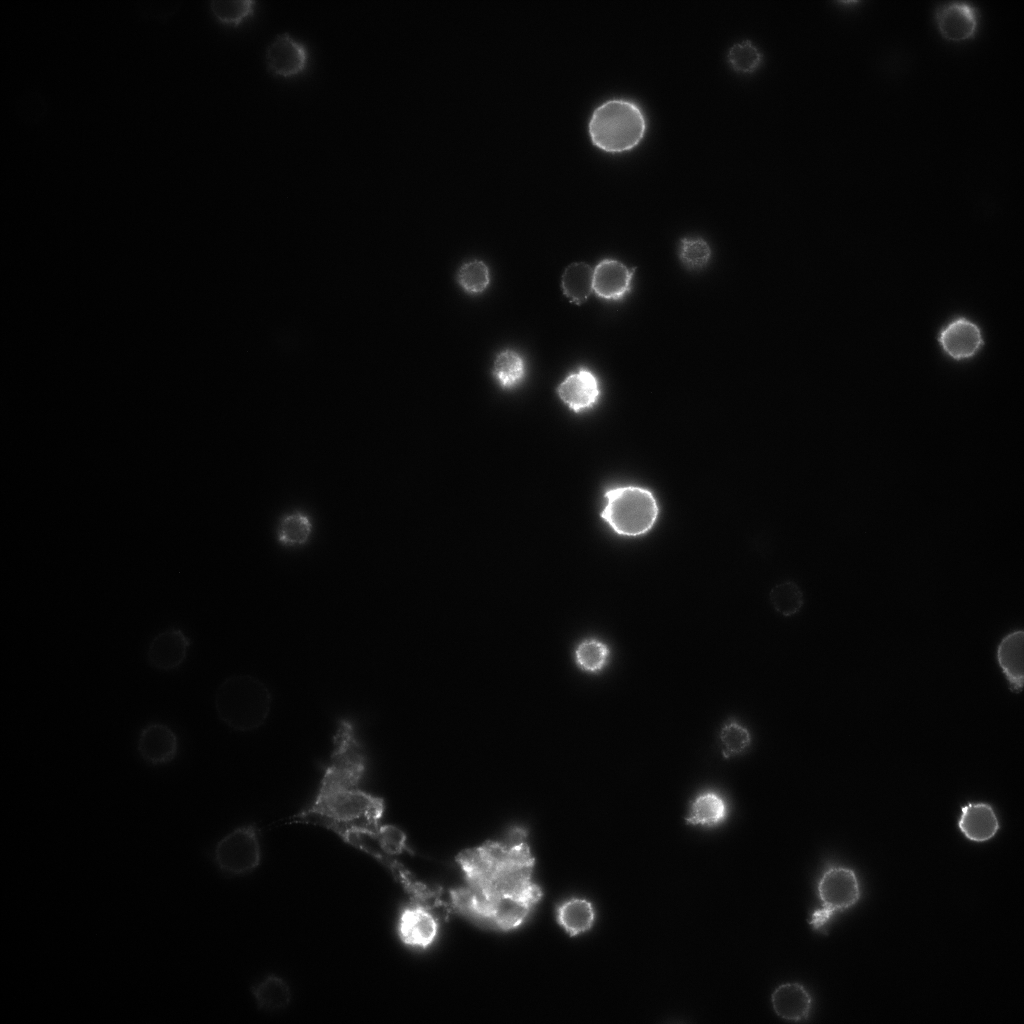

Supplement: Supplementary file 7 — Figure EV1 Source Data [file 44319_2025_533_MOESM7_ESM.zip › Figure EV1/Figure EV1A/Original image CXCR4 cell5.tif]

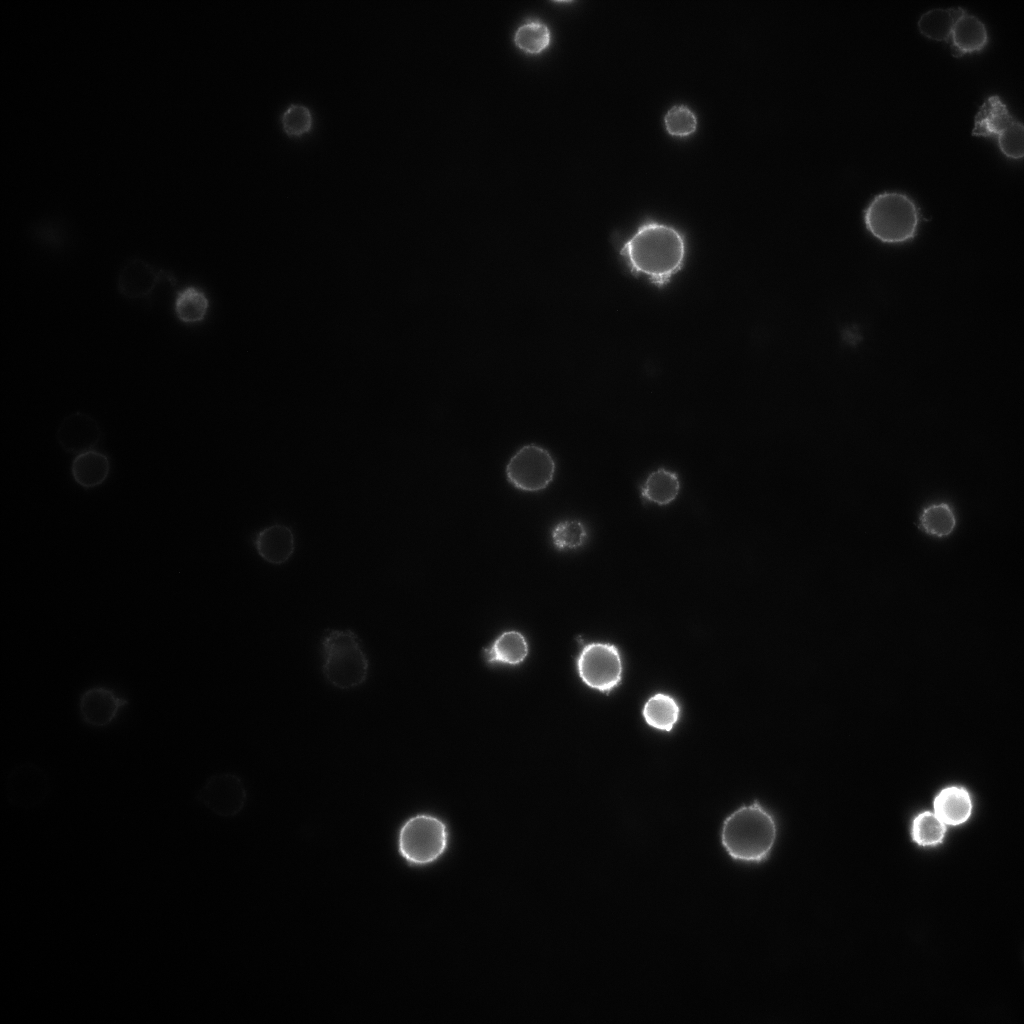

Supplement: Supplementary file 7 — Figure EV1 Source Data [file 44319_2025_533_MOESM7_ESM.zip › Figure EV1/Figure EV1A/Original image CXCR4 cell6.tif]

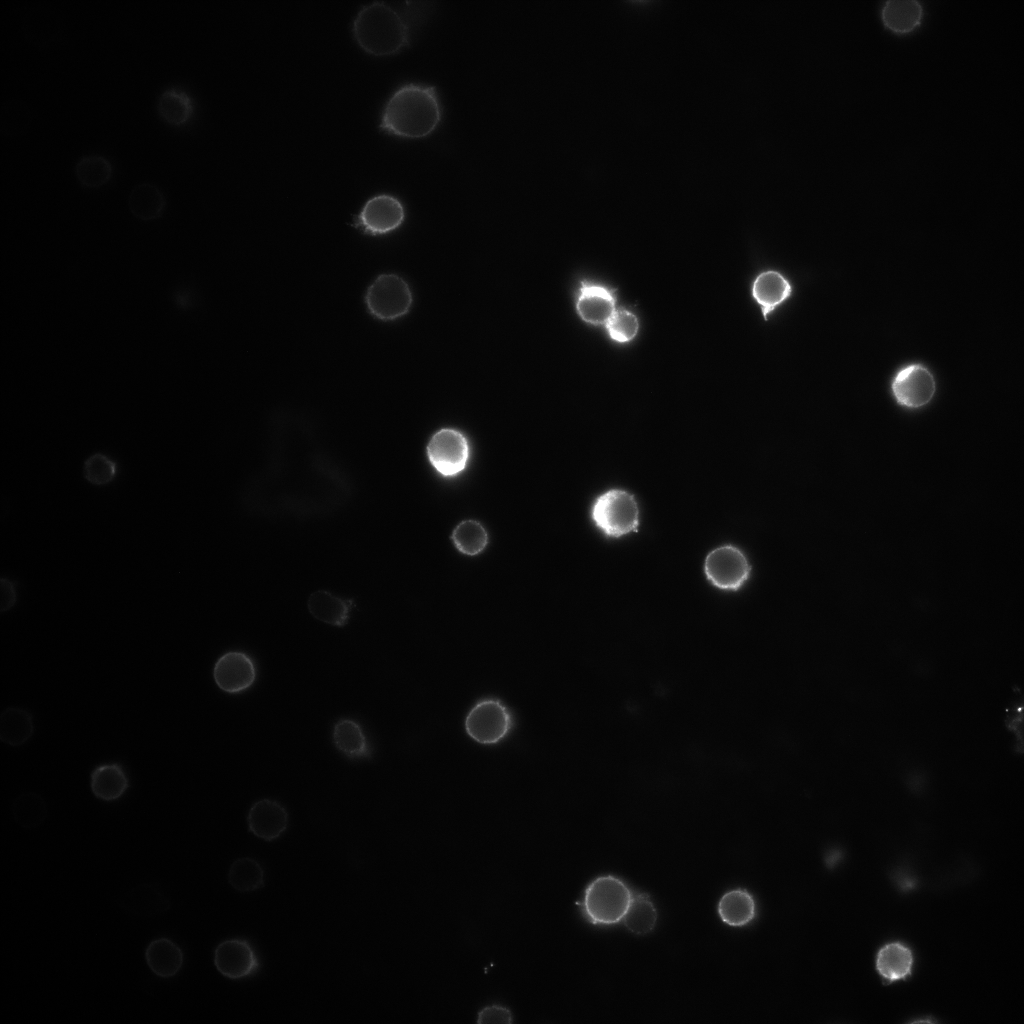

Supplement: Supplementary file 7 — Figure EV1 Source Data [file 44319_2025_533_MOESM7_ESM.zip › Figure EV1/Figure EV1A/Original image CXCR4 cell7,8.tif]

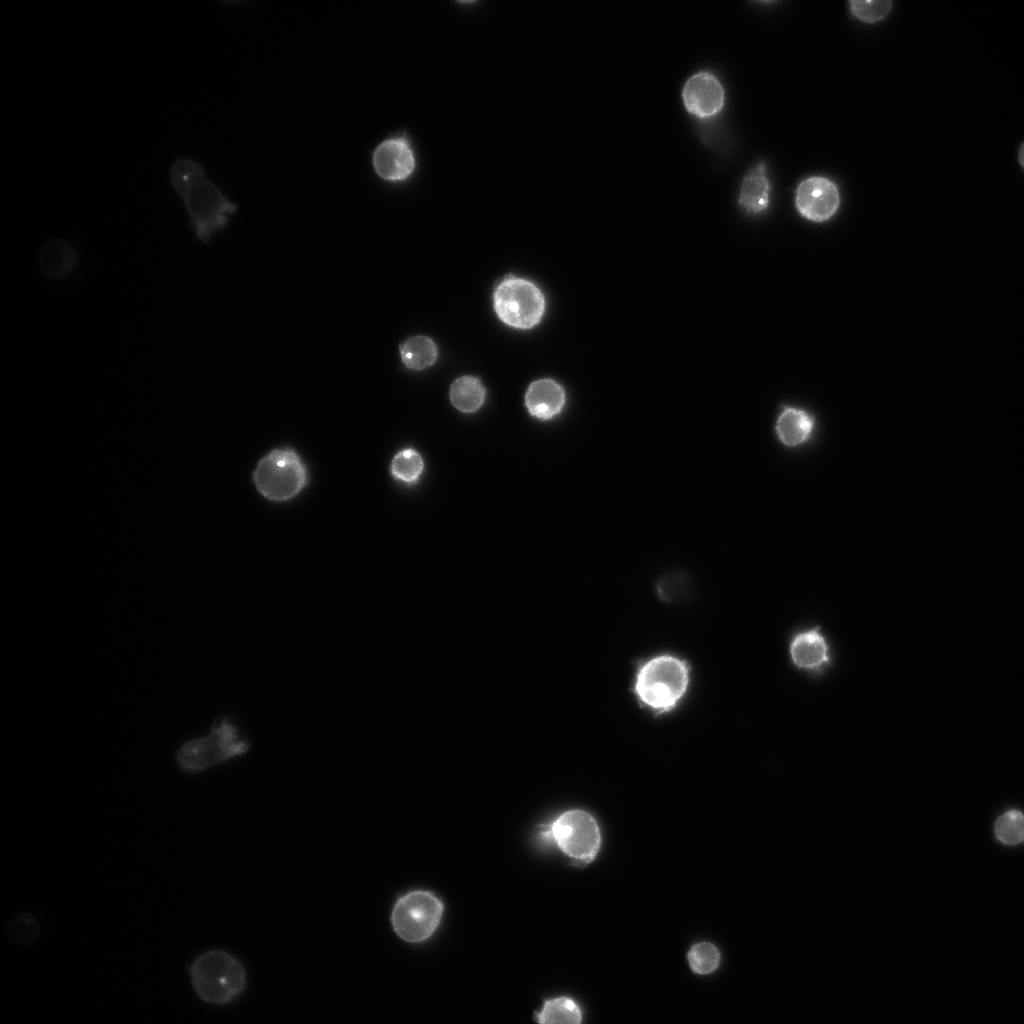

Supplement: Supplementary file 7 — Figure EV1 Source Data [file 44319_2025_533_MOESM7_ESM.zip › Figure EV1/Figure EV1A/Original image Pericentrin cell1,2,3.tif]

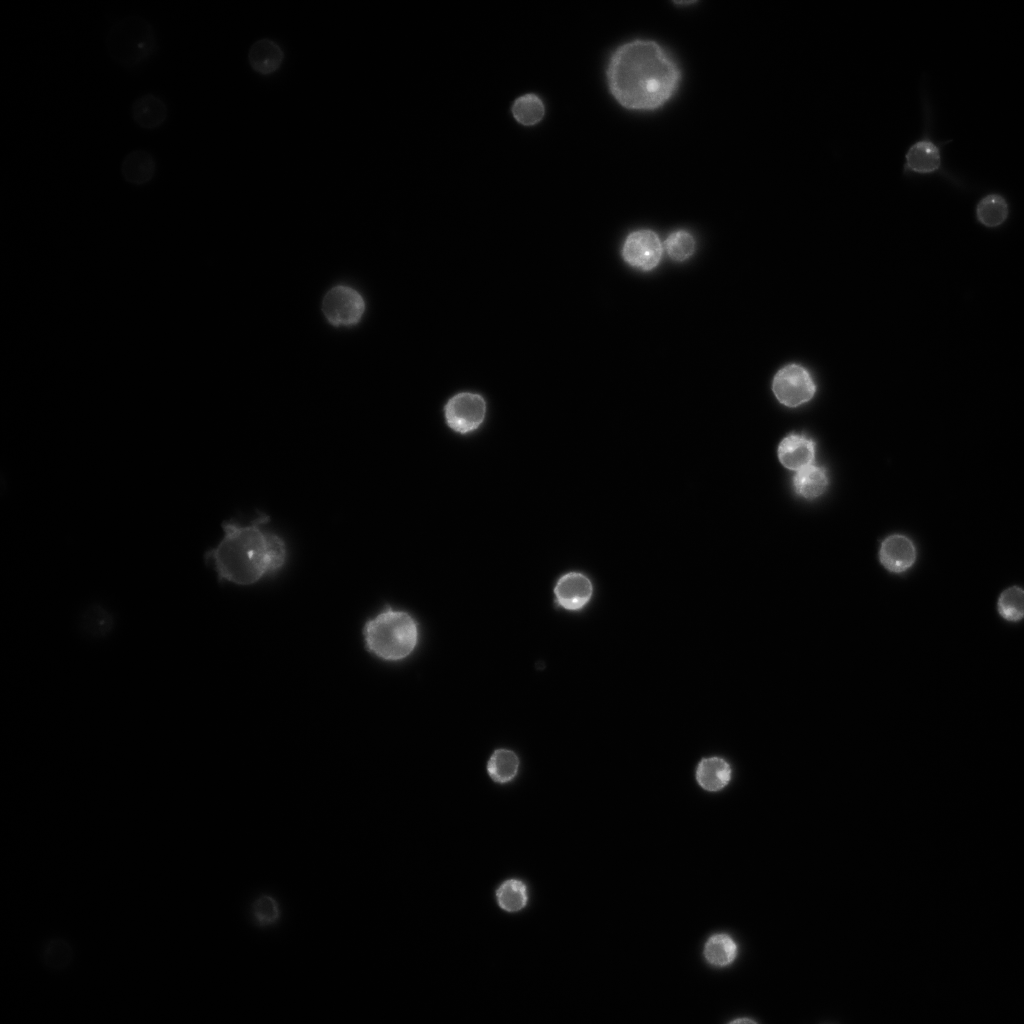

Supplement: Supplementary file 7 — Figure EV1 Source Data [file 44319_2025_533_MOESM7_ESM.zip › Figure EV1/Figure EV1A/Original image Pericentrin cell4.tif]

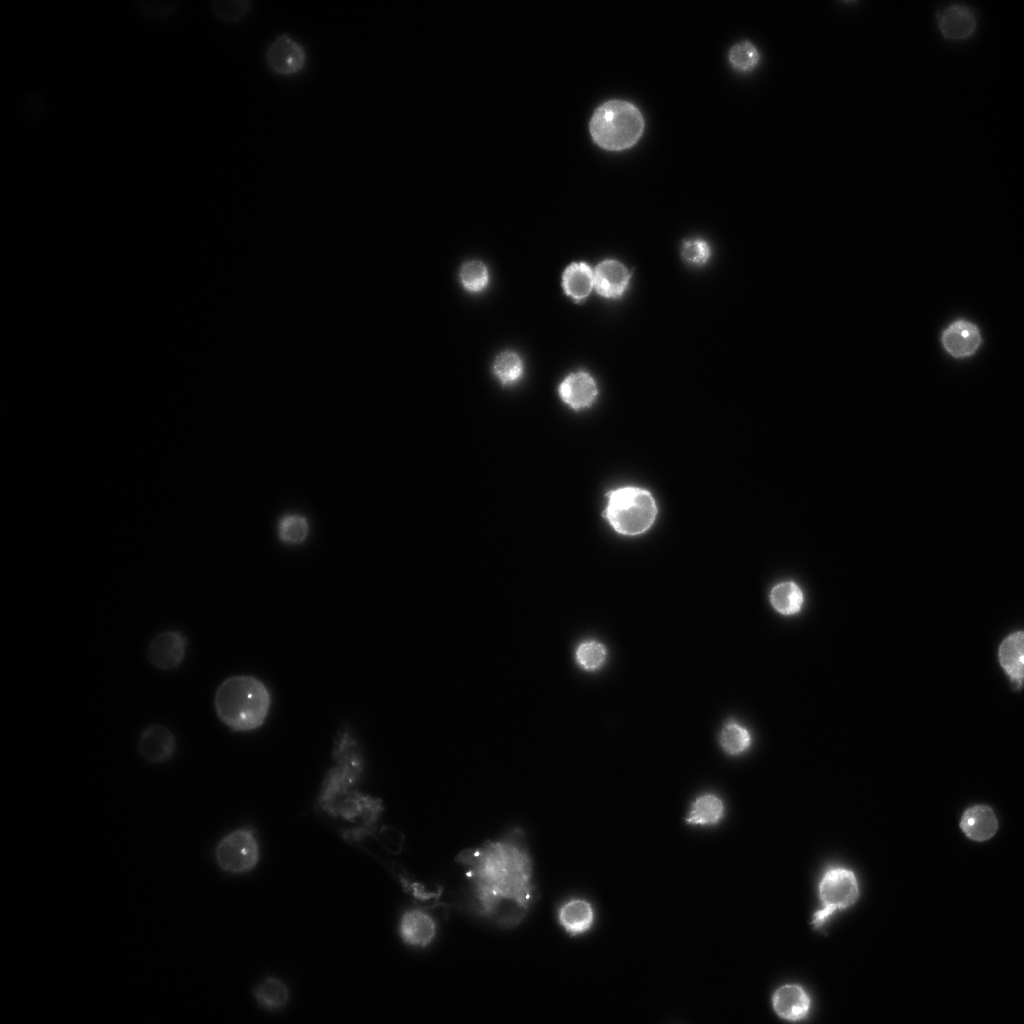

Supplement: Supplementary file 7 — Figure EV1 Source Data [file 44319_2025_533_MOESM7_ESM.zip › Figure EV1/Figure EV1A/Original image Pericentrin cell5.tif]

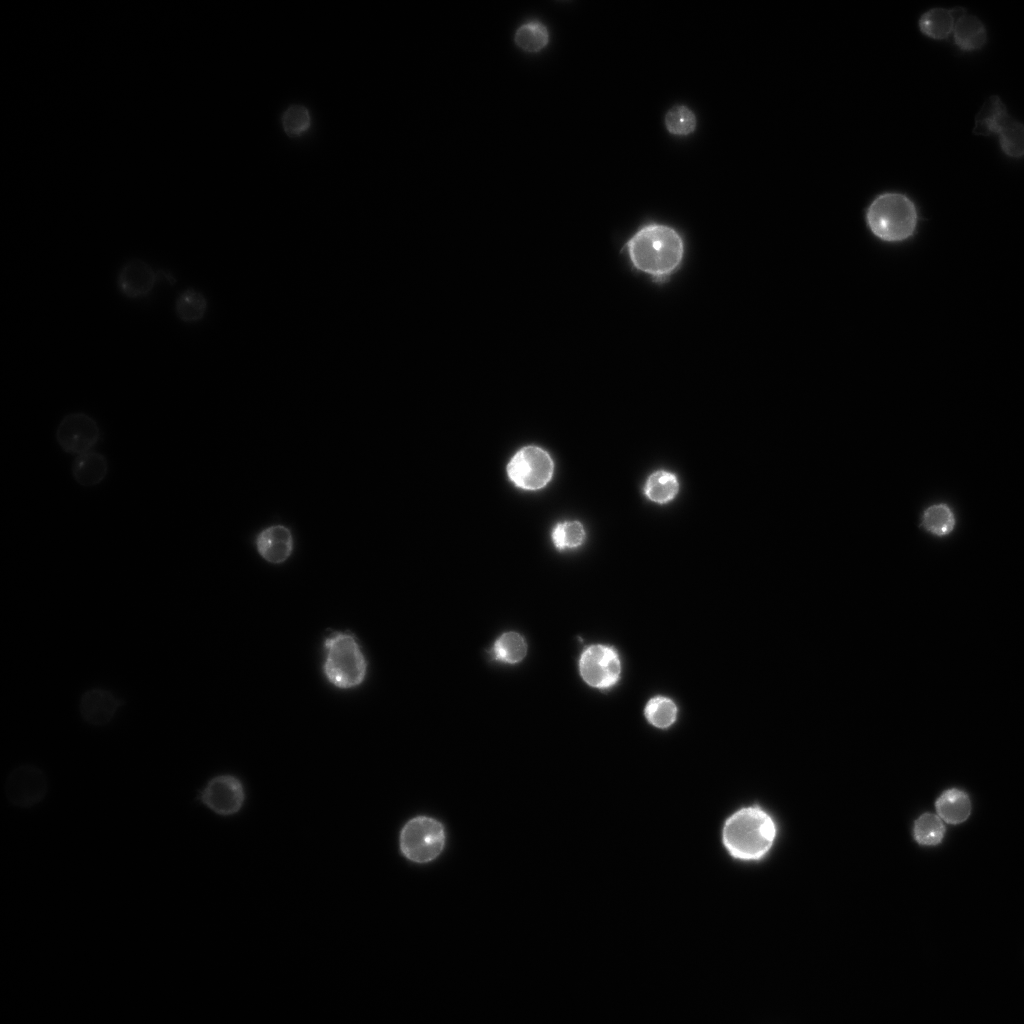

Supplement: Supplementary file 7 — Figure EV1 Source Data [file 44319_2025_533_MOESM7_ESM.zip › Figure EV1/Figure EV1A/Original image Pericentrin cell6.tif]

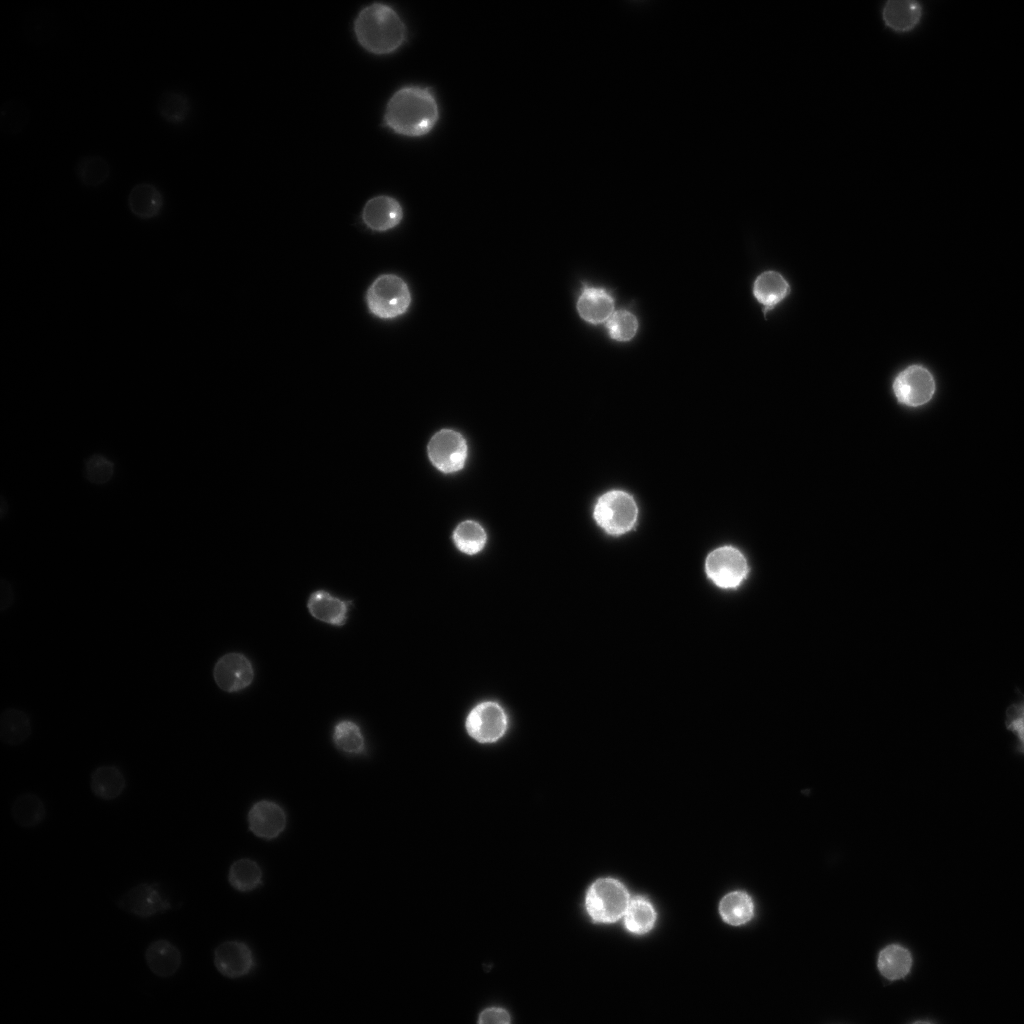

Supplement: Supplementary file 7 — Figure EV1 Source Data [file 44319_2025_533_MOESM7_ESM.zip › Figure EV1/Figure EV1A/Original image Pericentrin cell7,8.tif]

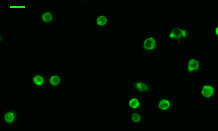

Supplement: Supplementary file 8 — Figure EV2 Source Data [file 44319_2025_533_MOESM8_ESM.zip › Figure EV2/Figure EV2A/Control example of morphology.tif]

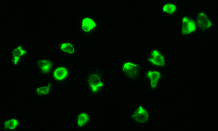

Supplement: Supplementary file 8 — Figure EV2 Source Data [file 44319_2025_533_MOESM8_ESM.zip › Figure EV2/Figure EV2A/CXCL12 example of morphology.tif]

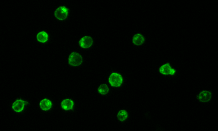

Supplement: Supplementary file 8 — Figure EV2 Source Data [file 44319_2025_533_MOESM8_ESM.zip › Figure EV2/Figure EV2A/H89 CXCL12 example of morphology.tif]

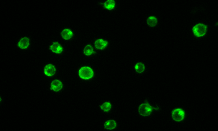

Supplement: Supplementary file 8 — Figure EV2 Source Data [file 44319_2025_533_MOESM8_ESM.zip › Figure EV2/Figure EV2A/H89 example of morphology.tif]

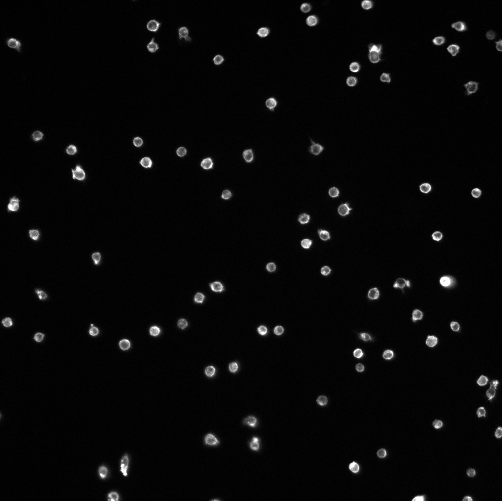

Supplement: Supplementary file 8 — Figure EV2 Source Data [file 44319_2025_533_MOESM8_ESM.zip › Figure EV2/Figure EV2A/Original image control.tif]

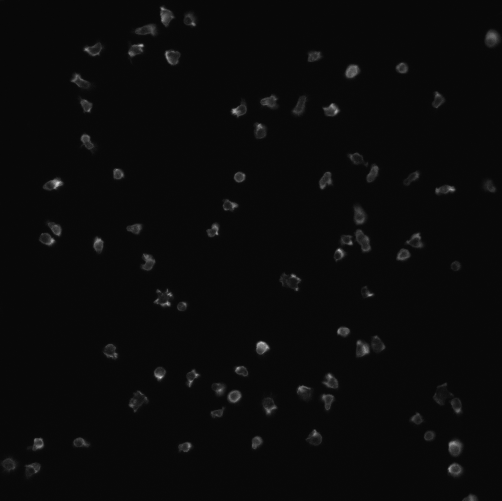

Supplement: Supplementary file 8 — Figure EV2 Source Data [file 44319_2025_533_MOESM8_ESM.zip › Figure EV2/Figure EV2A/Original image CXCL12.tif]

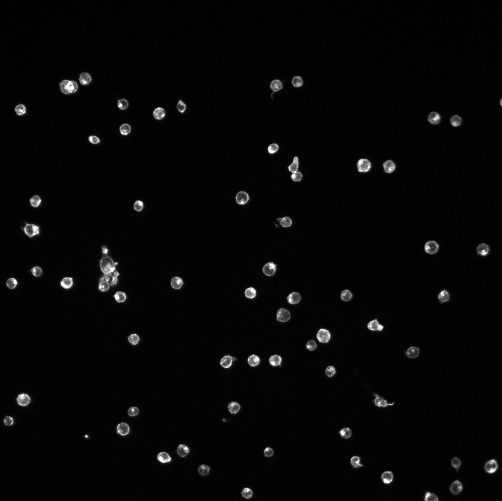

Supplement: Supplementary file 8 — Figure EV2 Source Data [file 44319_2025_533_MOESM8_ESM.zip › Figure EV2/Figure EV2A/Original image H89 CXCL12.tif]

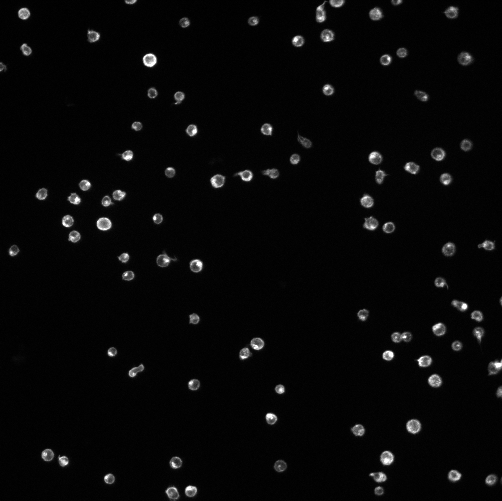

Supplement: Supplementary file 8 — Figure EV2 Source Data [file 44319_2025_533_MOESM8_ESM.zip › Figure EV2/Figure EV2A/Original image H89.tif]

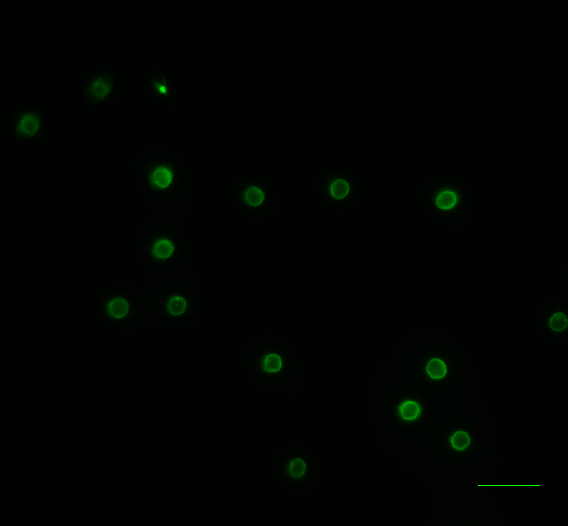

Supplement: Supplementary file 9 — Figure EV3 Source Data [file 44319_2025_533_MOESM9_ESM.zip › Figure EV3/Figure EV3A/Control PBT morphology.tif]

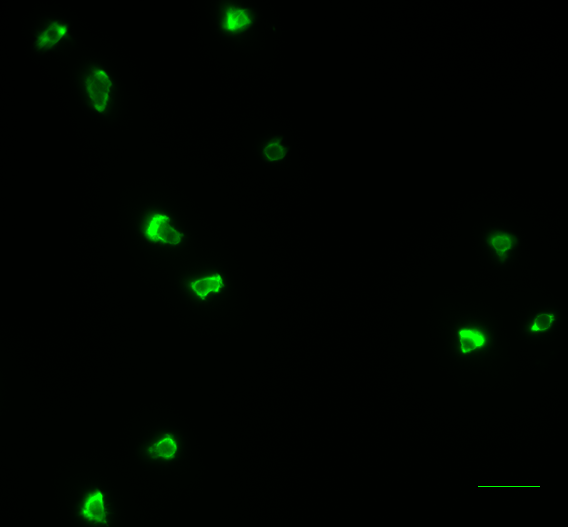

Supplement: Supplementary file 9 — Figure EV3 Source Data [file 44319_2025_533_MOESM9_ESM.zip › Figure EV3/Figure EV3A/CXCL12 PBT morphology.tif]

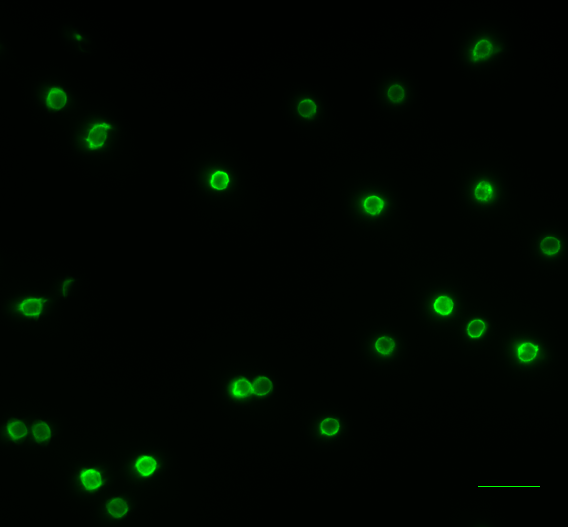

Supplement: Supplementary file 9 — Figure EV3 Source Data [file 44319_2025_533_MOESM9_ESM.zip › Figure EV3/Figure EV3A/H89 CXCL12 PBT morphology.tif]

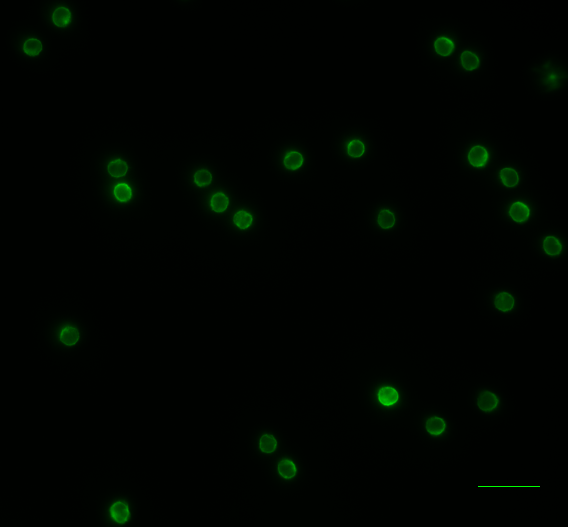

Supplement: Supplementary file 9 — Figure EV3 Source Data [file 44319_2025_533_MOESM9_ESM.zip › Figure EV3/Figure EV3A/H89 PBT morphology.tif]

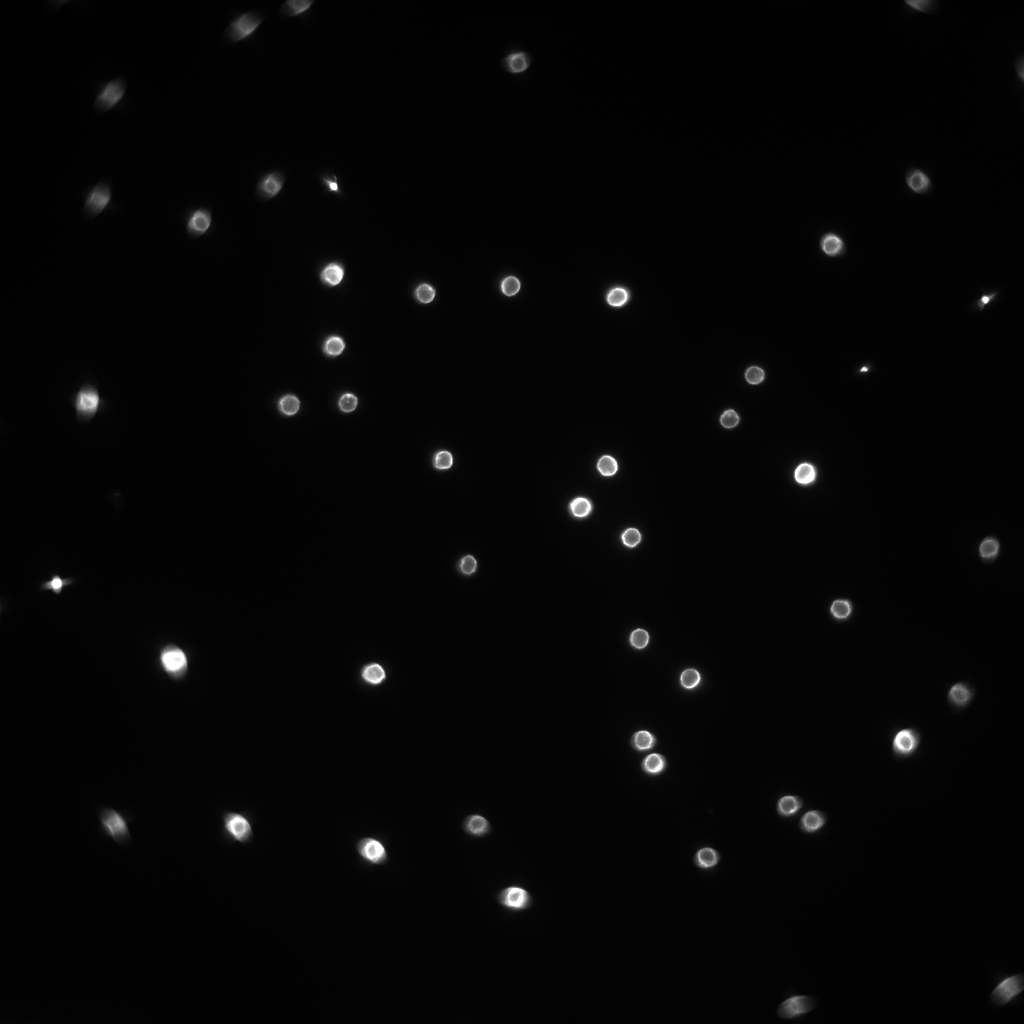

Supplement: Supplementary file 9 — Figure EV3 Source Data [file 44319_2025_533_MOESM9_ESM.zip › Figure EV3/Figure EV3A/Original image control actin.tif]

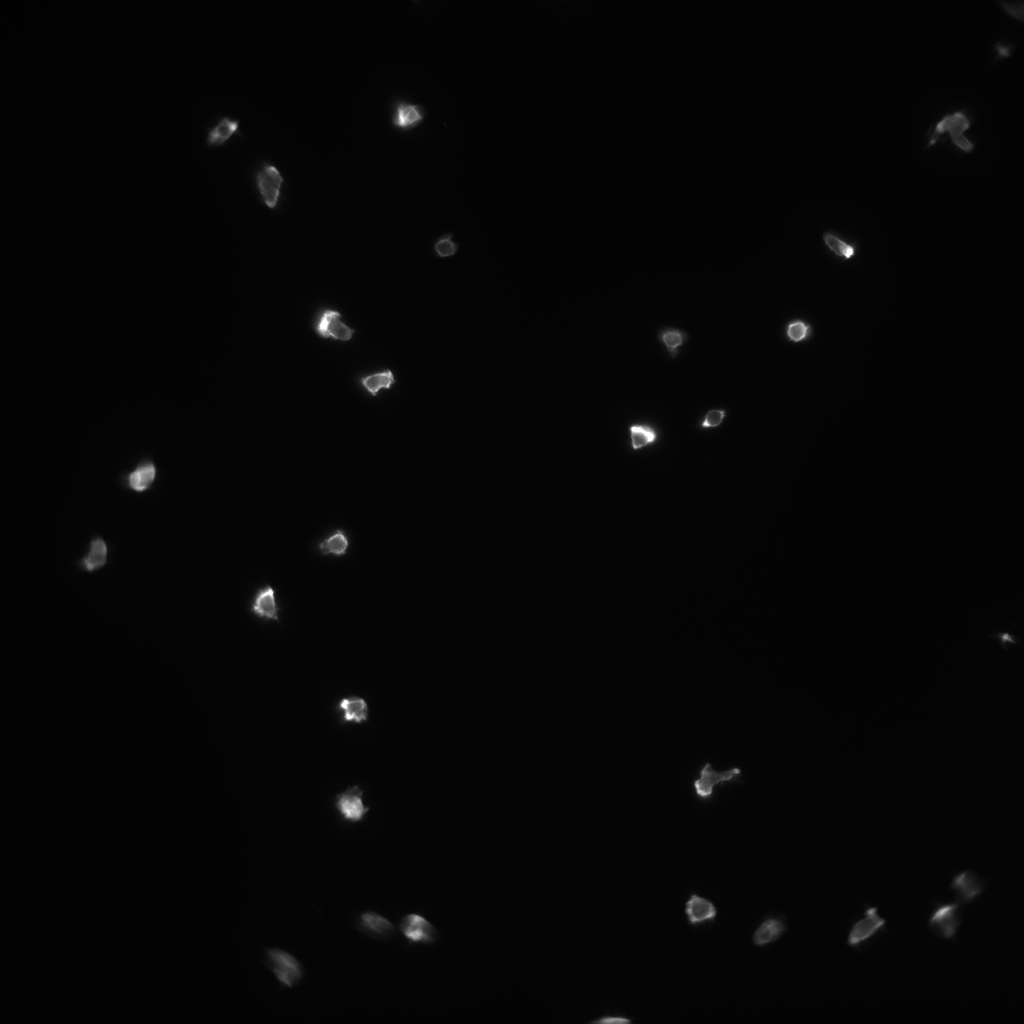

Supplement: Supplementary file 9 — Figure EV3 Source Data [file 44319_2025_533_MOESM9_ESM.zip › Figure EV3/Figure EV3A/Original image CXCL12 actin.tif]

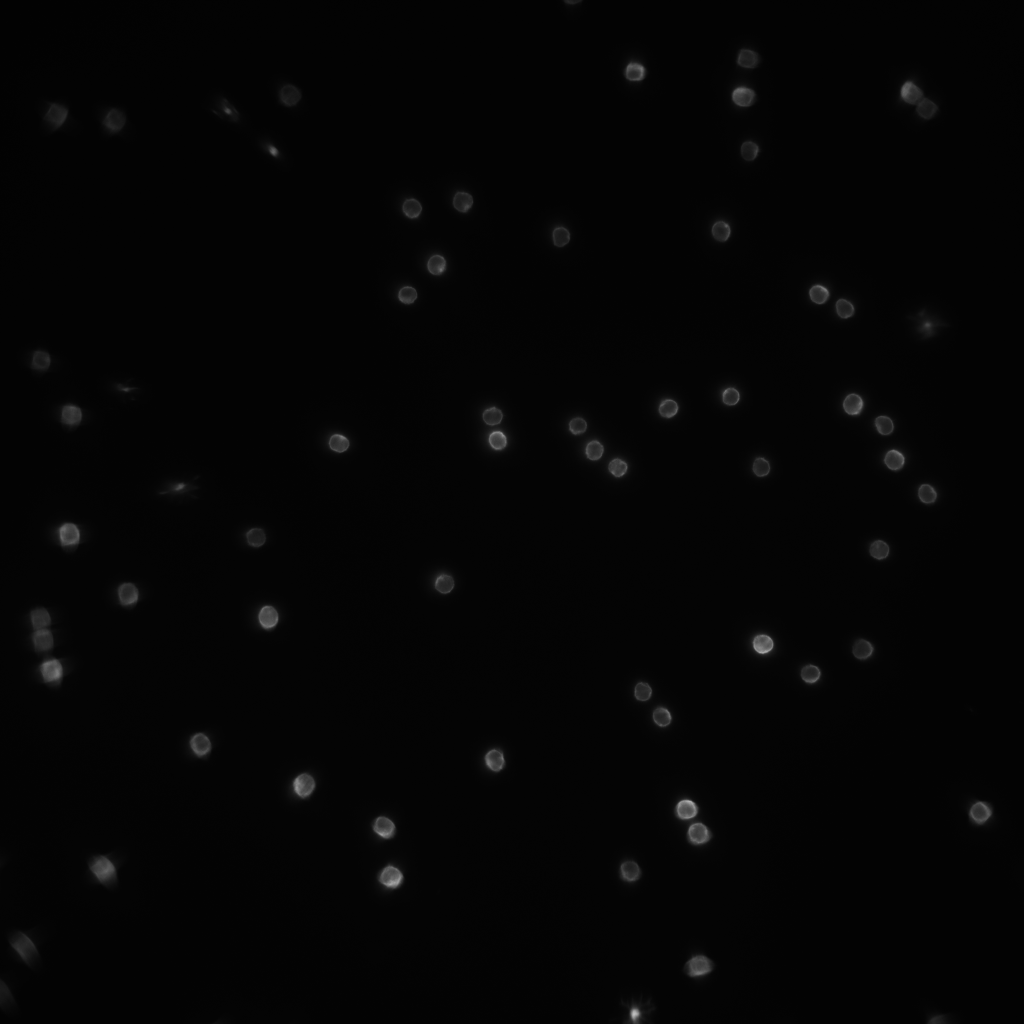

Supplement: Supplementary file 9 — Figure EV3 Source Data [file 44319_2025_533_MOESM9_ESM.zip › Figure EV3/Figure EV3A/Original image H89 actin.tif]

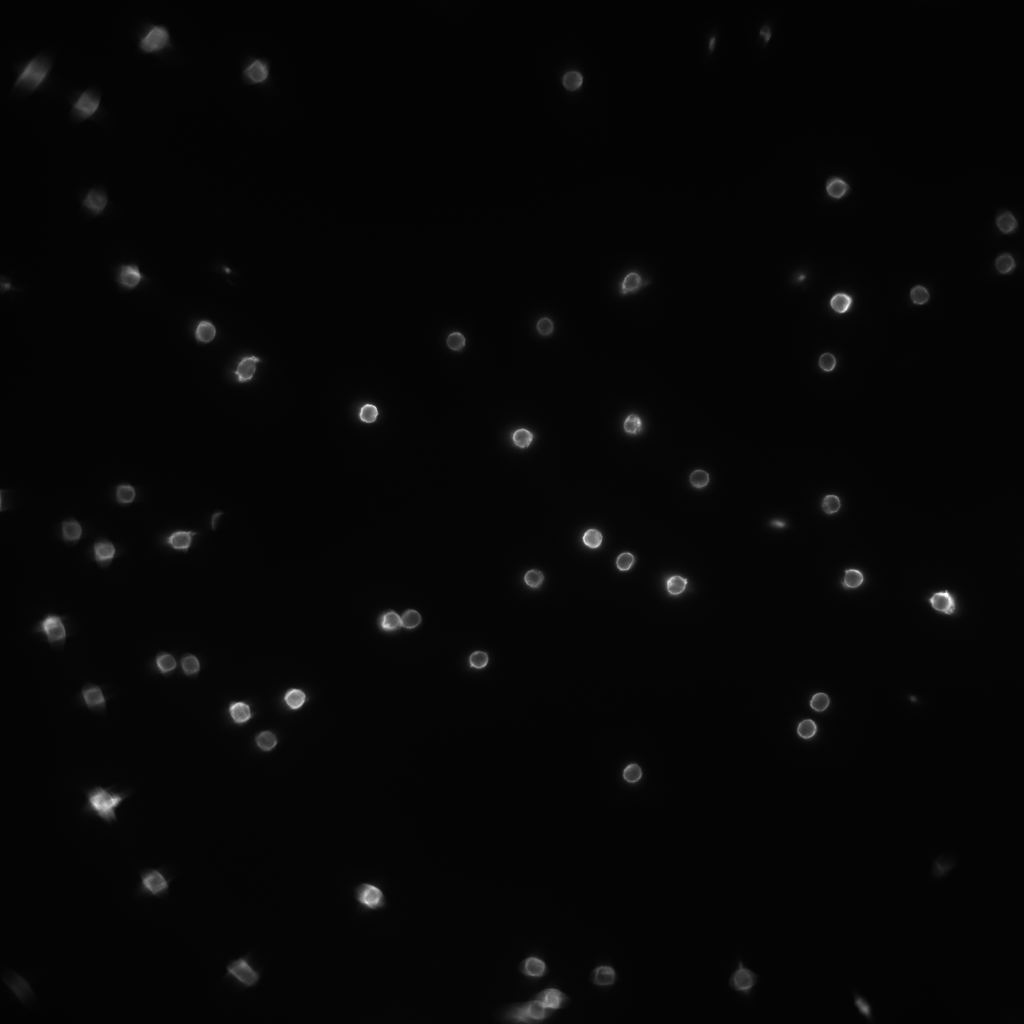

Supplement: Supplementary file 9 — Figure EV3 Source Data [file 44319_2025_533_MOESM9_ESM.zip › Figure EV3/Figure EV3A/Original image H89 CXCL12 actin.tif]

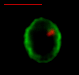

Supplement: Supplementary file 9 — Figure EV3 Source Data [file 44319_2025_533_MOESM9_ESM.zip › Figure EV3/Figure EV3C/Control PBT Actin and Pericentrin.tif]

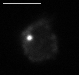

Supplement: Supplementary file 9 — Figure EV3 Source Data [file 44319_2025_533_MOESM9_ESM.zip › Figure EV3/Figure EV3C/CXCL12 PBT Actin and Pericentrin.tif]

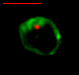

Supplement: Supplementary file 9 — Figure EV3 Source Data [file 44319_2025_533_MOESM9_ESM.zip › Figure EV3/Figure EV3C/H89 CXCL12 PBT Actin and Pericentrin .tif]

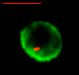

Supplement: Supplementary file 9 — Figure EV3 Source Data [file 44319_2025_533_MOESM9_ESM.zip › Figure EV3/Figure EV3C/H89 PBT Actin and Pericentrin.tif]

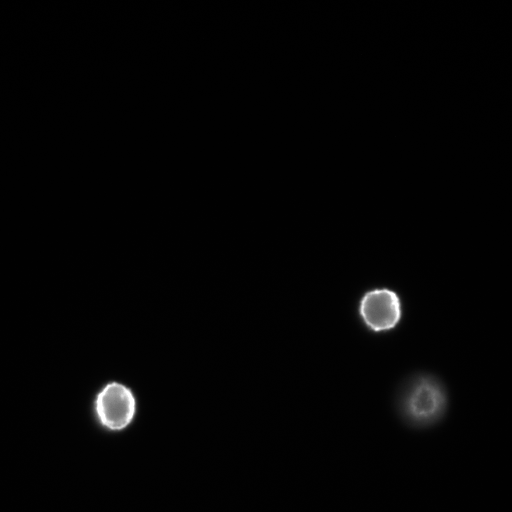

Supplement: Supplementary file 9 — Figure EV3 Source Data [file 44319_2025_533_MOESM9_ESM.zip › Figure EV3/Figure EV3C/Original Image Control Actin.tif]

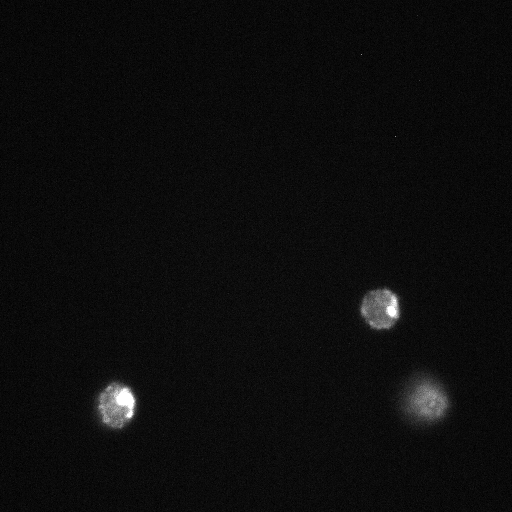

Supplement: Supplementary file 9 — Figure EV3 Source Data [file 44319_2025_533_MOESM9_ESM.zip › Figure EV3/Figure EV3C/Original Image Control Pericentrin.tif]

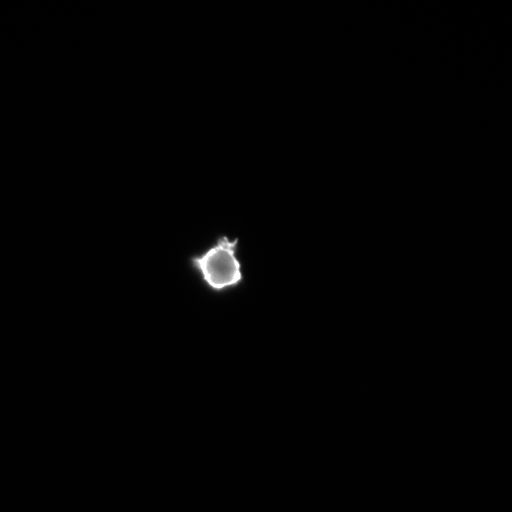

Supplement: Supplementary file 9 — Figure EV3 Source Data [file 44319_2025_533_MOESM9_ESM.zip › Figure EV3/Figure EV3C/Original Image CXCL12 Actin.tif]

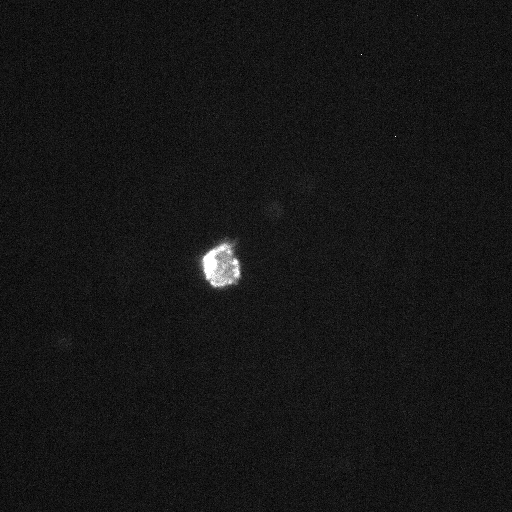

Supplement: Supplementary file 9 — Figure EV3 Source Data [file 44319_2025_533_MOESM9_ESM.zip › Figure EV3/Figure EV3C/Original Image CXL12 Pericentrin.tif]

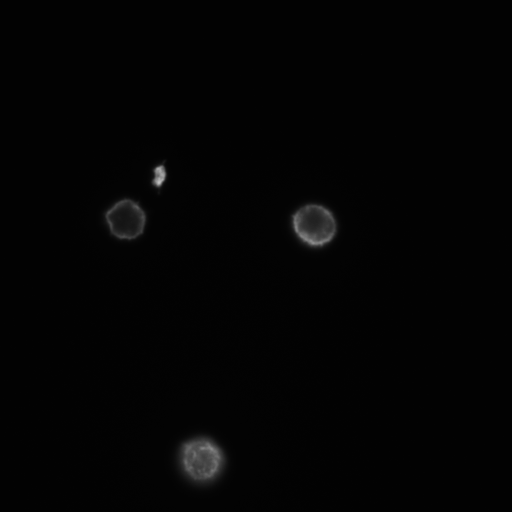

Supplement: Supplementary file 9 — Figure EV3 Source Data [file 44319_2025_533_MOESM9_ESM.zip › Figure EV3/Figure EV3C/Original Image H89 Actin.tif]

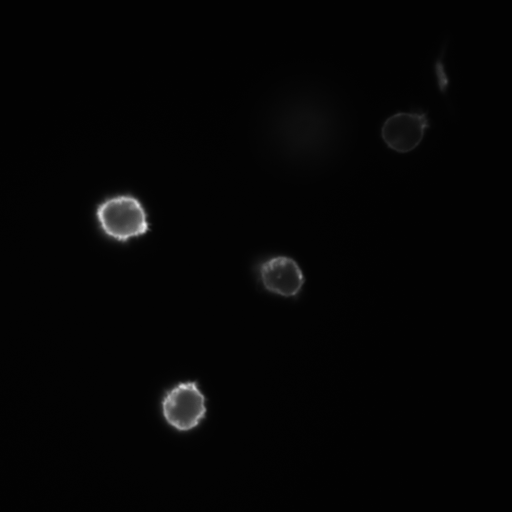

Supplement: Supplementary file 9 — Figure EV3 Source Data [file 44319_2025_533_MOESM9_ESM.zip › Figure EV3/Figure EV3C/Original Image H89 CXCL12 Actin.tif]

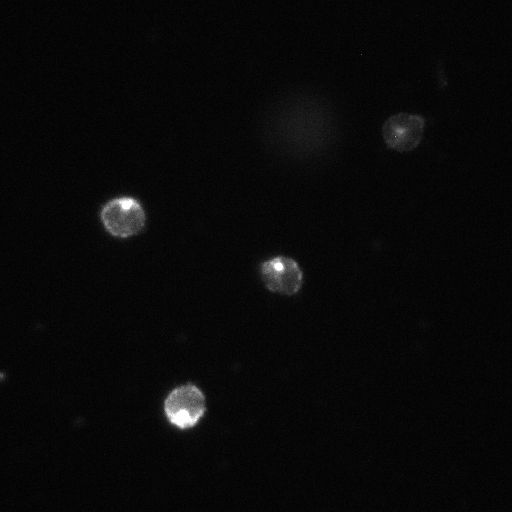

Supplement: Supplementary file 9 — Figure EV3 Source Data [file 44319_2025_533_MOESM9_ESM.zip › Figure EV3/Figure EV3C/Original Image H89 CXCL12 Pericentrin.tif]

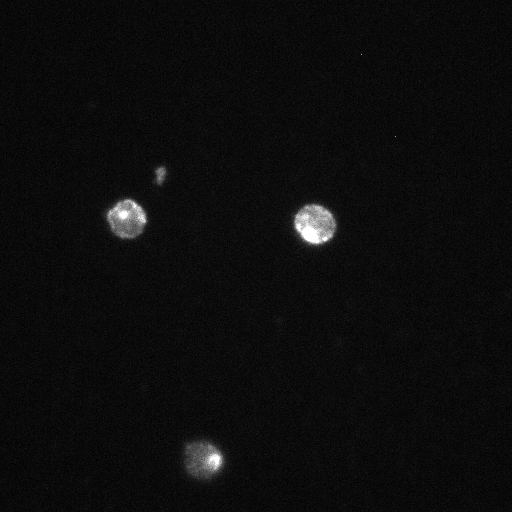

Supplement: Supplementary file 9 — Figure EV3 Source Data [file 44319_2025_533_MOESM9_ESM.zip › Figure EV3/Figure EV3C/Original Image H89 Pericentrin.tif]

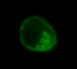

Supplement: Supplementary file 10 — Figure EV4 Source Data [file 44319_2025_533_MOESM10_ESM.zip › Figure EV4/Figure EV4C/Control Actin.tif]
