# Supplementary figures and images for: Centrosomal actin pool levels regulated by localized PKA set the threshold for T cell polarization (part 2 of 2)
Source: EMBO Rep. 2025 Aug 26;26(18):4436–55. doi: 10.1038/s44319-025-00533-2 (PMC12457651; doi:10.1038/s44319-025-00533-2)

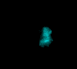

Supplement: Supplementary file 10 — Figure EV4 Source Data [file 44319_2025_533_MOESM10_ESM.zip › Figure EV4/Figure EV4C/Control Arp2.tif]

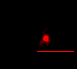

Supplement: Supplementary file 10 — Figure EV4 Source Data [file 44319_2025_533_MOESM10_ESM.zip › Figure EV4/Figure EV4C/Control Pericentrin.tif]

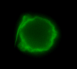

Supplement: Supplementary file 10 — Figure EV4 Source Data [file 44319_2025_533_MOESM10_ESM.zip › Figure EV4/Figure EV4C/CXCL12 Actin.tif]

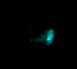

Supplement: Supplementary file 10 — Figure EV4 Source Data [file 44319_2025_533_MOESM10_ESM.zip › Figure EV4/Figure EV4C/CXCL12 Arp2.tif]

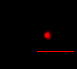

Supplement: Supplementary file 10 — Figure EV4 Source Data [file 44319_2025_533_MOESM10_ESM.zip › Figure EV4/Figure EV4C/CXCL12 Pericentrin.tif]

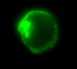

Supplement: Supplementary file 10 — Figure EV4 Source Data [file 44319_2025_533_MOESM10_ESM.zip › Figure EV4/Figure EV4C/H89 Actin.tif]

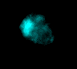

Supplement: Supplementary file 10 — Figure EV4 Source Data [file 44319_2025_533_MOESM10_ESM.zip › Figure EV4/Figure EV4C/H89 Arp2.tif]

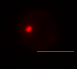

Supplement: Supplementary file 10 — Figure EV4 Source Data [file 44319_2025_533_MOESM10_ESM.zip › Figure EV4/Figure EV4C/H89 Pericentrin.tif]

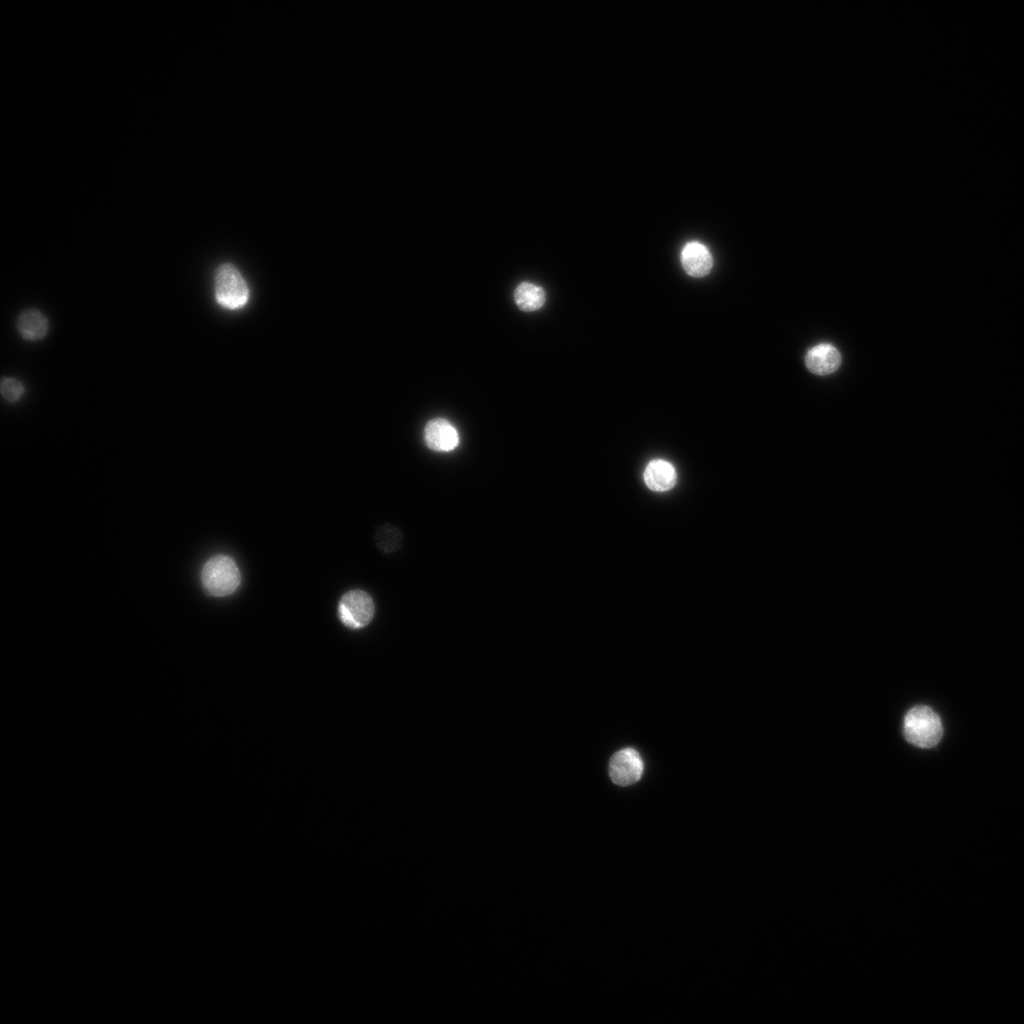

Supplement: Supplementary file 10 — Figure EV4 Source Data [file 44319_2025_533_MOESM10_ESM.zip › Figure EV4/Figure EV4C/Original image Control Arp2.tif]

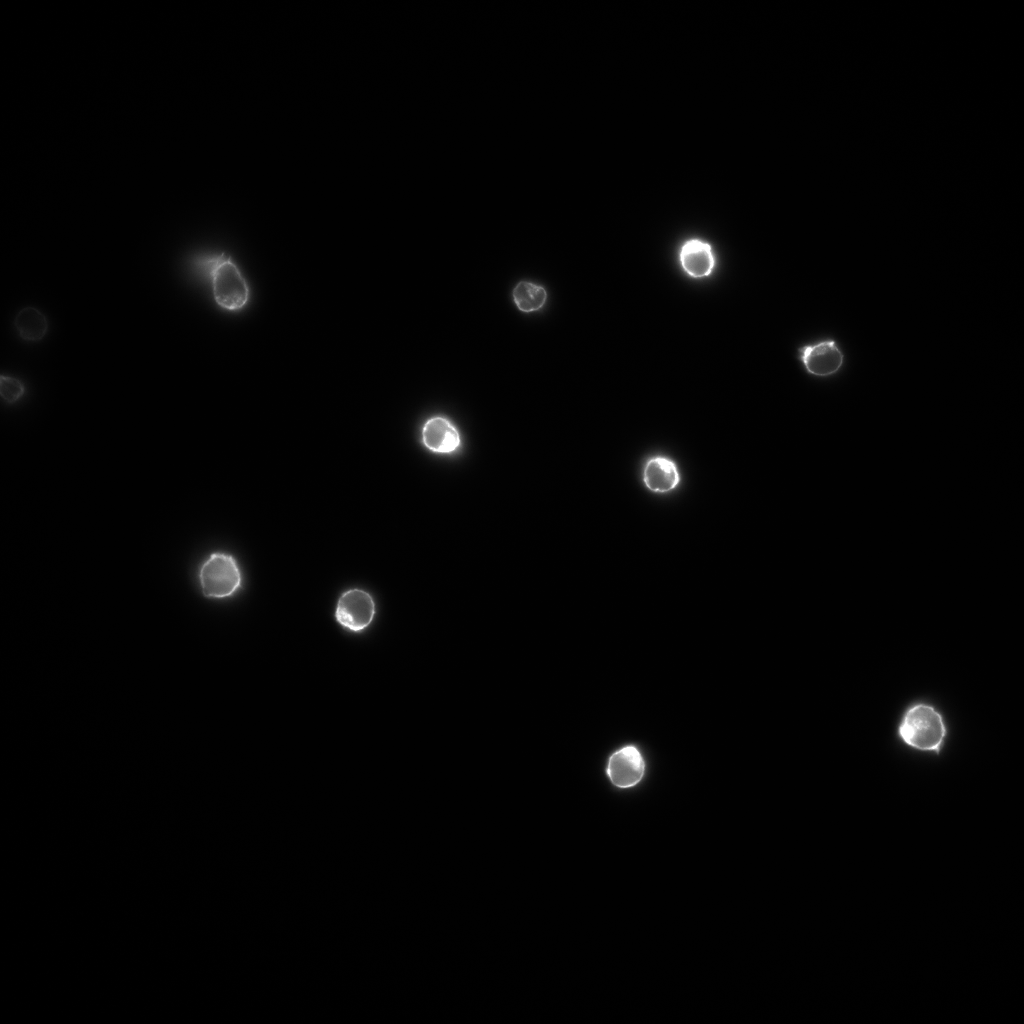

Supplement: Supplementary file 10 — Figure EV4 Source Data [file 44319_2025_533_MOESM10_ESM.zip › Figure EV4/Figure EV4C/Original image Control Actin.tif]

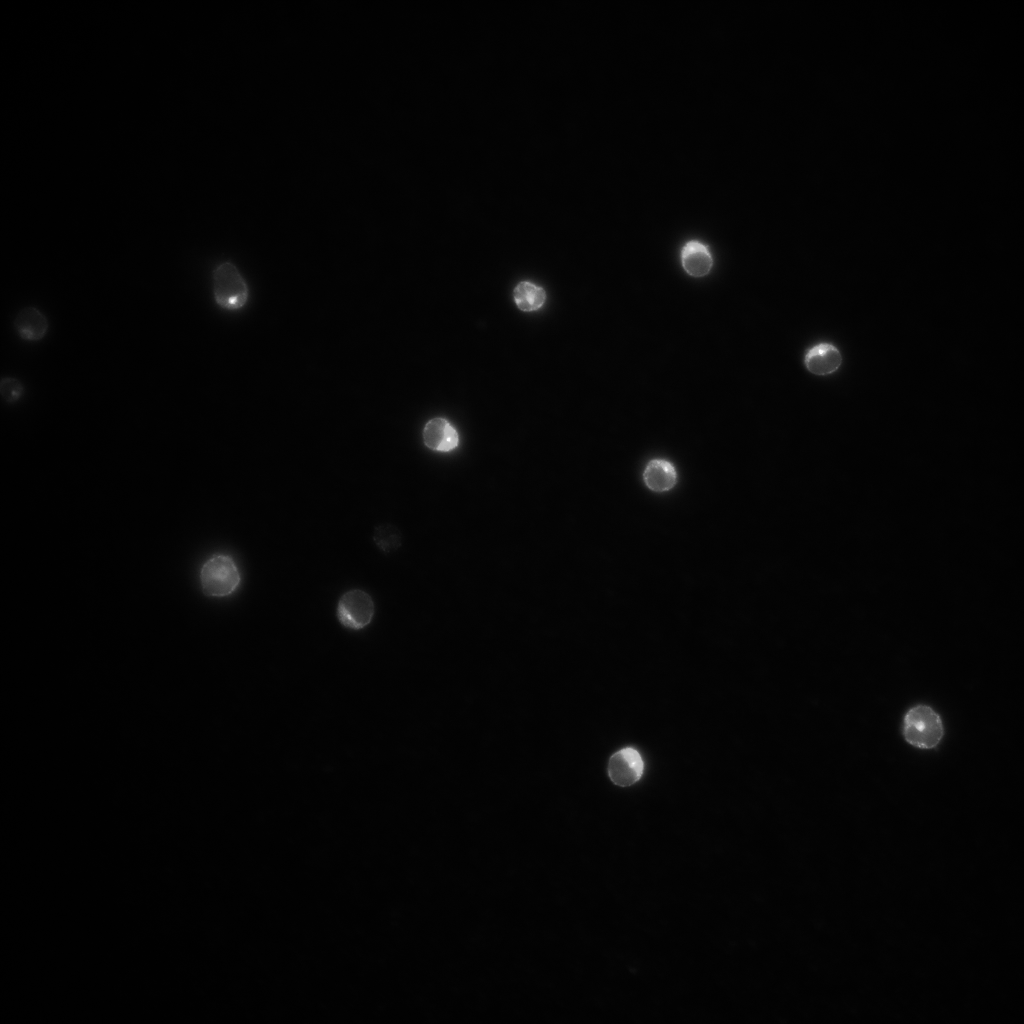

Supplement: Supplementary file 10 — Figure EV4 Source Data [file 44319_2025_533_MOESM10_ESM.zip › Figure EV4/Figure EV4C/Original image Control Pericentrin.tif]

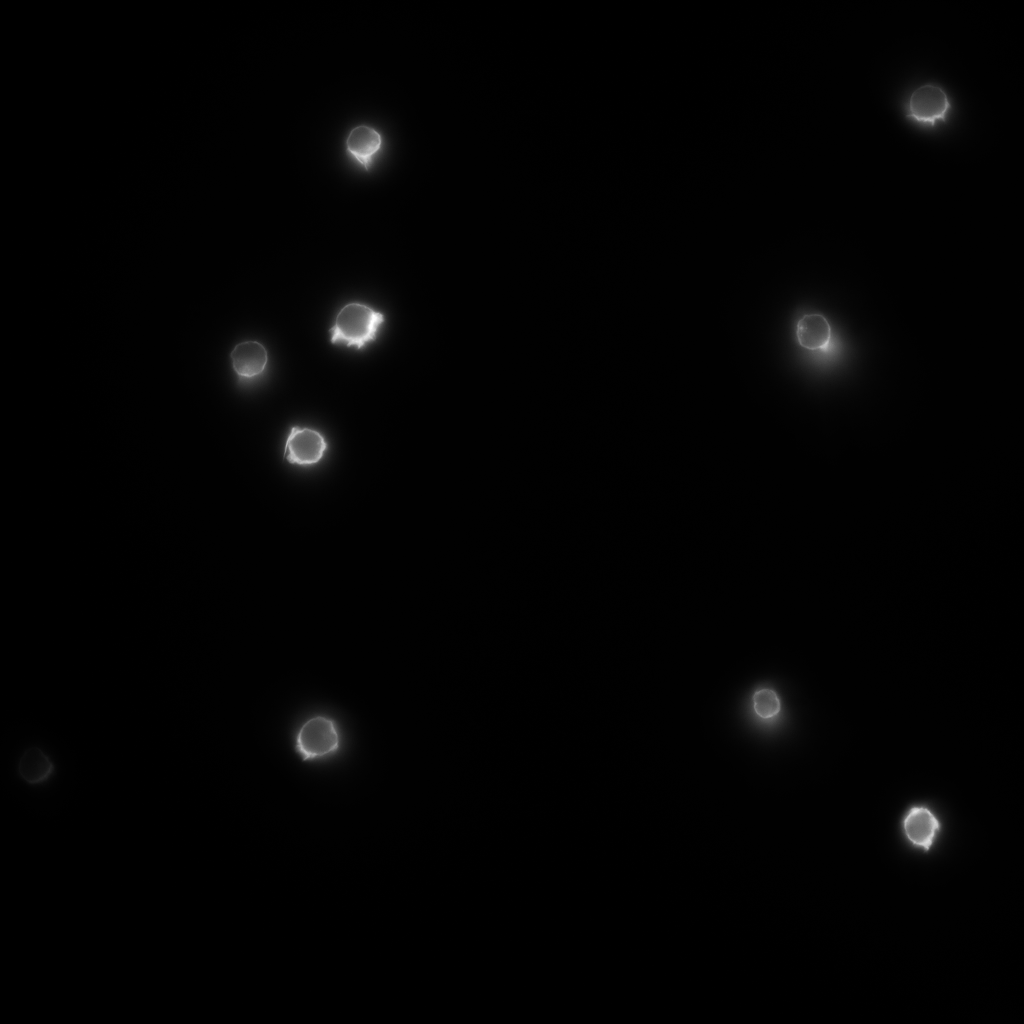

Supplement: Supplementary file 10 — Figure EV4 Source Data [file 44319_2025_533_MOESM10_ESM.zip › Figure EV4/Figure EV4C/Original image CXCL12 Actin.tif]

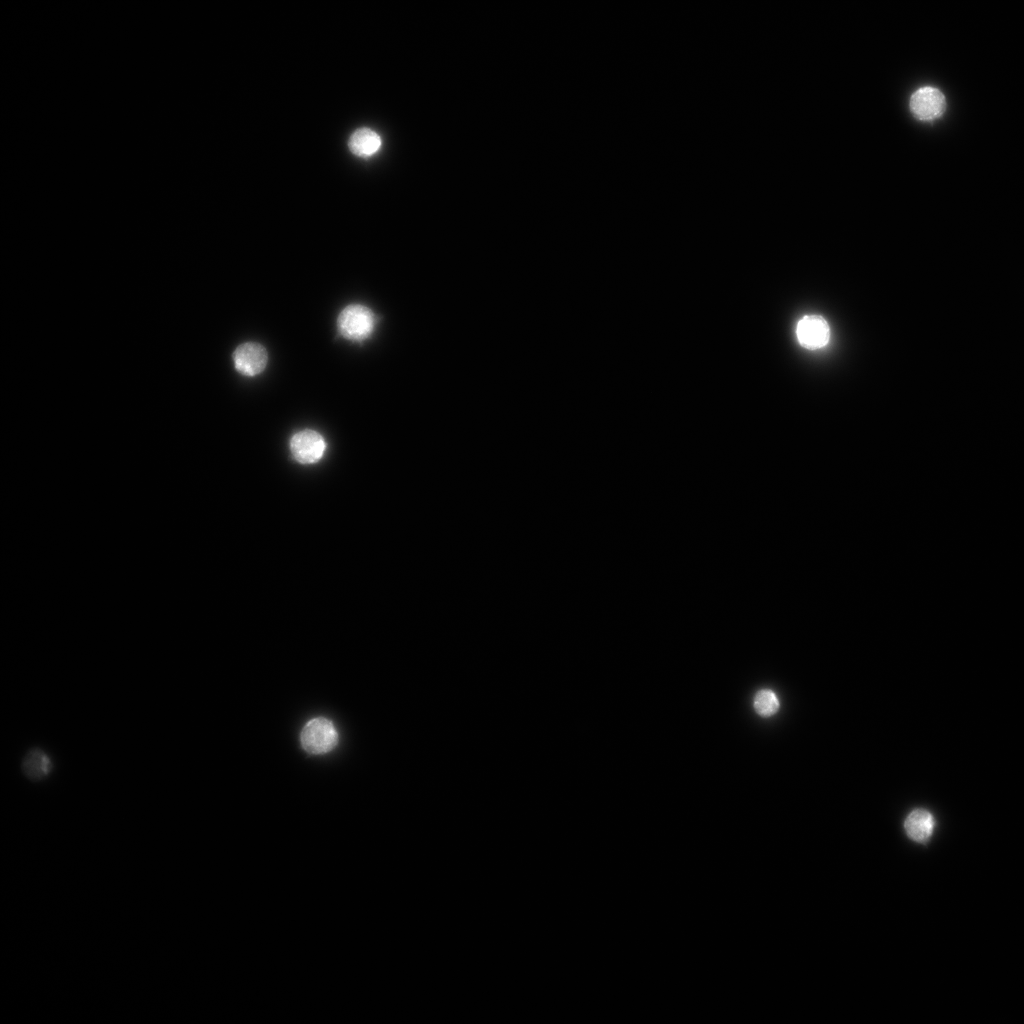

Supplement: Supplementary file 10 — Figure EV4 Source Data [file 44319_2025_533_MOESM10_ESM.zip › Figure EV4/Figure EV4C/Original image CXCL12 Arp2.tif]

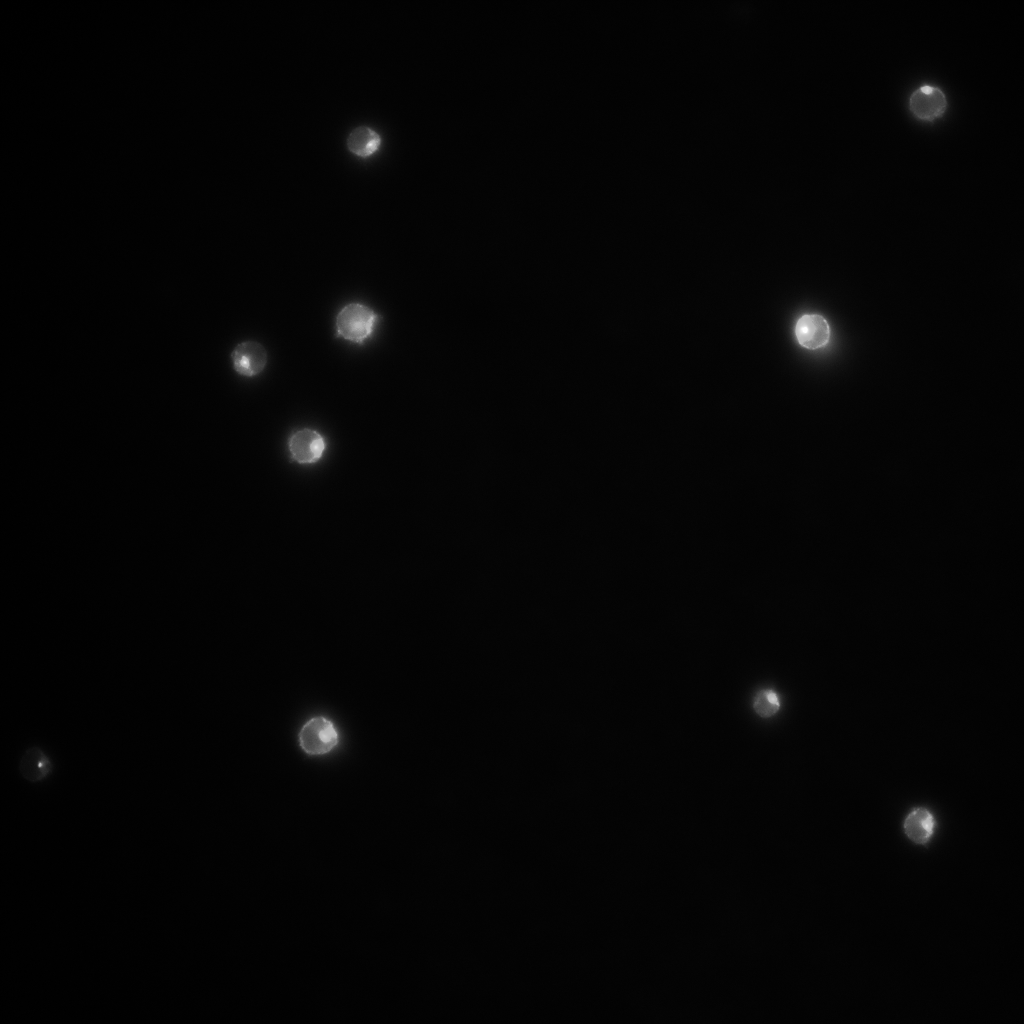

Supplement: Supplementary file 10 — Figure EV4 Source Data [file 44319_2025_533_MOESM10_ESM.zip › Figure EV4/Figure EV4C/Original image CXCL12 Pericentrin.tif]

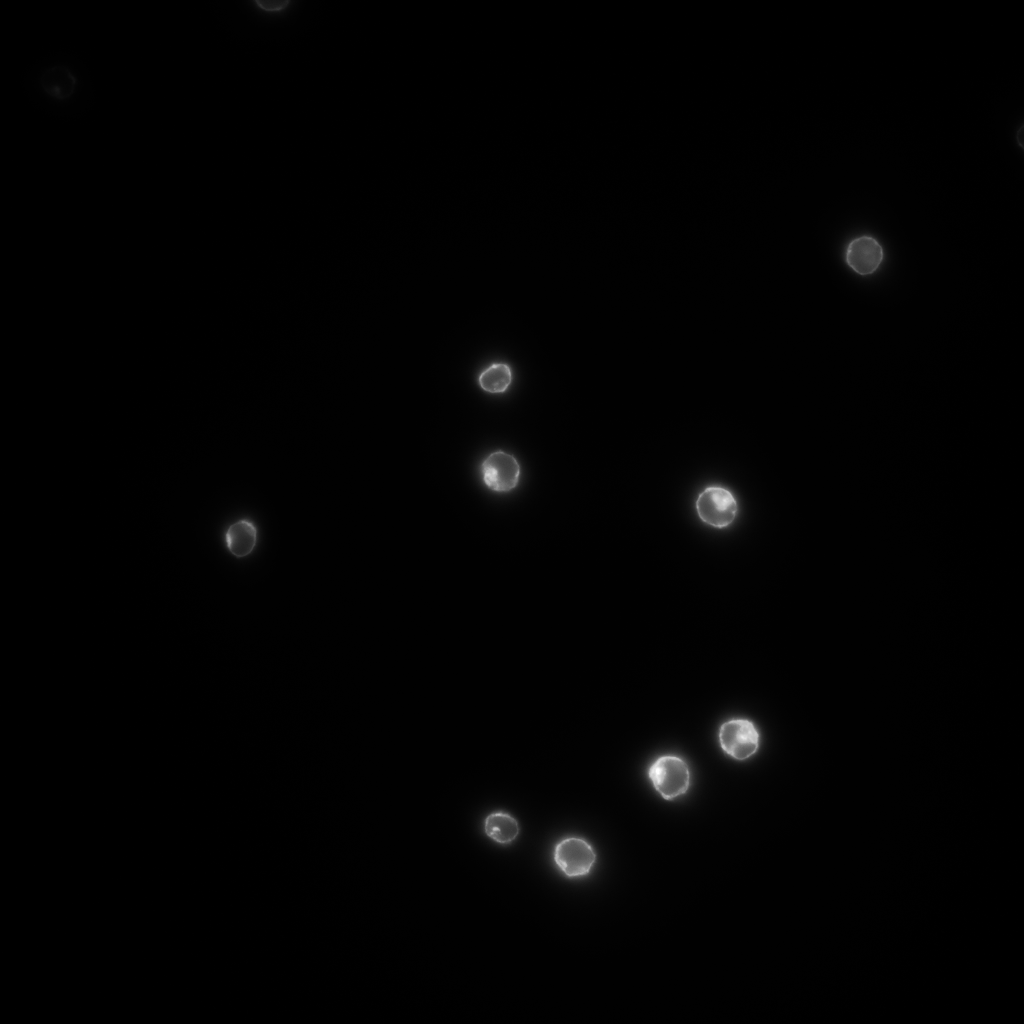

Supplement: Supplementary file 10 — Figure EV4 Source Data [file 44319_2025_533_MOESM10_ESM.zip › Figure EV4/Figure EV4C/Original image H89 Actin.tif]

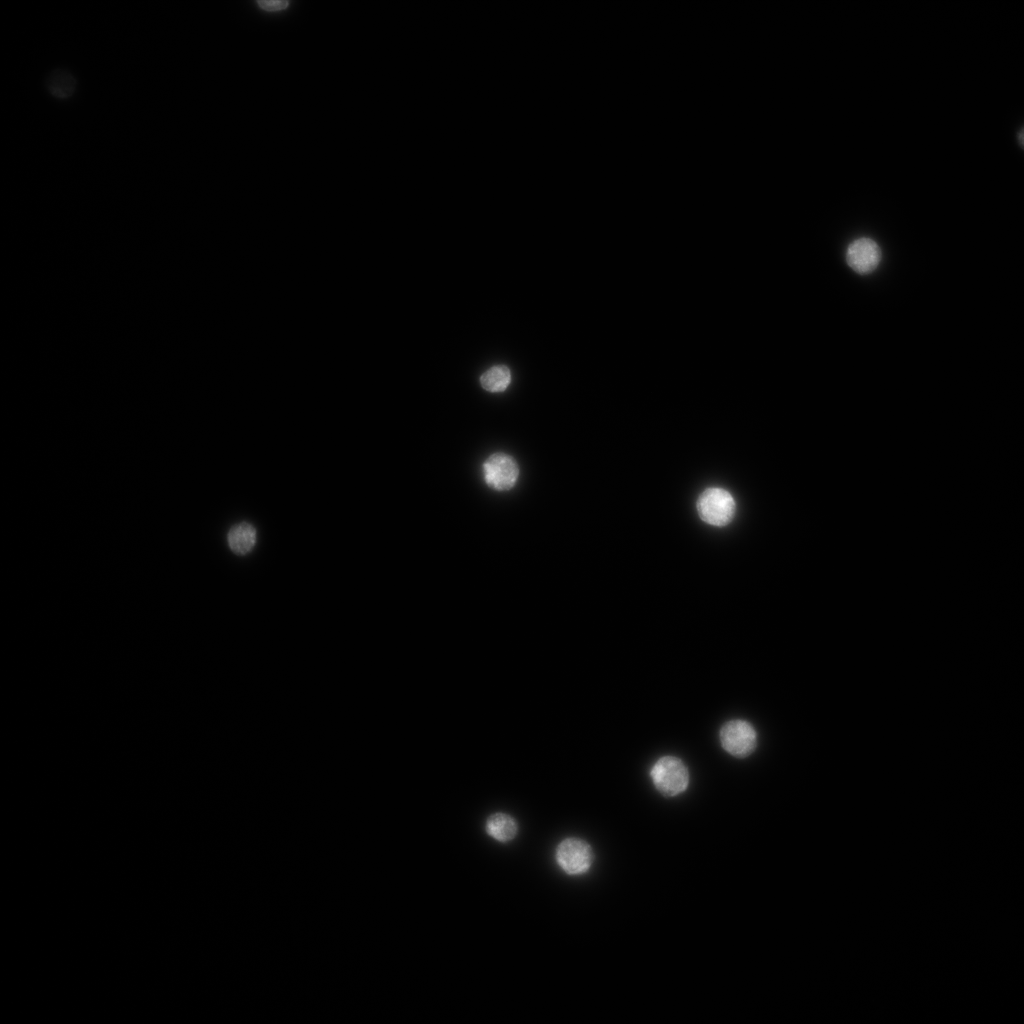

Supplement: Supplementary file 10 — Figure EV4 Source Data [file 44319_2025_533_MOESM10_ESM.zip › Figure EV4/Figure EV4C/Original image H89 Arp2.tif]

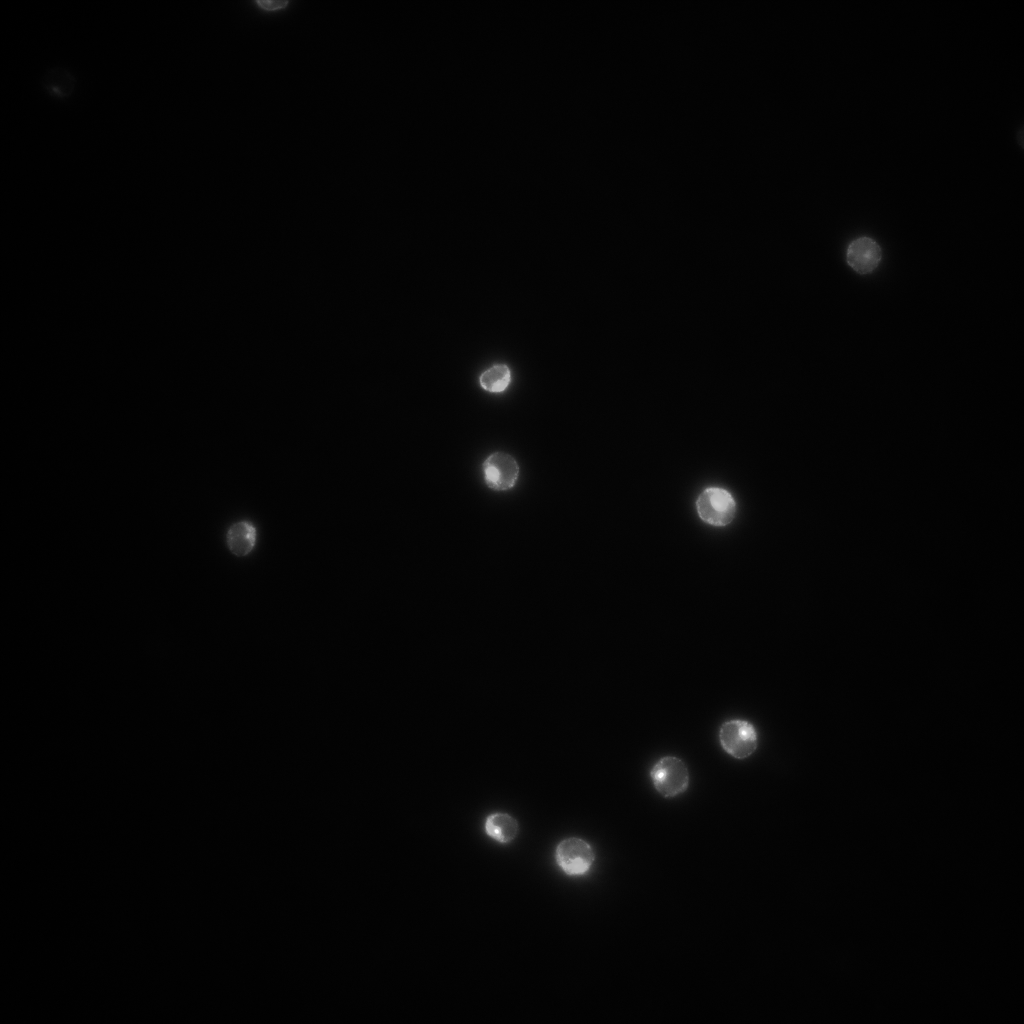

Supplement: Supplementary file 10 — Figure EV4 Source Data [file 44319_2025_533_MOESM10_ESM.zip › Figure EV4/Figure EV4C/Original image H89 Pericentrin.tif]

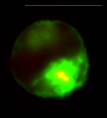

Supplement: Supplementary file 11 — Figure EV5 Source Data [file 44319_2025_533_MOESM11_ESM.zip › Figure EV5/Figure EV5A/Ht31 Actin Pericentrin.tif]

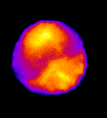

Supplement: Supplementary file 11 — Figure EV5 Source Data [file 44319_2025_533_MOESM11_ESM.zip › Figure EV5/Figure EV5A/Ht31 PKACa.tif]

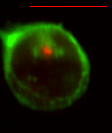

Supplement: Supplementary file 11 — Figure EV5 Source Data [file 44319_2025_533_MOESM11_ESM.zip › Figure EV5/Figure EV5A/Ht31-P Actin Pericentrin.tif]

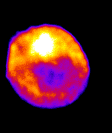

Supplement: Supplementary file 11 — Figure EV5 Source Data [file 44319_2025_533_MOESM11_ESM.zip › Figure EV5/Figure EV5A/Ht31-P PKACa.tif]

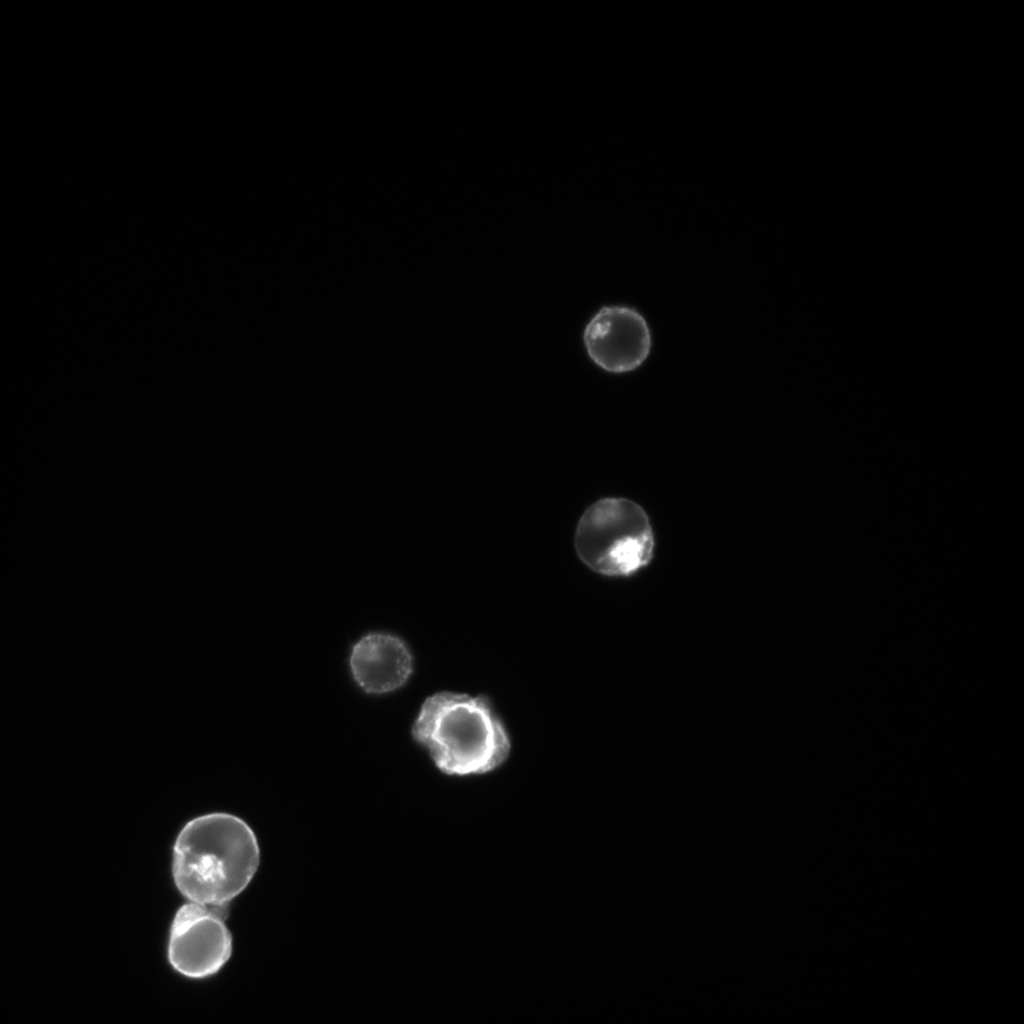

Supplement: Supplementary file 11 — Figure EV5 Source Data [file 44319_2025_533_MOESM11_ESM.zip › Figure EV5/Figure EV5A/Original image Ht31 Actin.tif]

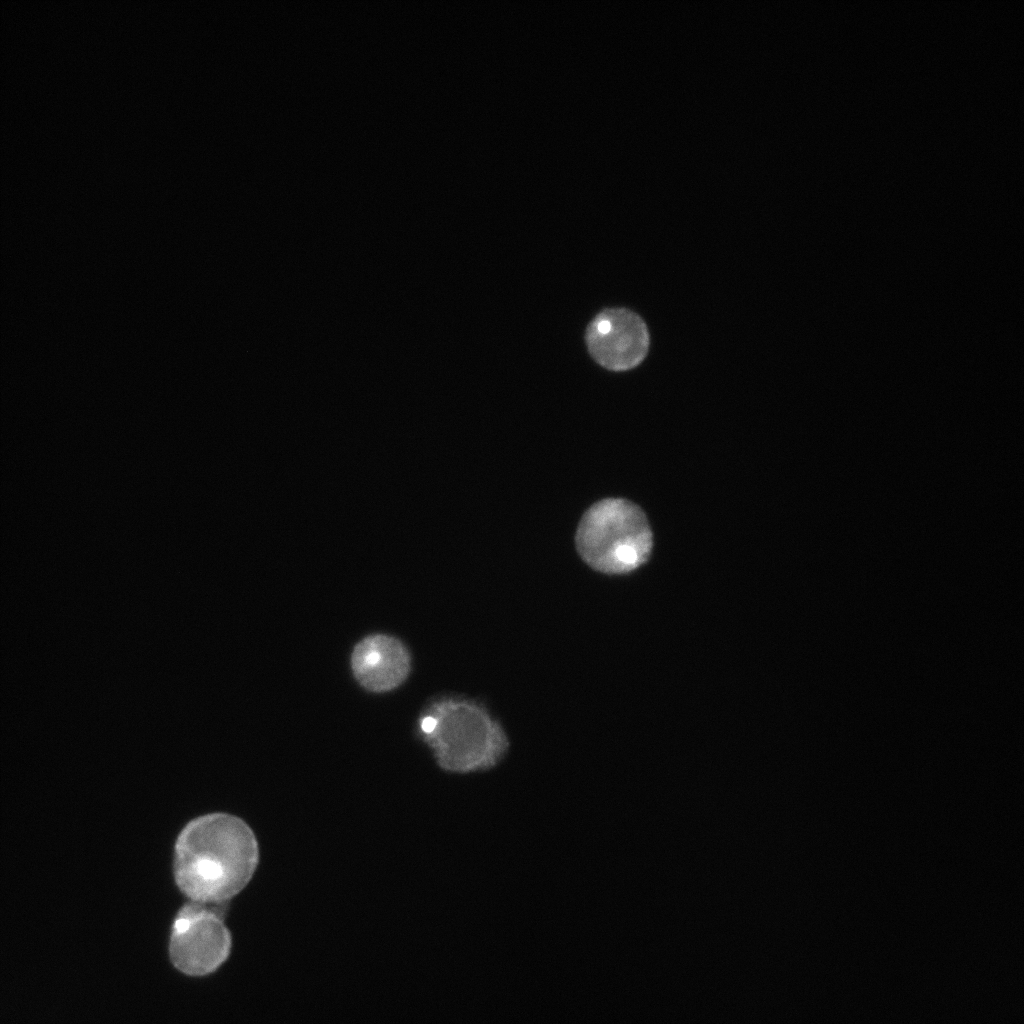

Supplement: Supplementary file 11 — Figure EV5 Source Data [file 44319_2025_533_MOESM11_ESM.zip › Figure EV5/Figure EV5A/Original image Ht31 Pericentrin.tif]

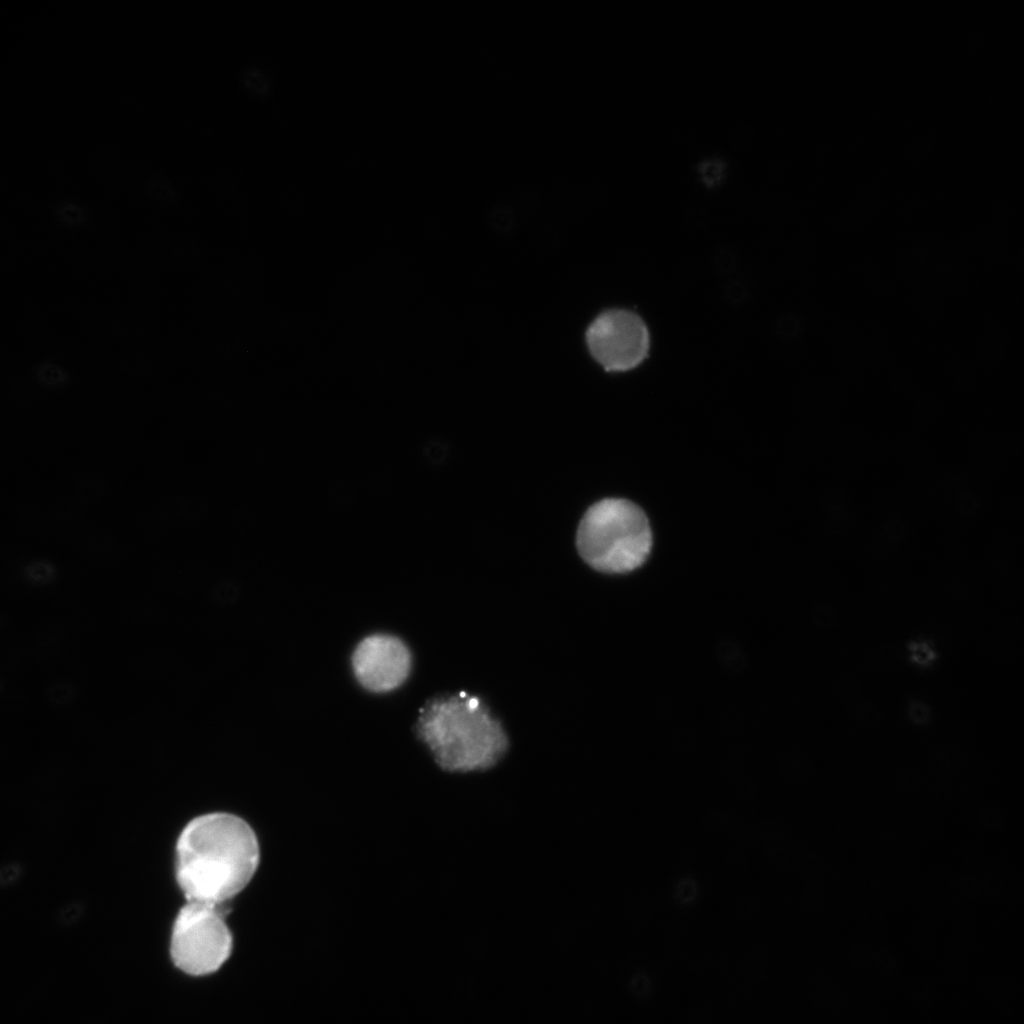

Supplement: Supplementary file 11 — Figure EV5 Source Data [file 44319_2025_533_MOESM11_ESM.zip › Figure EV5/Figure EV5A/Original image Ht31 PKA.tif]

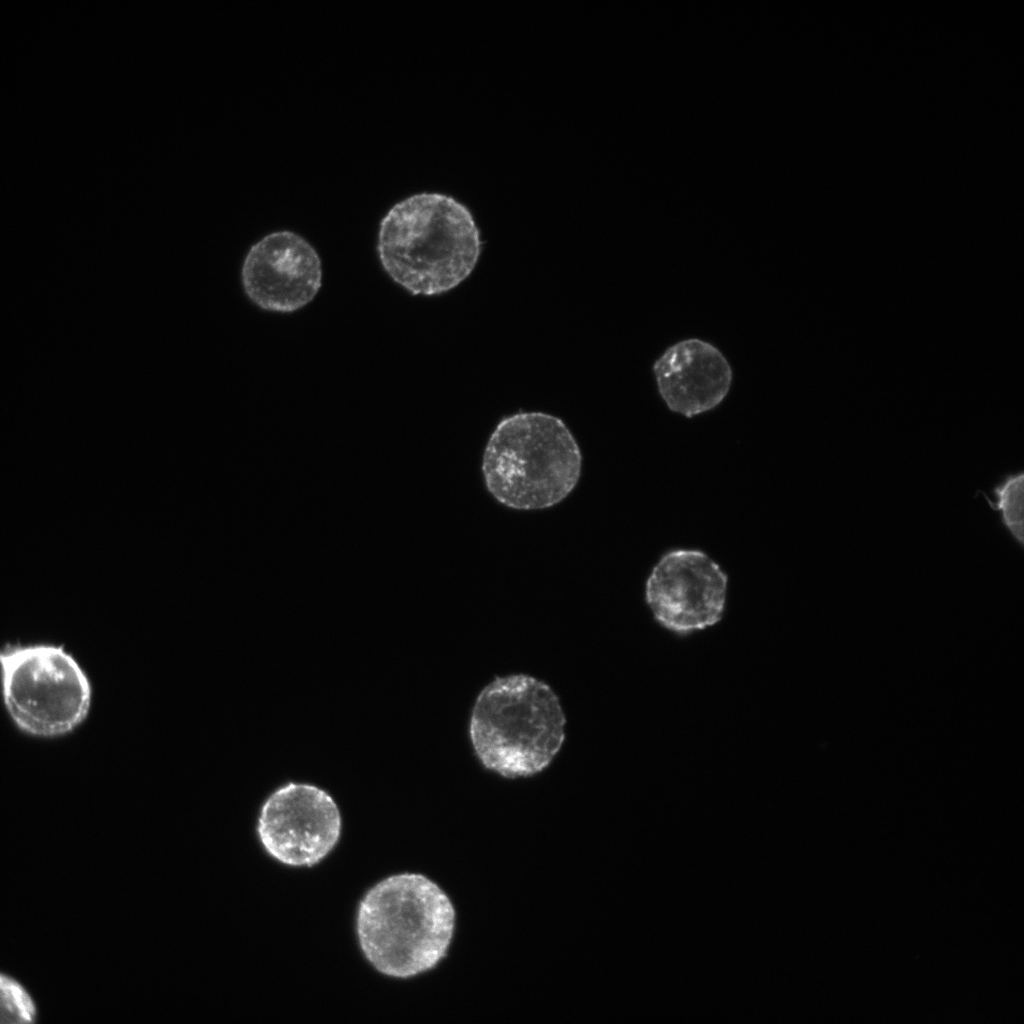

Supplement: Supplementary file 11 — Figure EV5 Source Data [file 44319_2025_533_MOESM11_ESM.zip › Figure EV5/Figure EV5A/Original image Ht31-P Actin.tif]

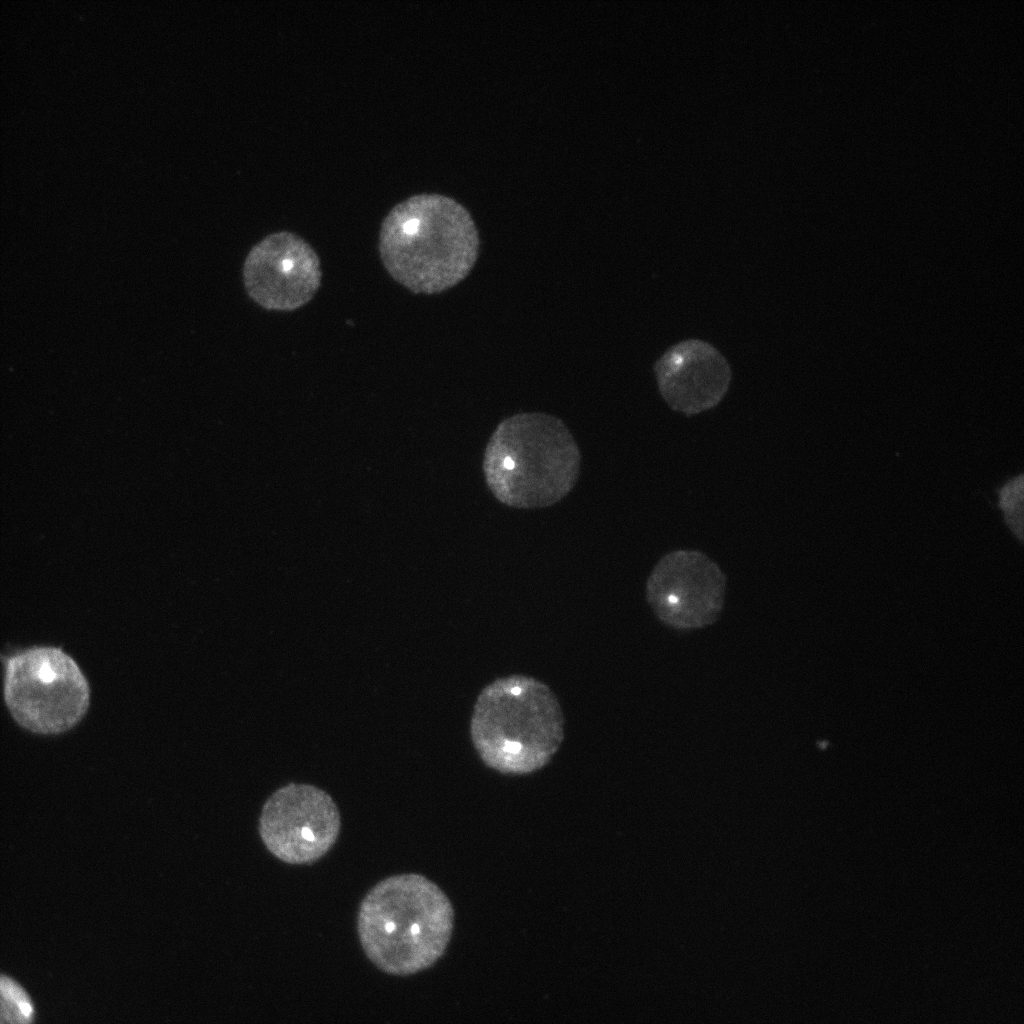

Supplement: Supplementary file 11 — Figure EV5 Source Data [file 44319_2025_533_MOESM11_ESM.zip › Figure EV5/Figure EV5A/Original image Ht31-P Pericentrin.tif]

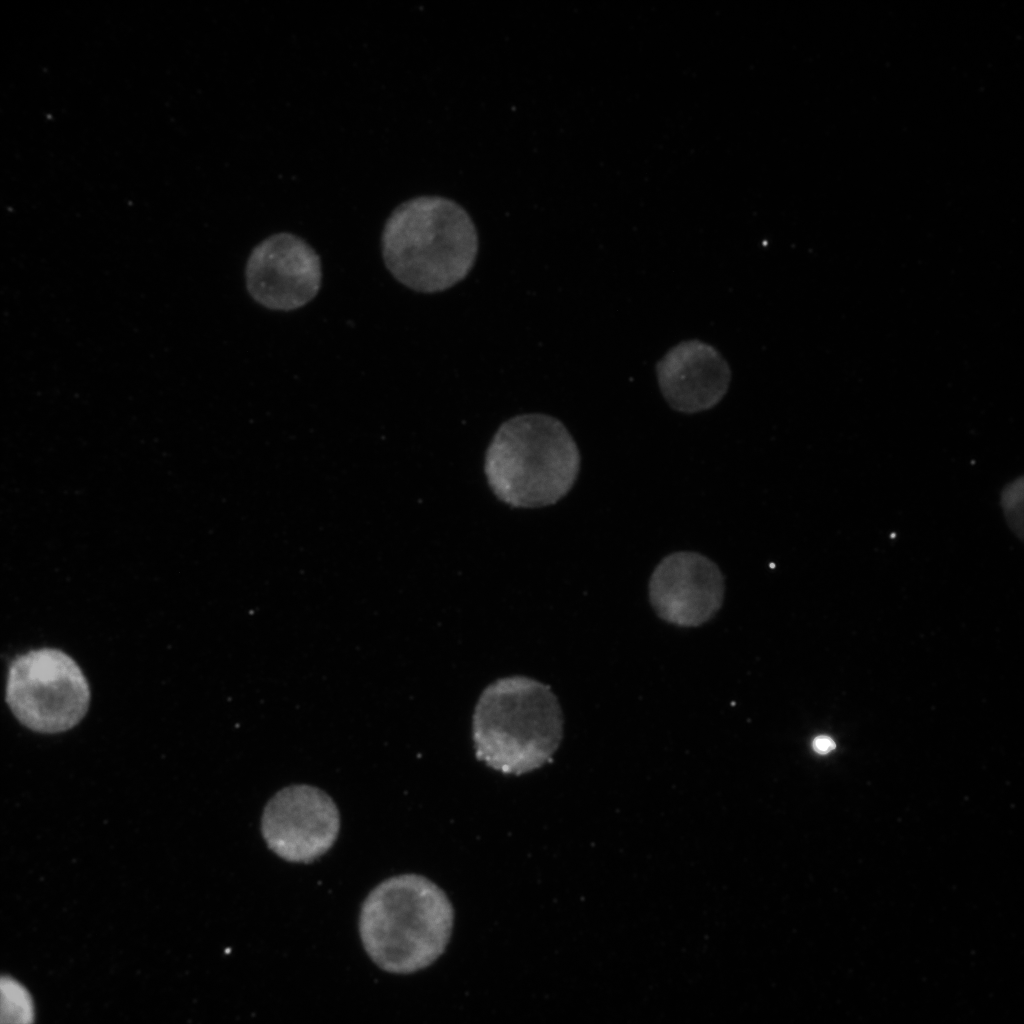

Supplement: Supplementary file 11 — Figure EV5 Source Data [file 44319_2025_533_MOESM11_ESM.zip › Figure EV5/Figure EV5A/Original image Ht31-P PKA.tif]

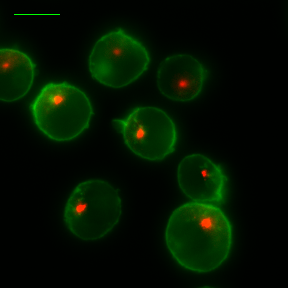

Supplement: Supplementary file 11 — Figure EV5 Source Data [file 44319_2025_533_MOESM11_ESM.zip › Figure EV5/Figure EV5C/Control Actin Pericentrin.tif]

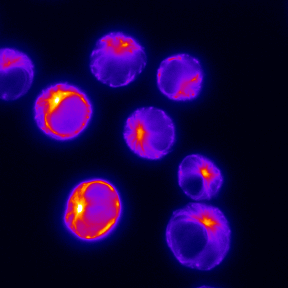

Supplement: Supplementary file 11 — Figure EV5 Source Data [file 44319_2025_533_MOESM11_ESM.zip › Figure EV5/Figure EV5C/Control Microtubule.tif]

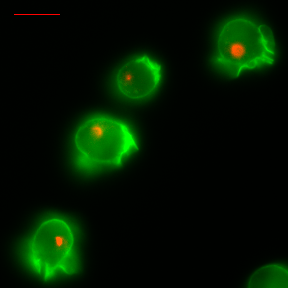

Supplement: Supplementary file 11 — Figure EV5 Source Data [file 44319_2025_533_MOESM11_ESM.zip › Figure EV5/Figure EV5C/CXCL12 Actin Pericentrin.tif]

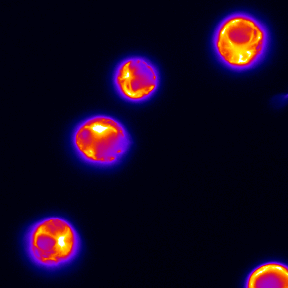

Supplement: Supplementary file 11 — Figure EV5 Source Data [file 44319_2025_533_MOESM11_ESM.zip › Figure EV5/Figure EV5C/CXCL12 Microtubule.tif]

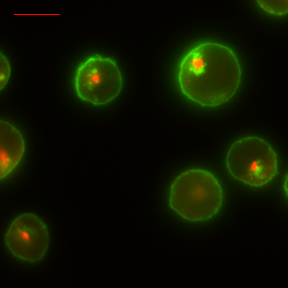

Supplement: Supplementary file 11 — Figure EV5 Source Data [file 44319_2025_533_MOESM11_ESM.zip › Figure EV5/Figure EV5C/H89 Actin Pericentrin.tif]

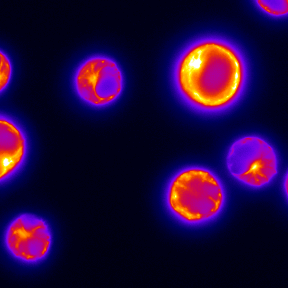

Supplement: Supplementary file 11 — Figure EV5 Source Data [file 44319_2025_533_MOESM11_ESM.zip › Figure EV5/Figure EV5C/H89 Microtubule.tif]

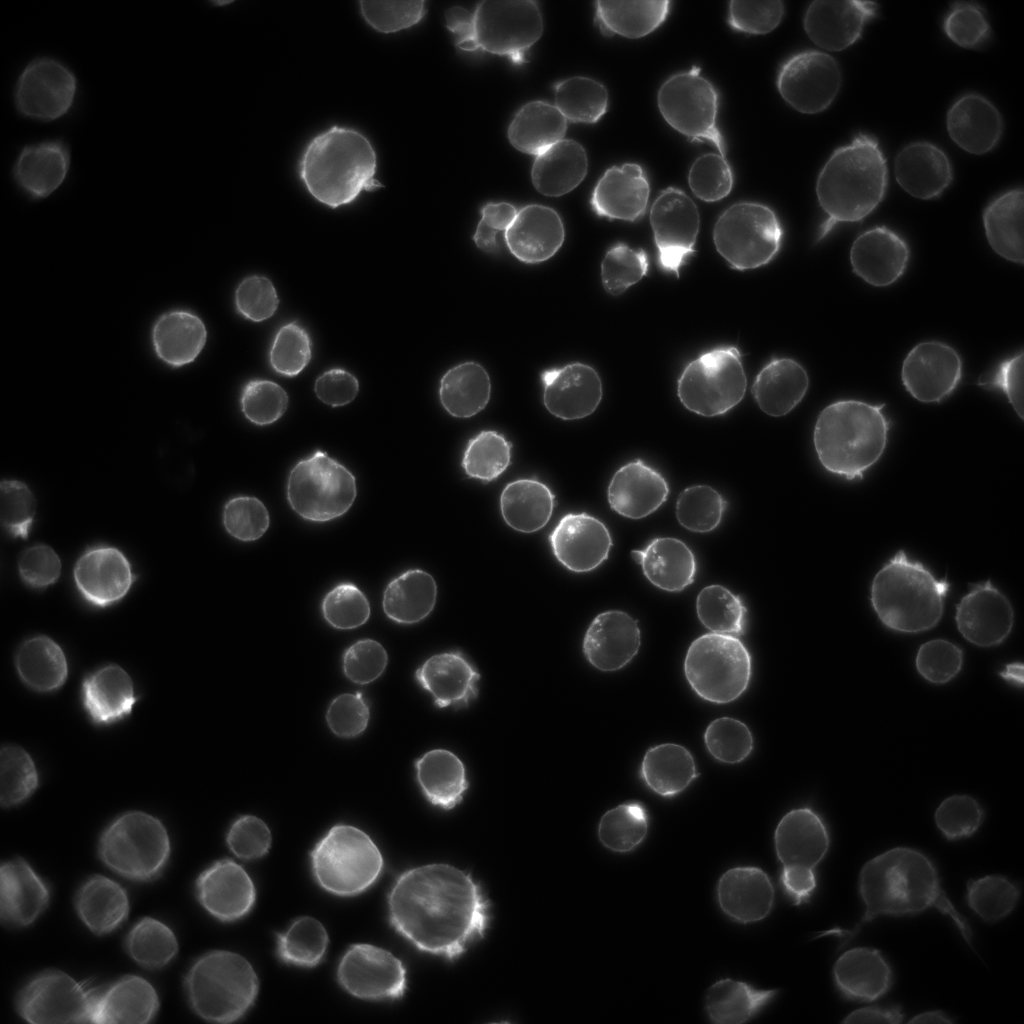

Supplement: Supplementary file 11 — Figure EV5 Source Data [file 44319_2025_533_MOESM11_ESM.zip › Figure EV5/Figure EV5C/Original image Control Actin.tif]

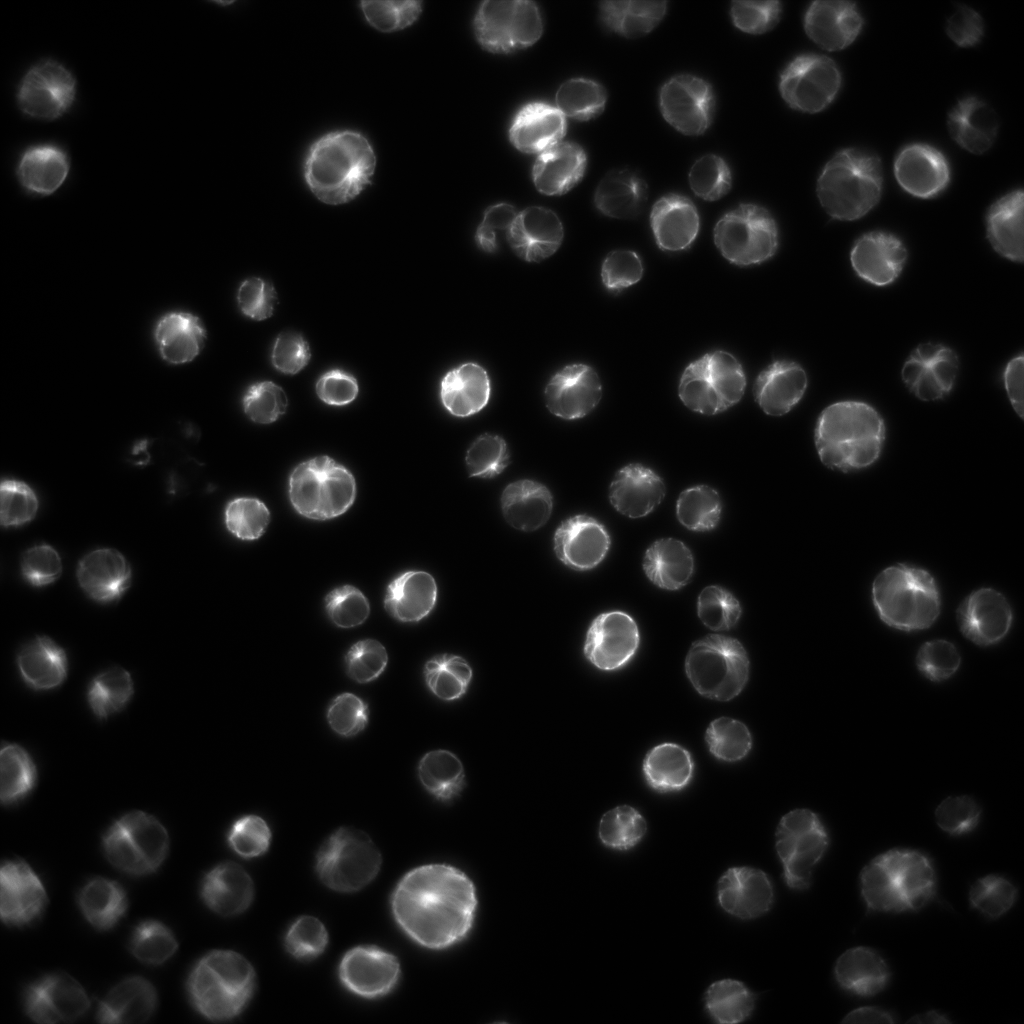

Supplement: Supplementary file 11 — Figure EV5 Source Data [file 44319_2025_533_MOESM11_ESM.zip › Figure EV5/Figure EV5C/Original image Control microtubule.tif]

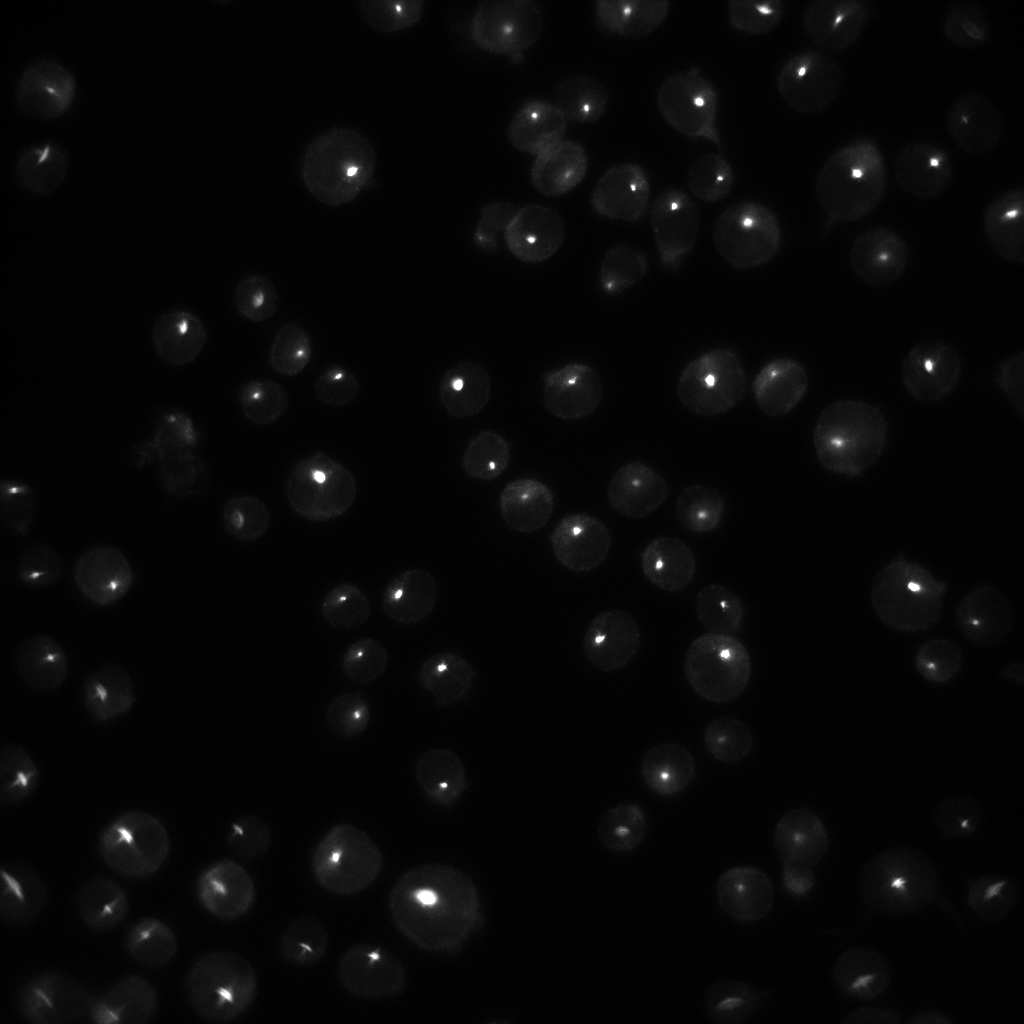

Supplement: Supplementary file 11 — Figure EV5 Source Data [file 44319_2025_533_MOESM11_ESM.zip › Figure EV5/Figure EV5C/Original image Control Pericentrin.tif]

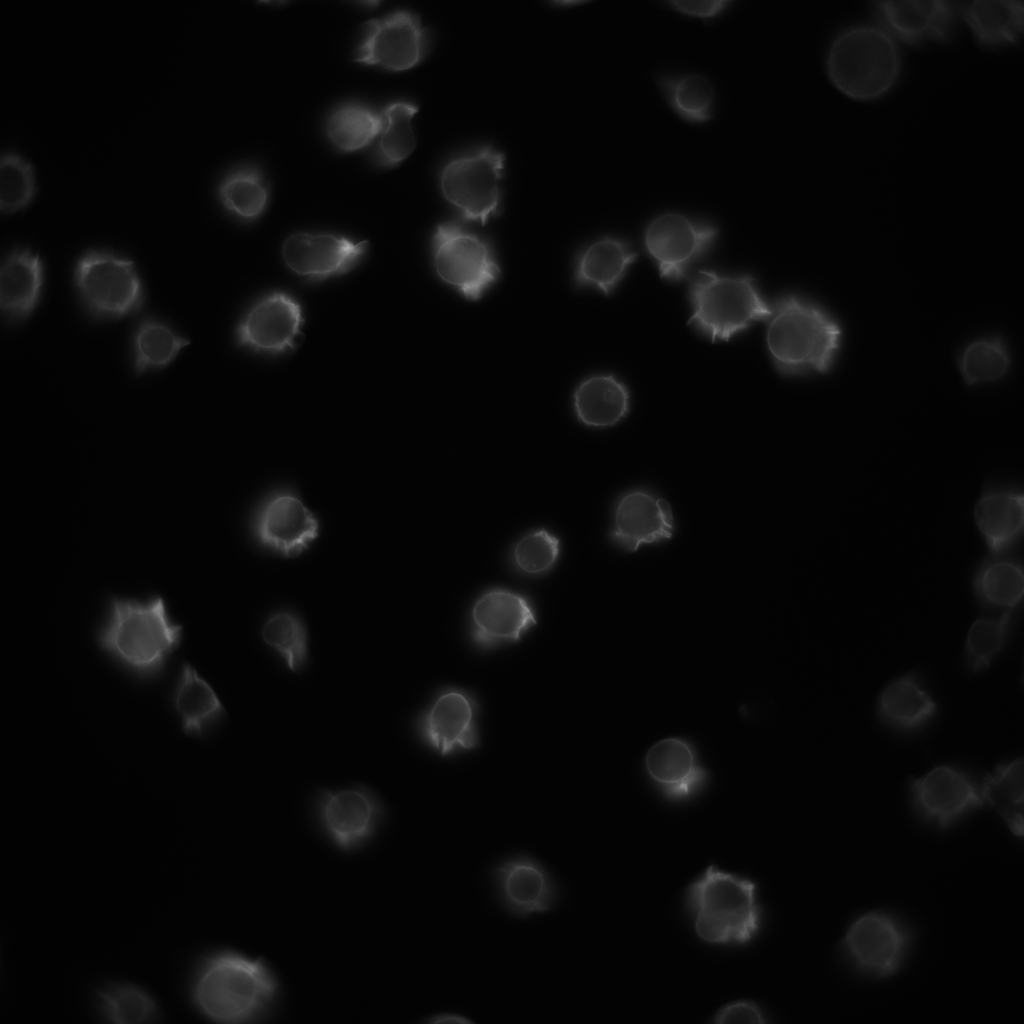

Supplement: Supplementary file 11 — Figure EV5 Source Data [file 44319_2025_533_MOESM11_ESM.zip › Figure EV5/Figure EV5C/Original image CXCL12 Actin.tif]

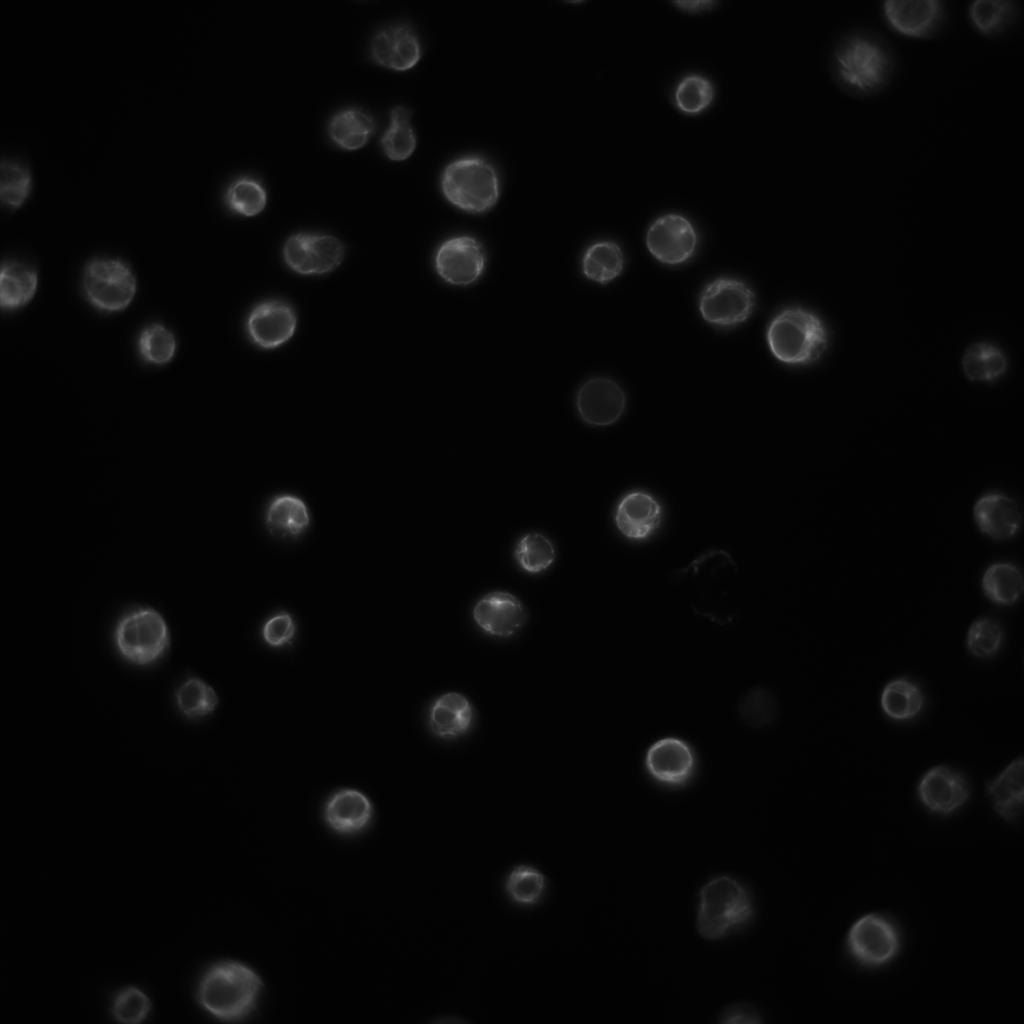

Supplement: Supplementary file 11 — Figure EV5 Source Data [file 44319_2025_533_MOESM11_ESM.zip › Figure EV5/Figure EV5C/Original image CXCL12 Microtubule.tif]

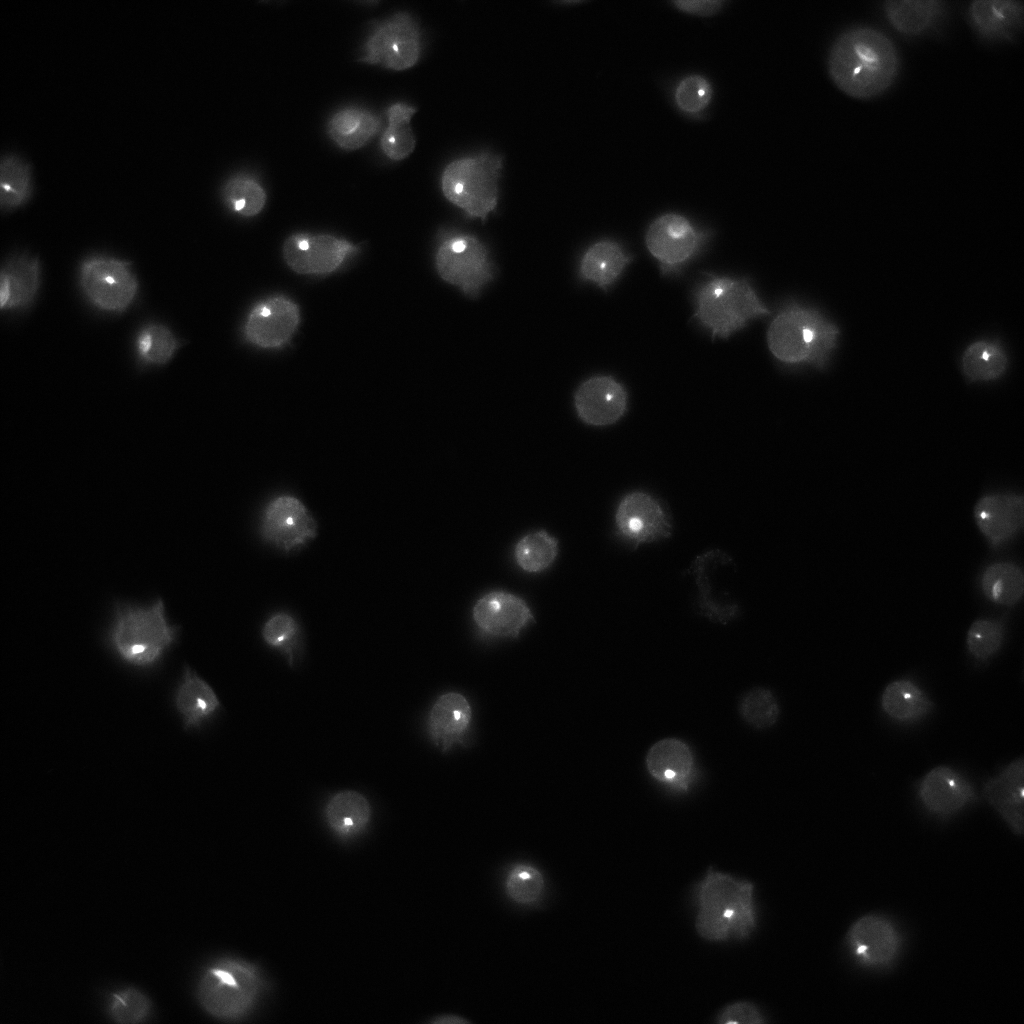

Supplement: Supplementary file 11 — Figure EV5 Source Data [file 44319_2025_533_MOESM11_ESM.zip › Figure EV5/Figure EV5C/Original image CXCL12 Pericentrin.tif]

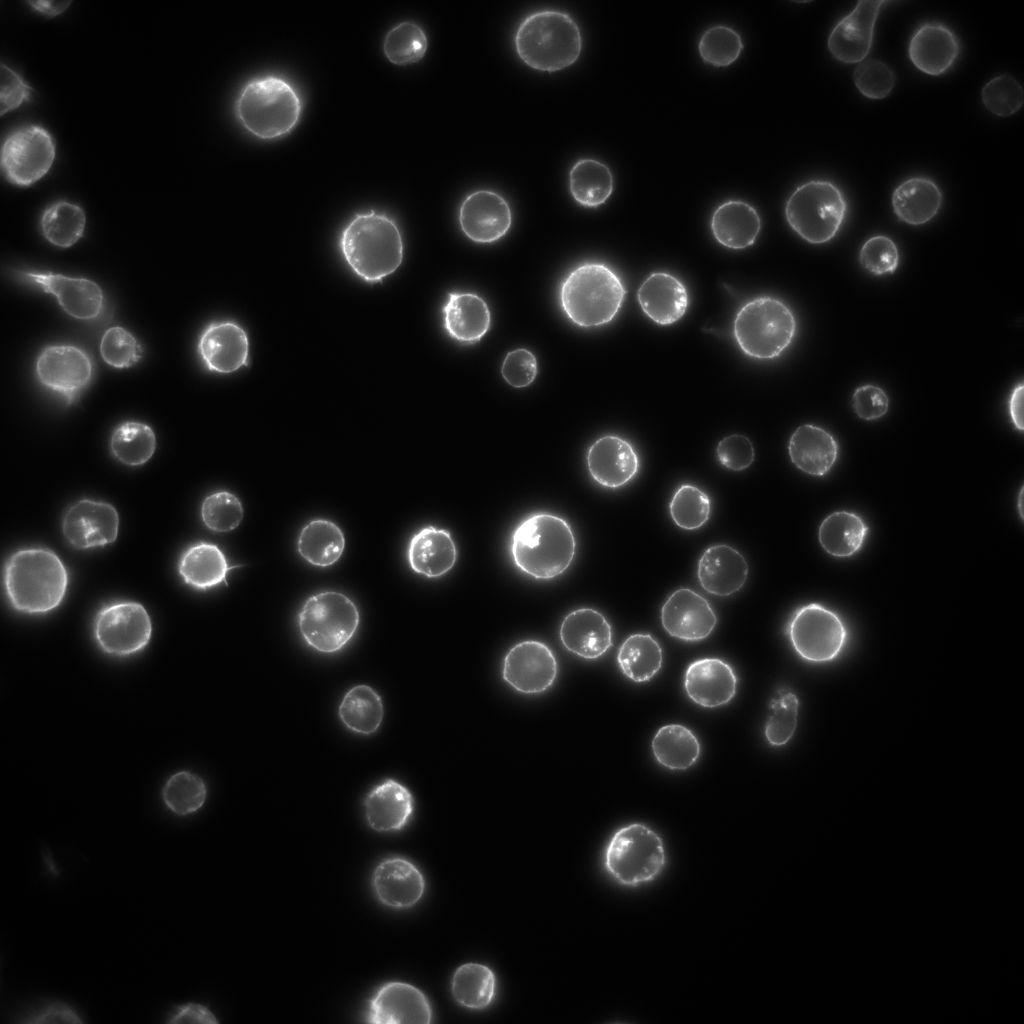

Supplement: Supplementary file 11 — Figure EV5 Source Data [file 44319_2025_533_MOESM11_ESM.zip › Figure EV5/Figure EV5C/Original image H89 Actin.tif]

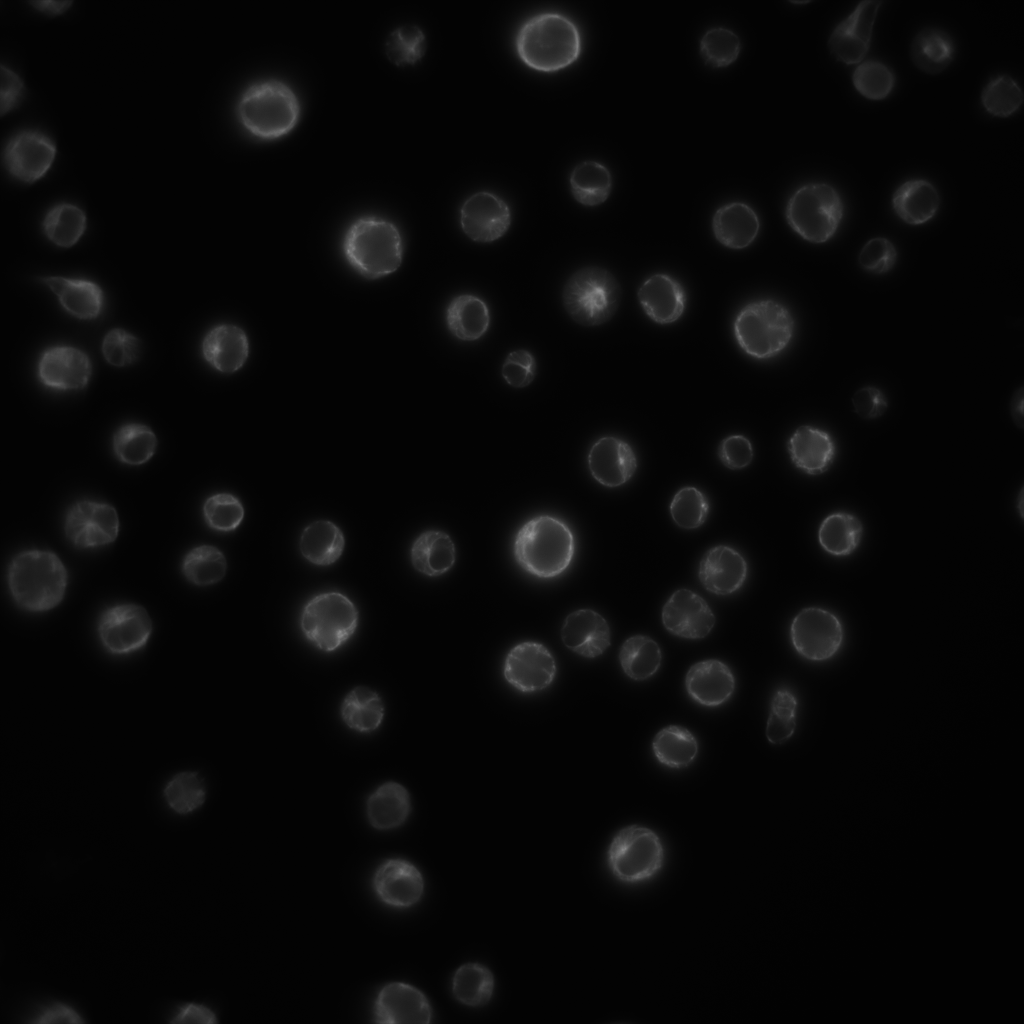

Supplement: Supplementary file 11 — Figure EV5 Source Data [file 44319_2025_533_MOESM11_ESM.zip › Figure EV5/Figure EV5C/Original image H89 Microtubule.tif]

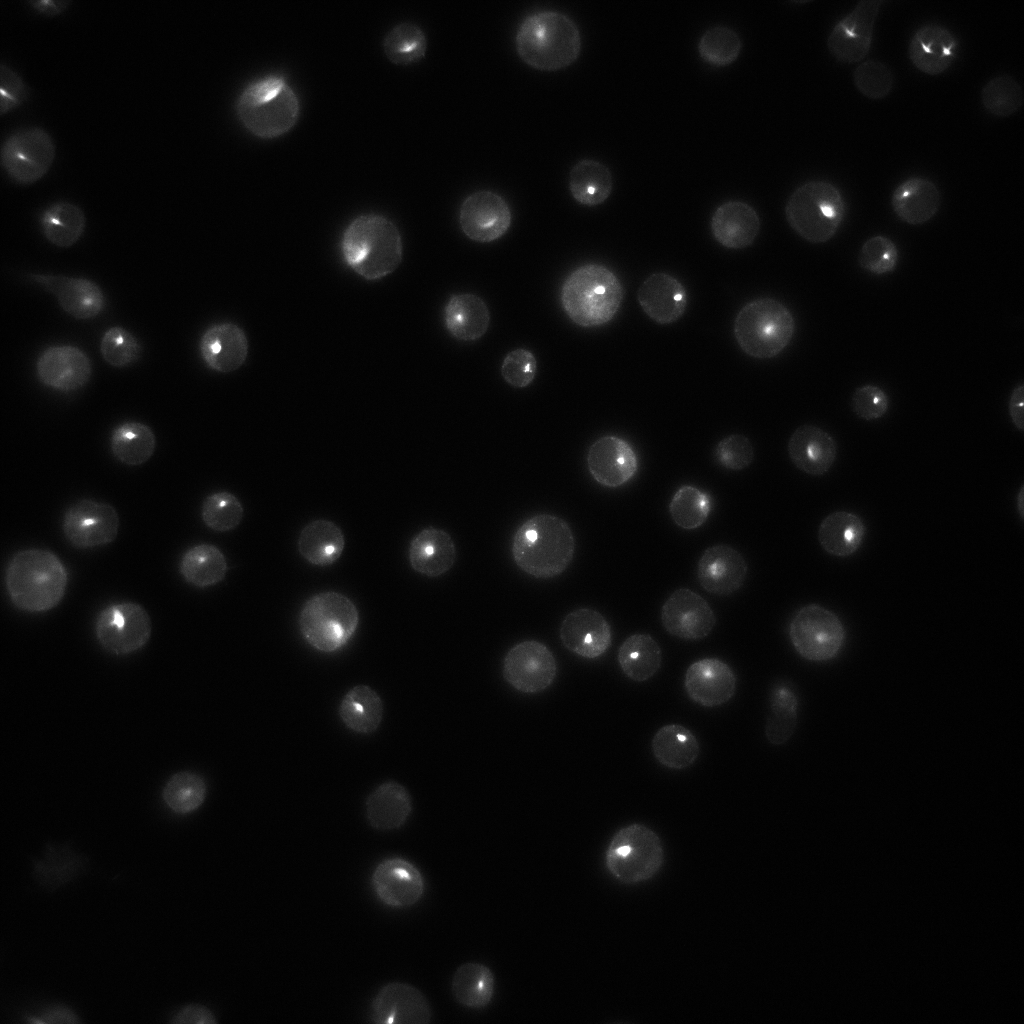

Supplement: Supplementary file 11 — Figure EV5 Source Data [file 44319_2025_533_MOESM11_ESM.zip › Figure EV5/Figure EV5C/Original image H89 Pericentrin.tif]

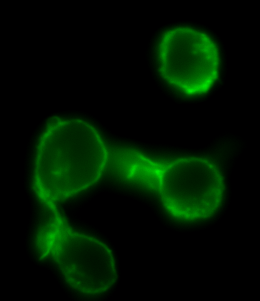

Supplement: Supplementary file 11 — Figure EV5 Source Data [file 44319_2025_533_MOESM11_ESM.zip › Figure EV5/Figure EV5D/Centrin-GFP Actin.tif]

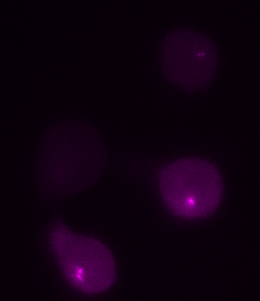

Supplement: Supplementary file 11 — Figure EV5 Source Data [file 44319_2025_533_MOESM11_ESM.zip › Figure EV5/Figure EV5D/Centrin-GFP GFP.tif]

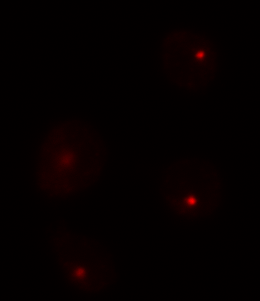

Supplement: Supplementary file 11 — Figure EV5 Source Data [file 44319_2025_533_MOESM11_ESM.zip › Figure EV5/Figure EV5D/Centrin-GFP Pericentrin.tif]

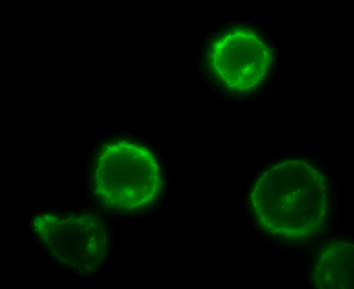

Supplement: Supplementary file 11 — Figure EV5 Source Data [file 44319_2025_533_MOESM11_ESM.zip › Figure EV5/Figure EV5D/Centrin-VCA-GFP Actin.tif]

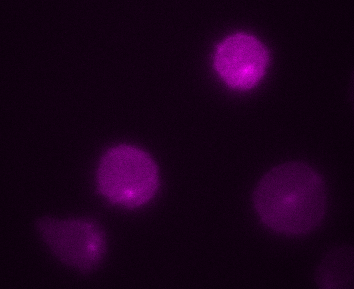

Supplement: Supplementary file 11 — Figure EV5 Source Data [file 44319_2025_533_MOESM11_ESM.zip › Figure EV5/Figure EV5D/Centrin-VCA-GFP GFP.tif]

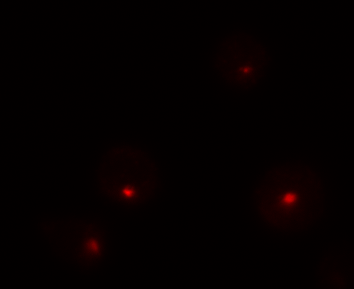

Supplement: Supplementary file 11 — Figure EV5 Source Data [file 44319_2025_533_MOESM11_ESM.zip › Figure EV5/Figure EV5D/Centrin-VCA-GFP Pericentrin.tif]

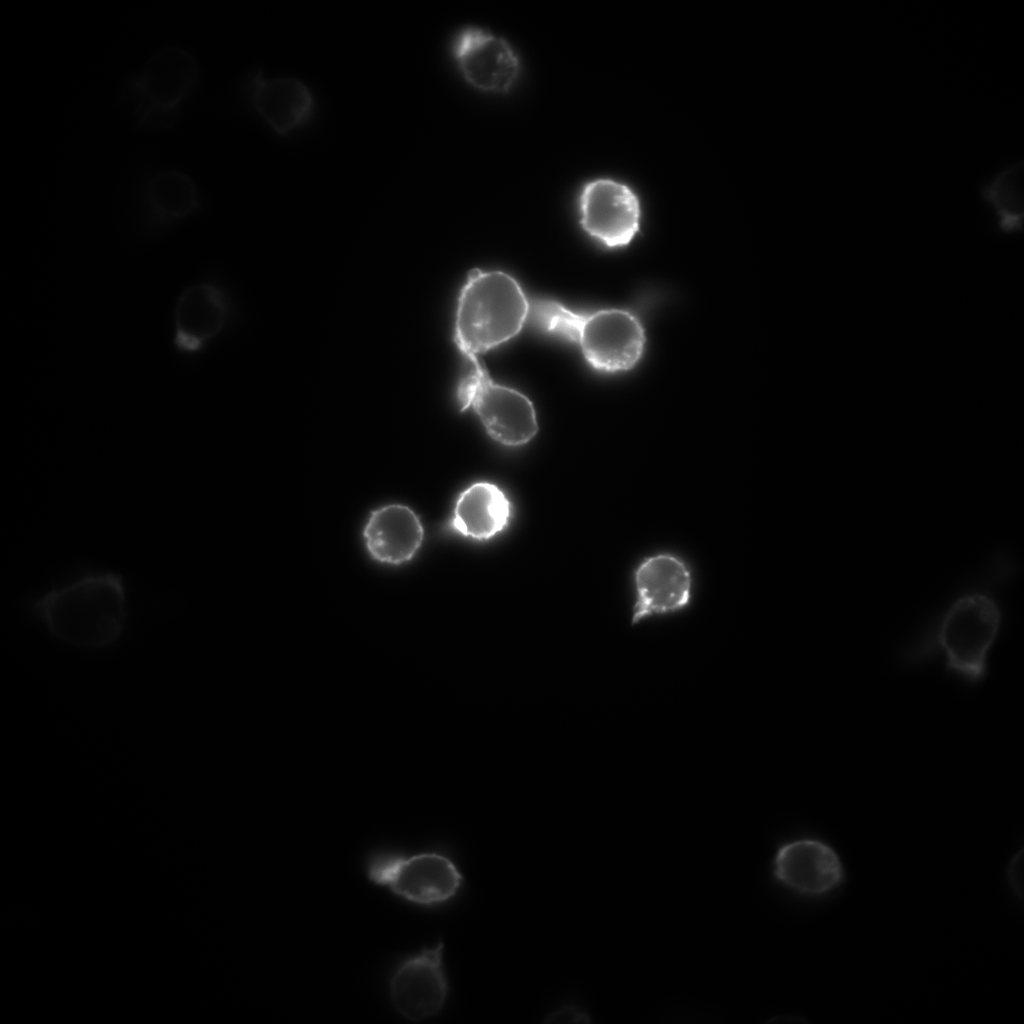

Supplement: Supplementary file 11 — Figure EV5 Source Data [file 44319_2025_533_MOESM11_ESM.zip › Figure EV5/Figure EV5D/Original image Centrin-GFP Actin.tif]

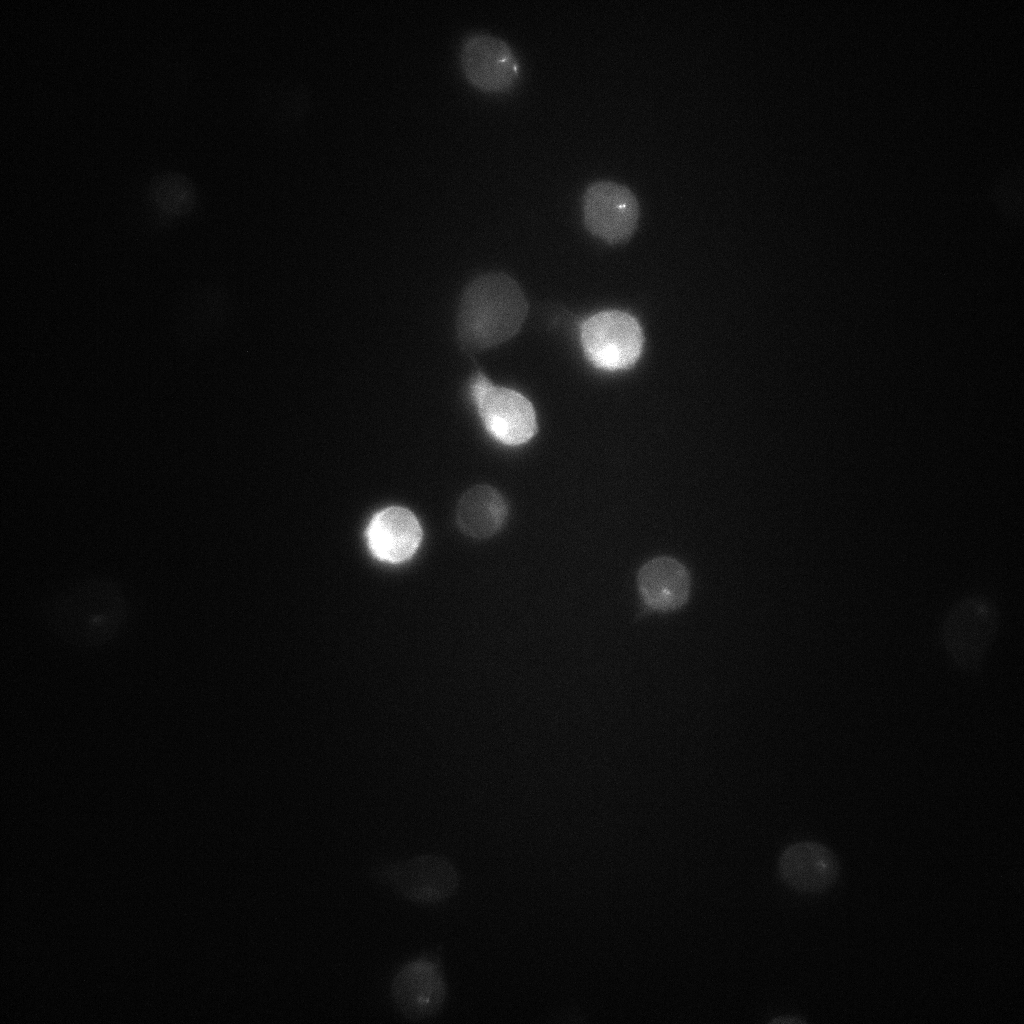

Supplement: Supplementary file 11 — Figure EV5 Source Data [file 44319_2025_533_MOESM11_ESM.zip › Figure EV5/Figure EV5D/Original image Centrin-GFP GFP.tif]

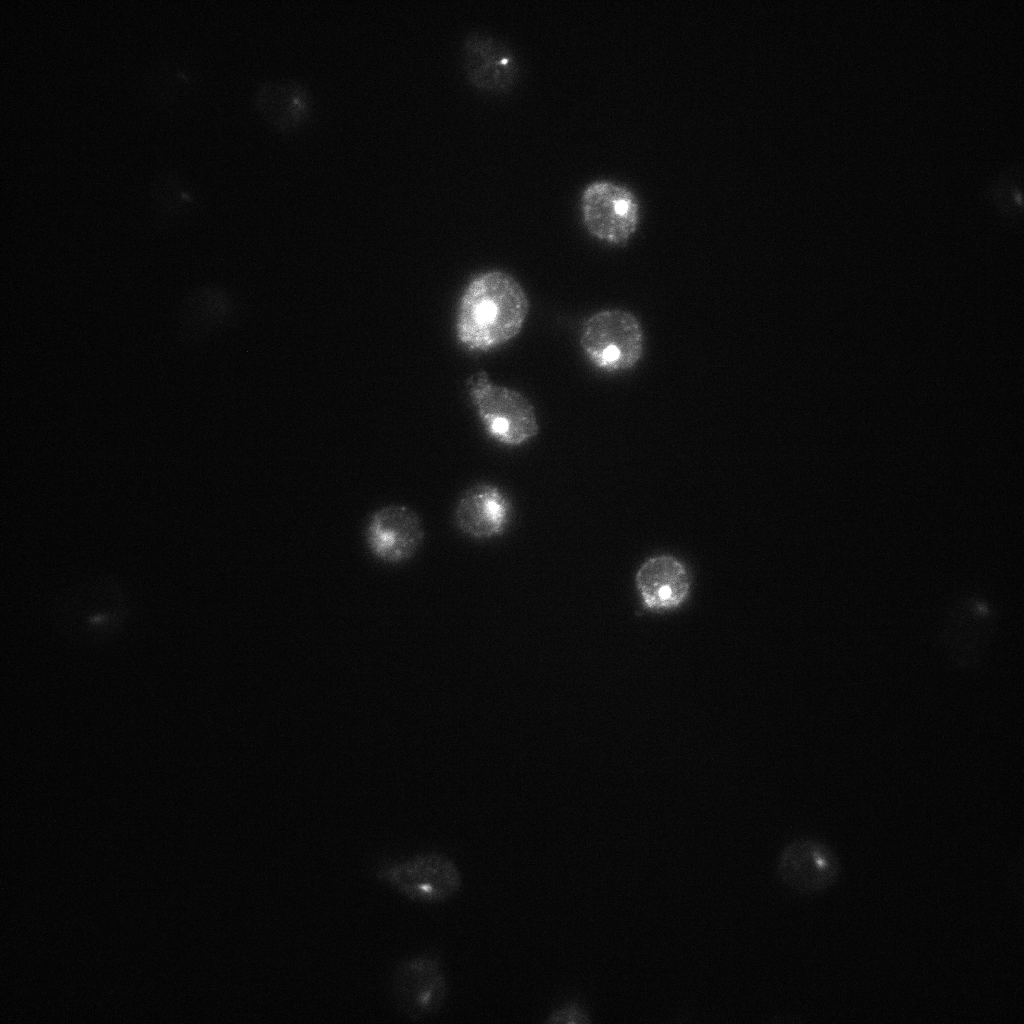

Supplement: Supplementary file 11 — Figure EV5 Source Data [file 44319_2025_533_MOESM11_ESM.zip › Figure EV5/Figure EV5D/Original image Centrin-GFP Pericentrin.tif]

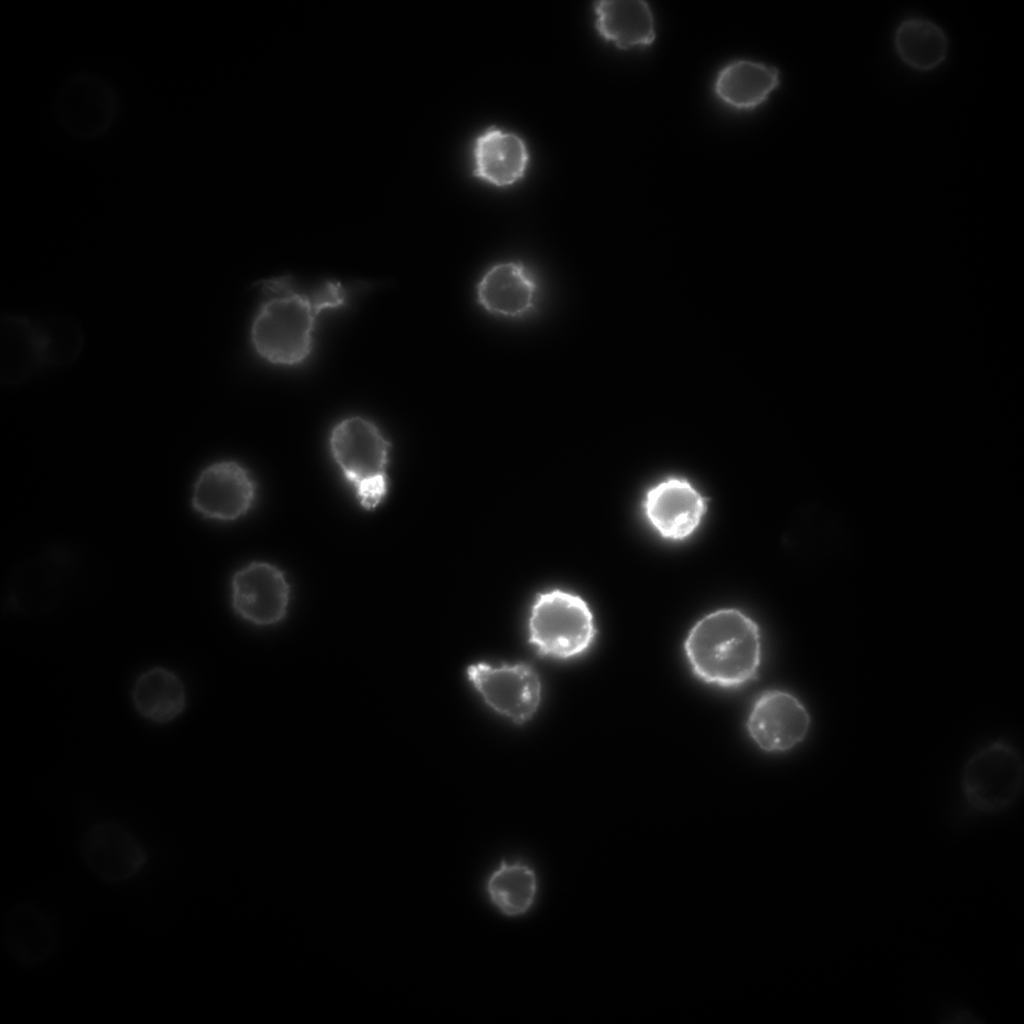

Supplement: Supplementary file 11 — Figure EV5 Source Data [file 44319_2025_533_MOESM11_ESM.zip › Figure EV5/Figure EV5D/Original image Centrin-VCA-GFP Actin.tif]

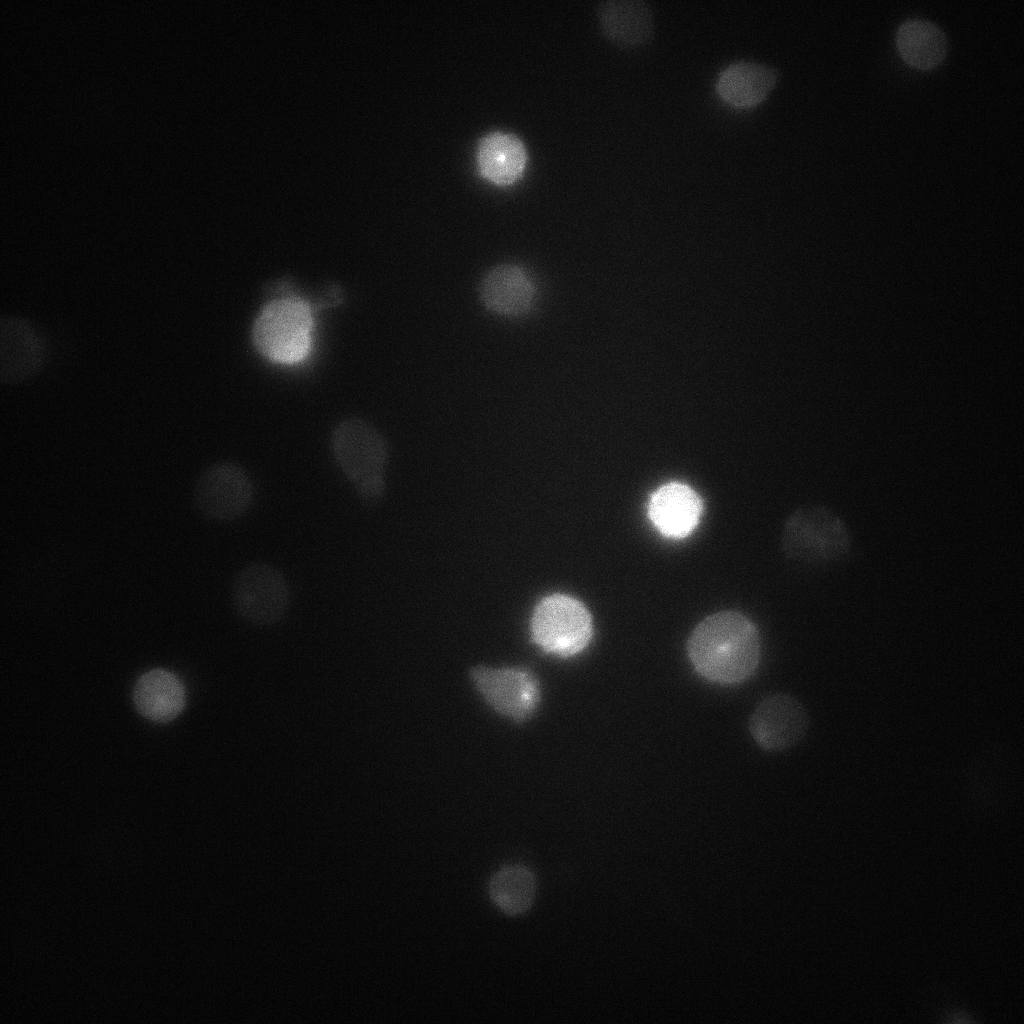

Supplement: Supplementary file 11 — Figure EV5 Source Data [file 44319_2025_533_MOESM11_ESM.zip › Figure EV5/Figure EV5D/Original image Centrin-VCA-GFP GFP.tif]

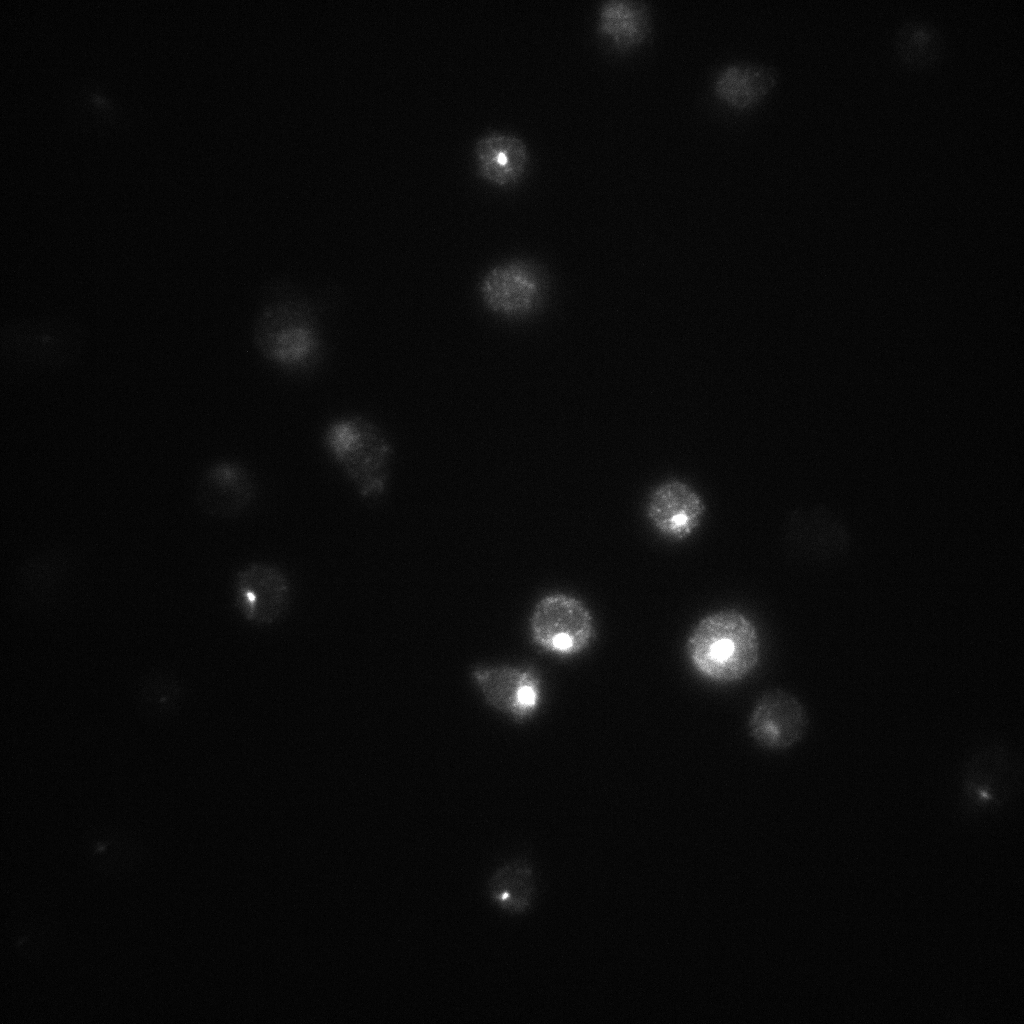

Supplement: Supplementary file 11 — Figure EV5 Source Data [file 44319_2025_533_MOESM11_ESM.zip › Figure EV5/Figure EV5D/Original image Centrin-VCA-GFP Pericentrin.tif]

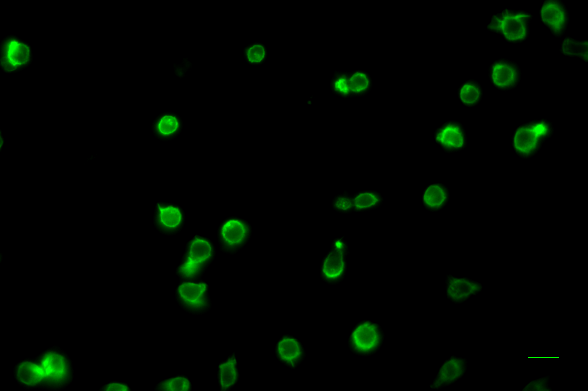

Supplement: Supplementary file 11 — Figure EV5 Source Data [file 44319_2025_533_MOESM11_ESM.zip › Figure EV5/Figure EV5E/Centrin-GFP Actin.tif]

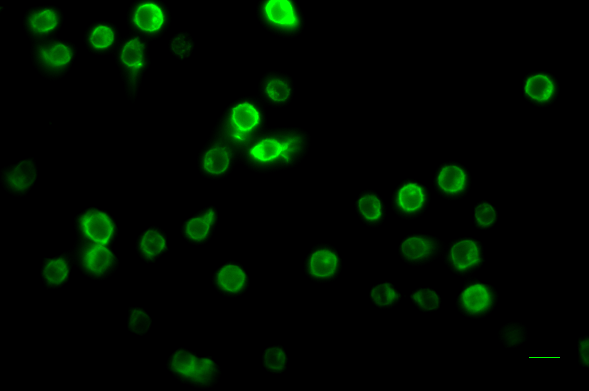

Supplement: Supplementary file 11 — Figure EV5 Source Data [file 44319_2025_533_MOESM11_ESM.zip › Figure EV5/Figure EV5E/Centrin-VCA-GFP Actin.tif]

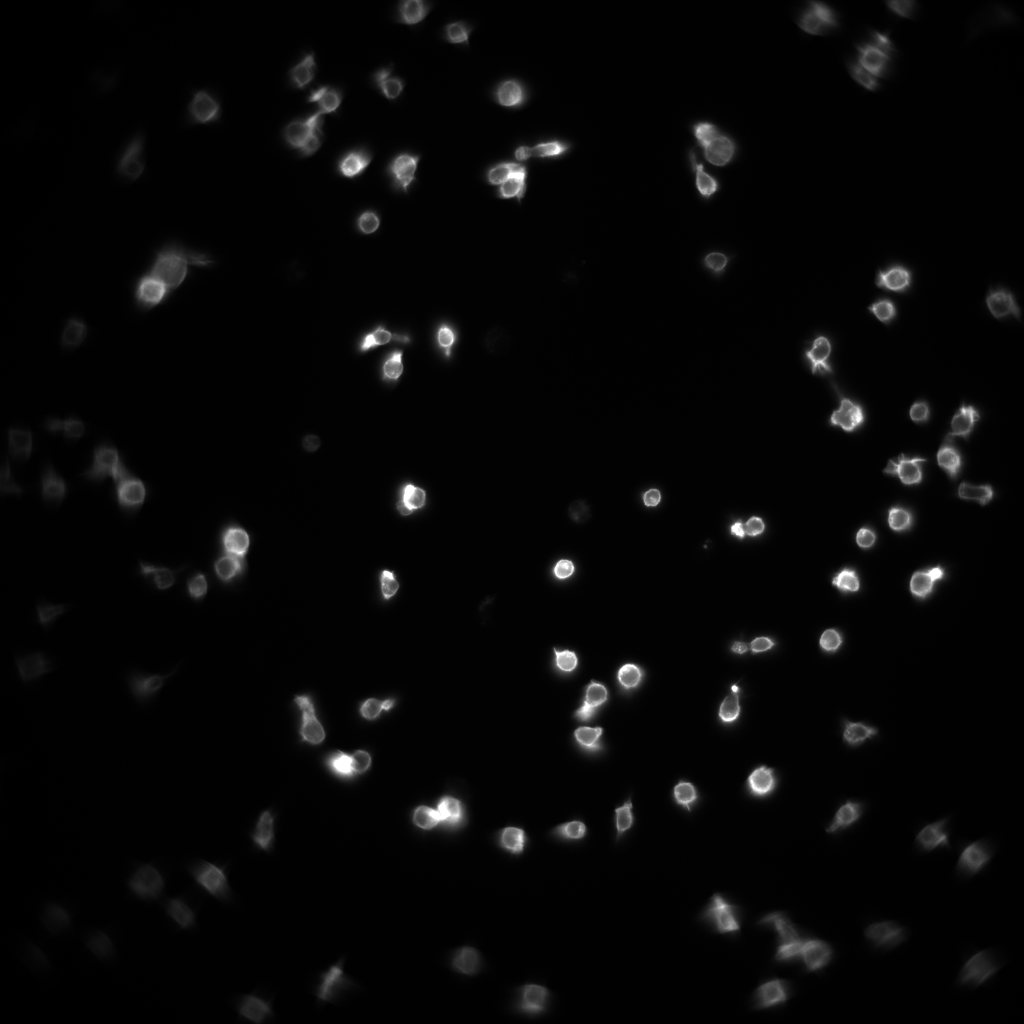

Supplement: Supplementary file 11 — Figure EV5 Source Data [file 44319_2025_533_MOESM11_ESM.zip › Figure EV5/Figure EV5E/Original Image Centrin-GFP Actin.tif]

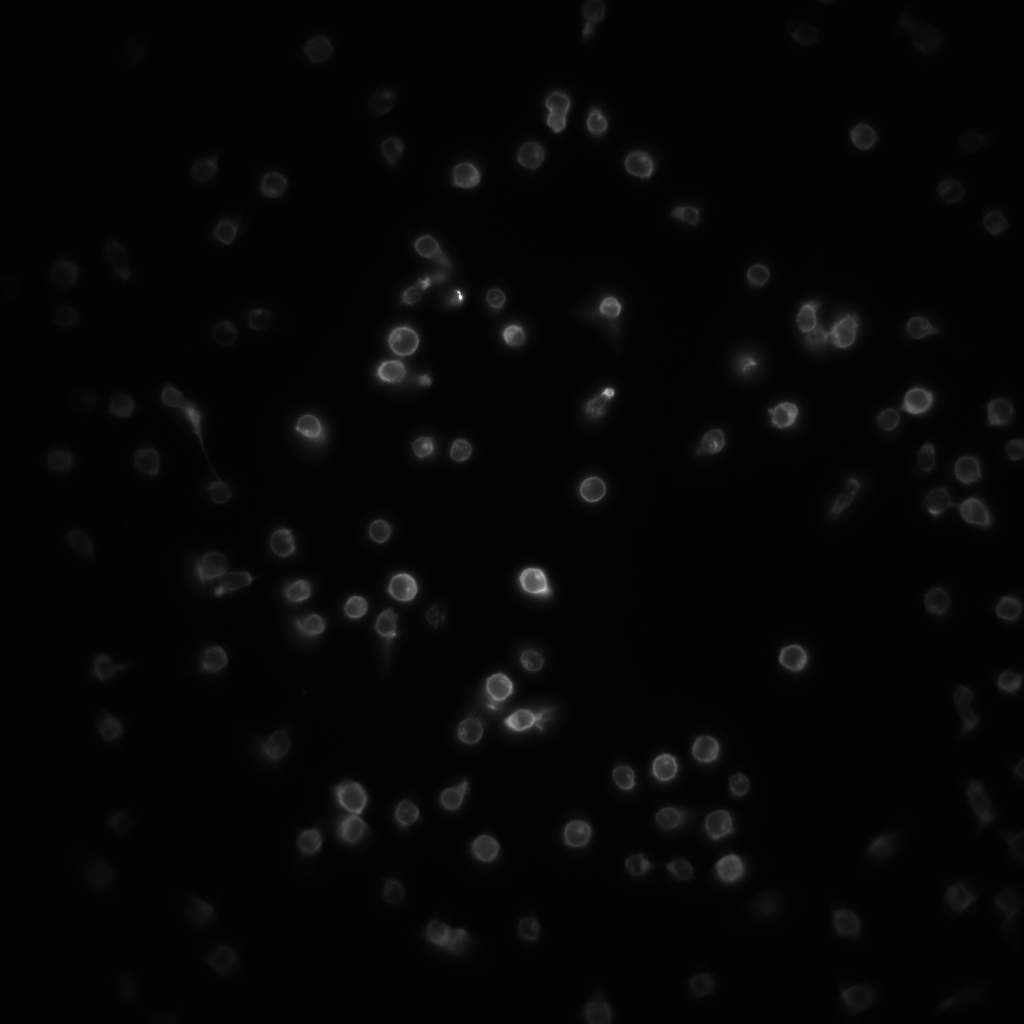

Supplement: Supplementary file 11 — Figure EV5 Source Data [file 44319_2025_533_MOESM11_ESM.zip › Figure EV5/Figure EV5E/Original image Centrin-VCA-GFP Actin.tif]
