# Supplementary material for: Centrosomal actin pool levels regulated by localized PKA set the threshold for T cell polarization
Source: EMBO Rep. 2025 Aug 26;26(18):4436–55. doi: 10.1038/s44319-025-00533-2 (PMC12457651; doi:10.1038/s44319-025-00533-2)
Supplement: Supplementary file 12 — Expanded View Figures [file 44319_2025_533_MOESM12_ESM.pdf]

Expanded View Figures

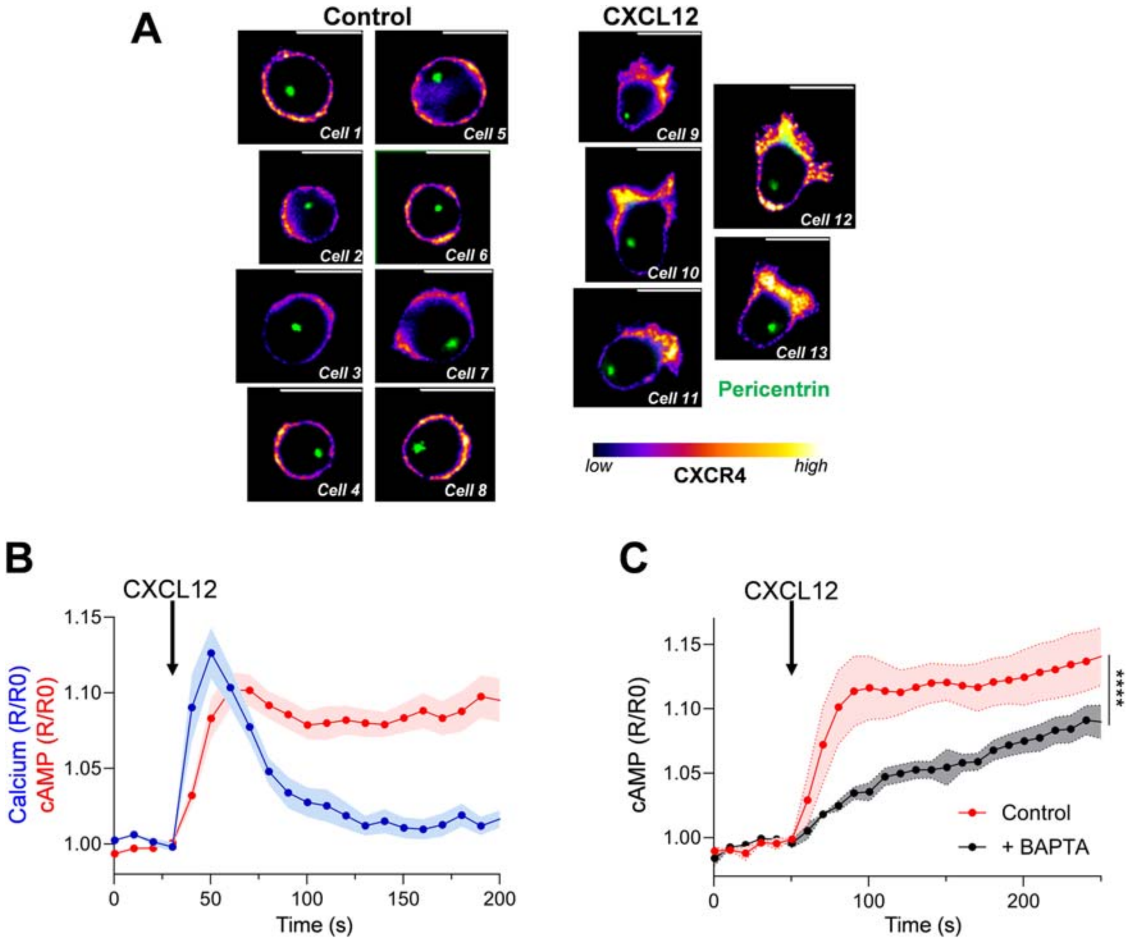

Figure EV1. Distribution of CXCR4 and variations of Calcium and cAMP upon CXCL12 stimulation.

(**A**) Example of CXCR4 distribution revealed by immunofluorescence in eight different unstimulated (left) and five different CXCL12-stimulated (right) CEM T cells. The centrosome is labeled with an antibody against pericentrin (green). Scale bar = 10 μm. (**B**) Simultaneous measurements of Ca and cAMP variations in CEM T cells transfected with TEpacVV and loaded with Fura-2. CXCL12 stimulation is indicated by the black arrow. For both parameters, values have been normalized relative to the value at the time of stimulation. Mean ± SE of 13 cells. (**C**) CXCL12-induced cAMP variations in CEM T cells transfected with TEpacVV and preincubated or not with the Ca chelator BAPTA/AM. Values have been normalized relatively to the Ca value at the time of stimulation. Mean ± SE of five independent experiments, 6–19 cells/condition/experiment. Statistics: Two-way Anova. ****$p < 0.0001$. Source data are available online for this figure.

© The Author(s)

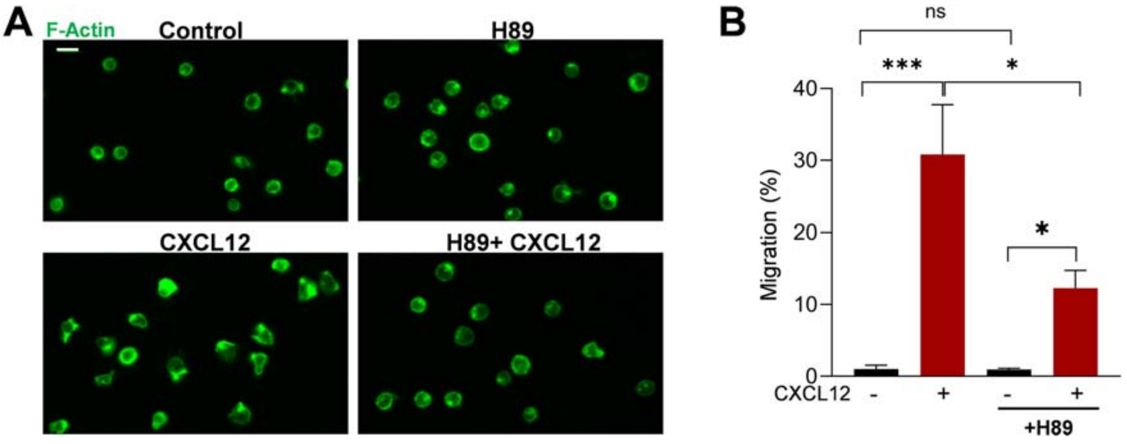

Figure EV2. Involvement of PKA in CXCL12-induced CEM T cell deformation and migration.

(**A**) Example of morphology of CEM T cell stimulated or not with CXCL12 and with or without pretreatment with H89. F-Actin (green) has been labeled with Phalloidin. Scale bar = 20 µm. (**B**) Transwell migration assay with CEM T cells pretreated or not with H89. When indicated, CXCL12 was added to the lower compartment. Mean ± SE of three independent experiments. Statistics: Nonparametric ANOVA test for multiple comparisons. ***$p \leq 0.001$, *$p < 0.041$. Source data are available online for this figure.

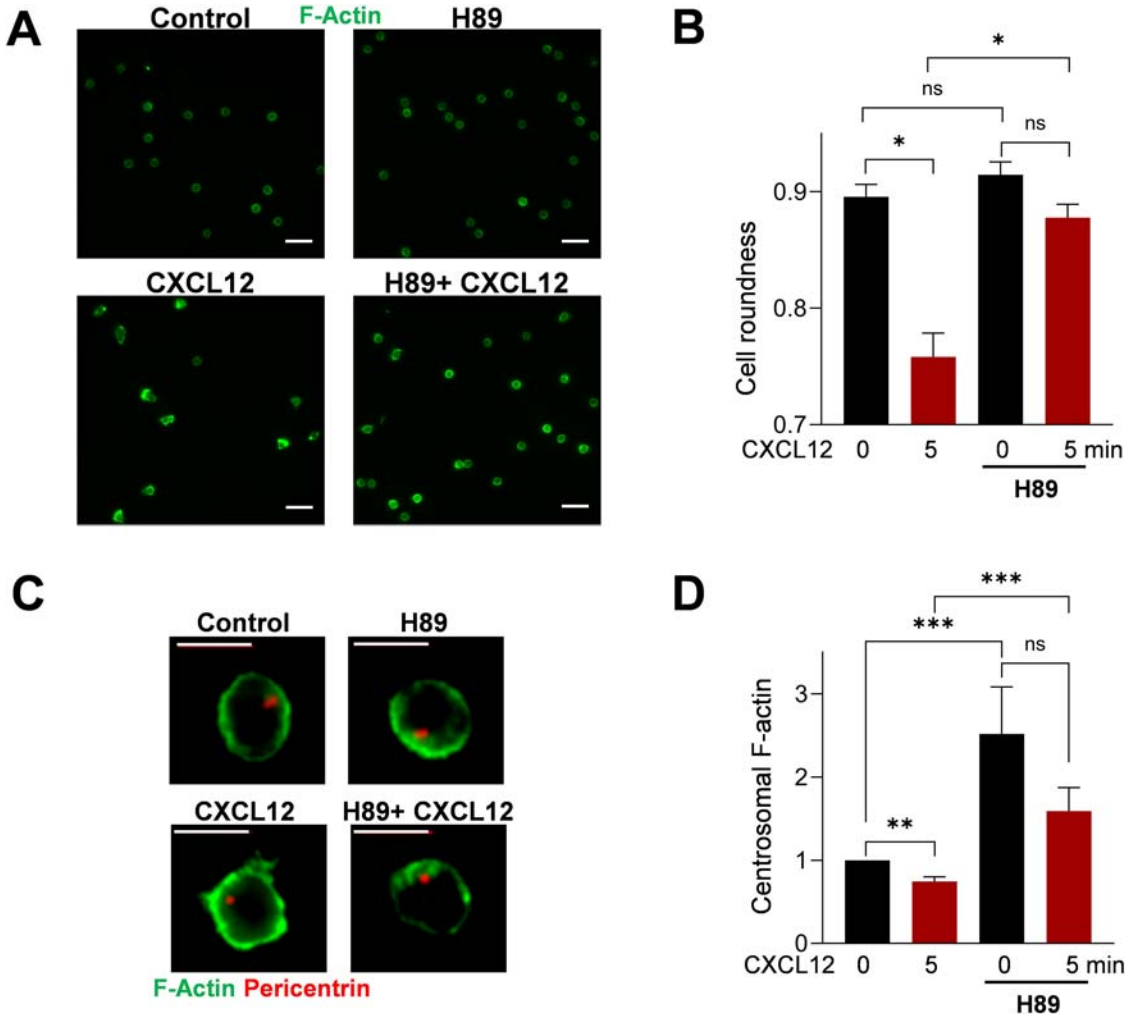

Figure EV3. Sensitivity of CXCL12-stimulated human primary blood T cells to PKA inhibitor.

(**A**) Example of morphology of PBT stimulated or not with CXCL12 and with or without pretreatment with H89. F-Actin (green) has been labeled with Phalloidin. Scale bar = 20 µm. (**B**) Quantification of cell roundness of PBT stimulated or not with CXCL12 and with or without pretreatment with H89. Mean ± SE of four independent experiments (four different donors), 64–871 cells/condition/experiment. Statistics: Nonparametric ANOVA test for multiple comparisons. *$p = 0.017$. (**C**) Example of F-actin distribution in PBT stimulated or not with CXCL12 and with or without pretreatment with H89. F-actin is labeled with Phalloidin (green) and centrosome with an antibody against pericentrin (red). Scale bar = 20 µm. (**D**) Quantification of centrosomal actin in PBT stimulated or not with CXCL12 and with or without pretreatment with H89. For each experiment, values have been normalized relative to the mean intensity of centrosomal F-actin measured in unstimulated and untreated PBT. Mean ± SE of four independent experiments (four different donors), 11–57 cells/condition/experiment. Statistics: Nonparametric ANOVA test for multiple comparisons. ***$p < 0.001$, **$p = 0.007$. Source data are available online for this figure.

© The Author(s)

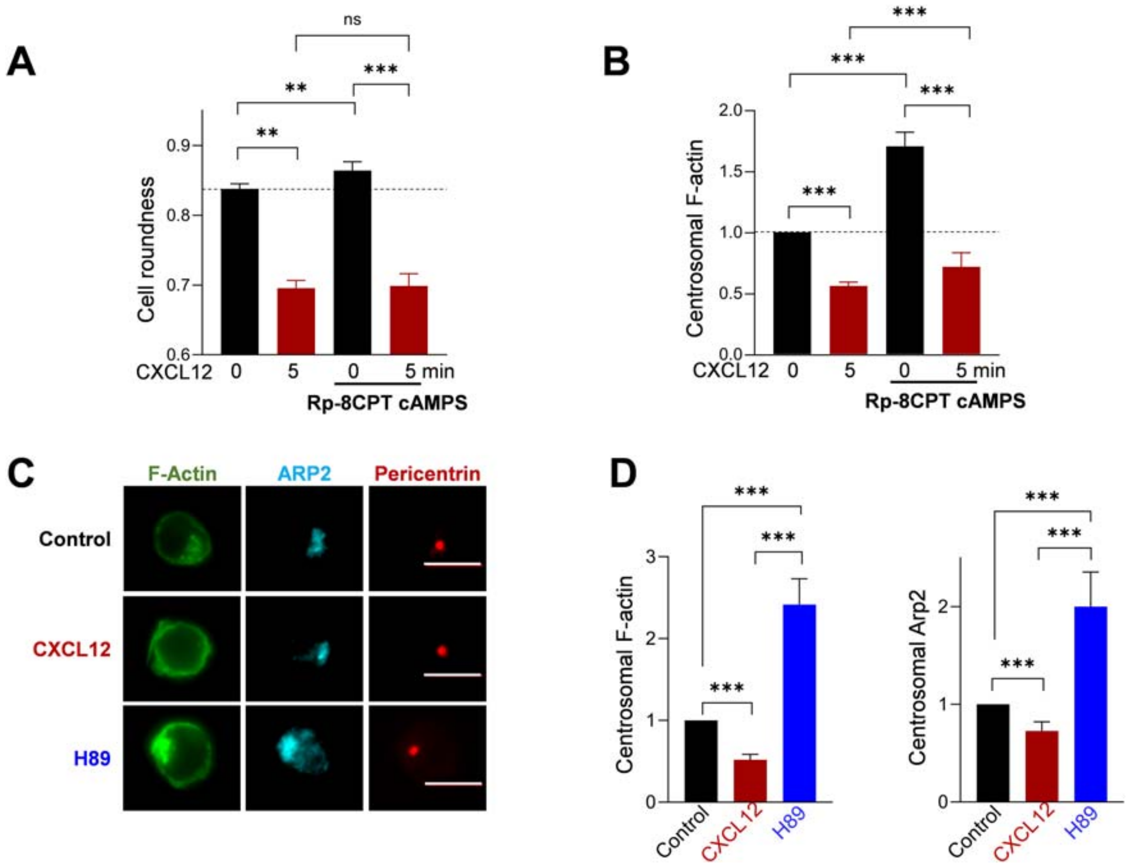

Figure EV4. Effect of Rp-8-CPT-cAMPS on cell roundness and centrosomal actin level and centrosomal Arp2 and F-Actin distribution upon CXCL12 and PKA inhibition.

(**A**) Cell roundness of CEM T cells pretreated or not with Rp-8-CPT-cAMPS and deposited on VCAM-1 or VCAM-1 + CXCL12 (1 µg/ml) for 5 min. Mean ± SE of four independent experiments, 500–1226 cells/condition/experiment. Statistics: Nonparametric ANOVA test for multiple comparisons. ***$p < 0.001$, **$p \le 0.007$. (**B**) Centrosomal actin was measured in CEM T cells pretreated or not with Rp-8-CPT-cAMPS and stimulated or not with CXCL12 (100 ng/ml) for 5 min. For each experiment, values have been normalized relative to the mean intensity of centrosomal F-actin measured in unstimulated and untreated CEM T cells. Mean ± SE of three independent experiments, 26–61 cells/condition/experiment. Statistics: Nonparametric ANOVA test for multiple comparisons. ***$p < 0.001$ (**C**) Example of F-Actin and Arp2 distributions in representative CEM T cells in control conditions (upper line), after CXCL12 stimulation (middle line) or after H89 pretreatment (lower line). Scale bar = 10 µm. (**D**) Effect of CXCL12 stimulation and PKA inhibition (H89) on F-Actin and Arp2/3 intensities at the centrosome. The quantification of the two proteins have been performed on the same cells. For each experiment and for both proteins, values have been normalized relative to the mean intensity measured in unstimulated and untreated CEM T cells. Mean ± SE of four independent experiments, 38–165 cells/condition/experiment. Statistics: Nonparametric ANOVA test for multiple comparisons. ***$p < 0.001$. Source data are available online for this figure.

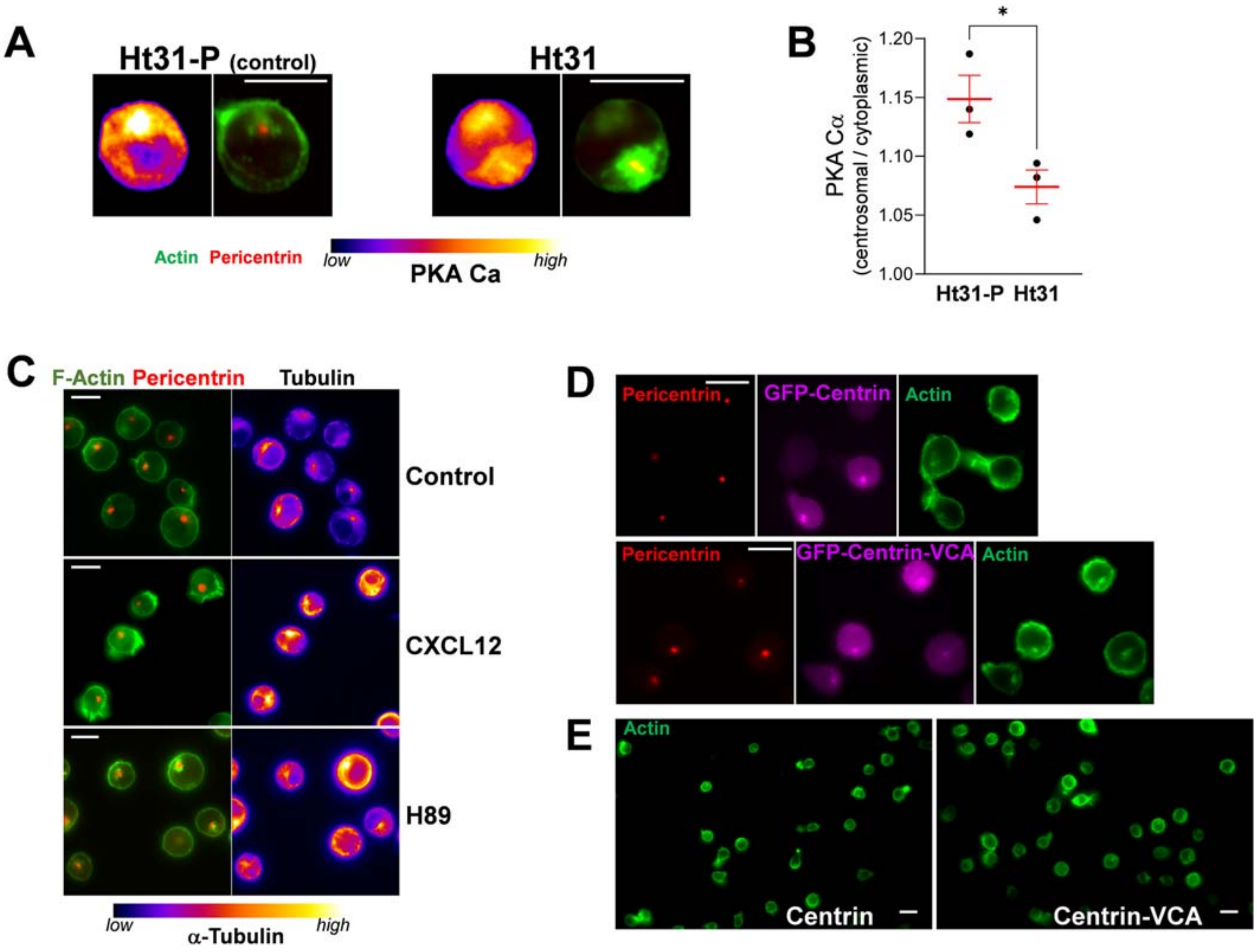

Figure EV5. PKA catalytic subunit, microtubules, and actin distributions.

(**A**) Example of PKA Cα distribution in CEM T cells treated with control peptide (Ht31-P) or with AKAP II inhibitor, Ht31. F-Actin labeled with phalloidin (green) and centrosome revealed with an antibody against pericentrin (red). Scale bar: 10 µm. (**B**) The ratio between PKA Cα intensities measured in a 3 µm diameter area centered at the centrosome with the ones measured in a similar area in the cytoplasm in cells either treated with the control peptide (Ht31-P) or with the AKAP II inhibitor (Ht31). A ratio >1 reflects an accumulation of the protein around the centrosome. Mean ± SE of three independent experiments, 12–95 cells/condition/experiment. Statistics: Paired *t*-test $*p = 0.0484$. (**C**) Examples of cytoskeleton distribution in CEM T cells in control conditions, stimulated with CXCL12, or pretreated with H89. F-Actin (green) is labeled with phalloidin, the centrosome with anti-pericentrin antibody, and microtubules with anti-α tubulin antibody. Scale bar = 10 µm. (**D**) Example of F-actin distribution (green) in CEM T cells transfected with either GFP-centrin1 or with GFP-centrin1-VCA (magenta). The centrosome is revealed with an antibody against pericentrin (red). Scale bar = 10 µm. (**E**) Example of morphology of CEM T cell transfected with either GFP-centrin1 or with GFP-centrin1-VCA. F-actin is labeled with phalloidin (green). Scale bar: 10 µm. Source data are available online for this figure.

 © The Author(s)
